# Design, Synthesis and Biological Activity Evaluation of β-Carboline Derivatives Containing Nitrogen Heterocycles

**DOI:** 10.3390/molecules29215155

**Published:** 2024-10-31

**Authors:** Guiyun Wu, Wenhang Wang, Fulian Li, Chenlu Xu, Yue Zhou, Zhurui Li, Bingqian Liu, Lihui Shao, Danping Chen, Song Bai, Zhenchao Wang

**Affiliations:** 1 Guizhou Engineering Laboratory for Synthetic Drugs, College of Pharmacy, Guizhou University, Guiyang 550025, China; gs.gywu22@gzu.edu.cn (G.W.); lflian1632562531@163.com (F.L.); dpchen@gzu.edu.cn (D.C.); 2 State Key Laboratory of Green Pesticide, Key Laboratory of Green Pesticide and Agricultural Bioengineering, Ministry of Education, Center for R&D of Fine Chemicals, Guizhou University, Guiyang 550025, China; 3 Research Center for Green Chemistry and Ecological Environment Technology, Guizhou Industry Polytechnic College, Guiyang 550008, China

**Keywords:** β-carboline derivatives, antitumor, apoptosis, ROS, cell cycle

## Abstract

A series of β-carboline derivatives containing nitrogen heterocycles were designed and synthesized. All compounds were screened for their antitumor activity against four human tumor cell lines (A549, K562, PC-3, T47D). Notably, compound *N*-(4-(morpholinomethyl)phenyl)-2-((5-(1-(3,4,5-trimethoxyphenyl)-9*H*-pyrido[3,4-b]indol-3-yl)-1,3,4-oxadiazol-2-yl)thio)acetamide (**8q**) exhibited significant inhibitory activity against PC-3 cells with an IC_50_ value of 9.86 µM. Importantly, compound **8q** effectively suppressed both the proliferation and migration of PC-3 cells. Mechanistic studies revealed that compound **8q** induced cell apoptosis and caused the accumulation of reactive oxygen species (ROS), leading to cell cycle arrest in the G0/G1 phase in PC-3 cells.

## 1. Introduction

**Figure 1:**
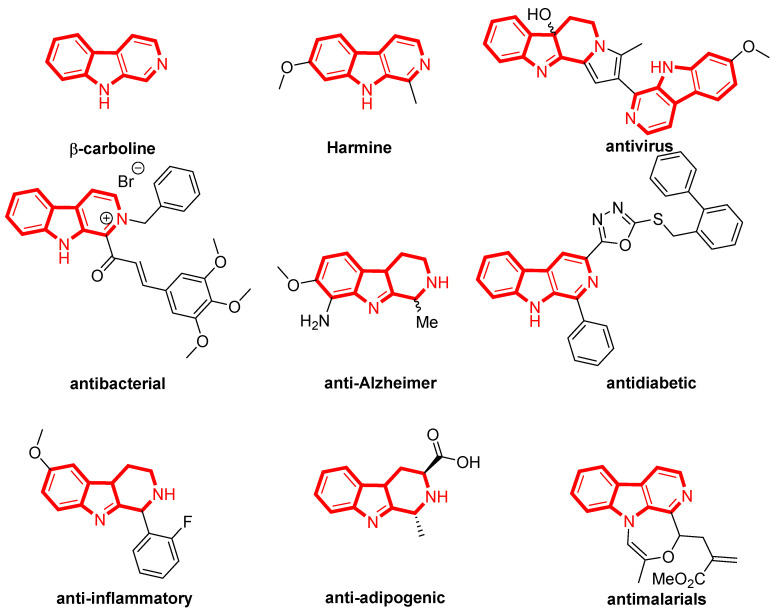
Chemical structures of β-carboline derivatives with multiple activities.

The utilization of natural products in the development of novel drugs is frequently favored due to their distinctive molecular frameworks and extensive pharmacological properties [1]. The tricyclic planar pyrido-[3,4-b]indole ring alkaloid β-carboline, which is extracted from the seeds of *Peganum harmala* L., has been documented to possess diverse pharmacological activities, particularly antitumor effects [2]. Its presence can be found in various locations, including plants, marine organisms and even human tissues [3]. The antitumor activity of β-carboline alkaloids and their derivatives is exerted through various mechanisms, including the inhibition of both topoisomerases I and II [4,5], cyclin-dependent kinases (CDKs) [6,7], IkappaB kinase (IKK) [8], Polo-like kinases (PLKs) [9], monoamine oxidase (MAO) [10,11] and DNA insertion and binding [12,13,14]. The compound under investigation exhibits a range of diverse biological activities in addition to its currently studied antitumor activity, including antibacterial [15], antiviral [16,17], anti-Alzheimer [18], antidiabetic [19], anti-inflammatory [20], anti-adipogenic [21] and antimalarial [22,23] (Figure 1).

The critical role of many heterocyclic compound systems in drug development lies in their ability to bind with other cyclic systems, thereby augmenting their activity and exhibiting potent anticancer effects against various human malignant tumor cell lines [24,25,26,27]. Oxadiazole activities include but are not limited to anticancer [28], antibacterial [29] and antifungal [30]. Due to its diverse biological activities, oxadiazole plays a crucial role in drug development. Zibotentan, an oxadiazole-based hybrid drug, is currently undergoing clinical trials [31]. Additionally, 1,3,4-oxadiazole is partially present in several commercially available drugs such as furamizole, raltegravir and nesapidil [32]. Moreover, the piperazine ring, as of 2014, has been reported to be the fourth most common type of heterocycle in marketed drugs, and it is usually introduced into the terminal part of the drug to improve molecular water solubility [33]. Overall, heterocycles play a crucial role in drug molecules.

The unique spatial structure of natural products has always made them excellent scaffolds for targeting various molecular targets, ensuring high-quality outcomes [34]. β-Carboline has biological activity due to its three parallel rings forming a special planar aromatic ring, which makes it easy to embed into DNA, causing DNA damage or preventing DNA repair [35,36]. The oxadiazole moiety is frequently employed in drug molecular design as a linking bridge, while the β-carboline exhibits poor water solubility [37]. Therefore, piperazine, as an end element to improve molecular water solubility, is introduced into the target molecular compound. In order to explore small molecules with higher antitumor activity in β-carboline derivatives, the Pictet–Spengler reaction to the C1 and C3 positions of β-carboline introduces different functional groups, which can enhance antitumor activity [37,38].

**Figure 2:**
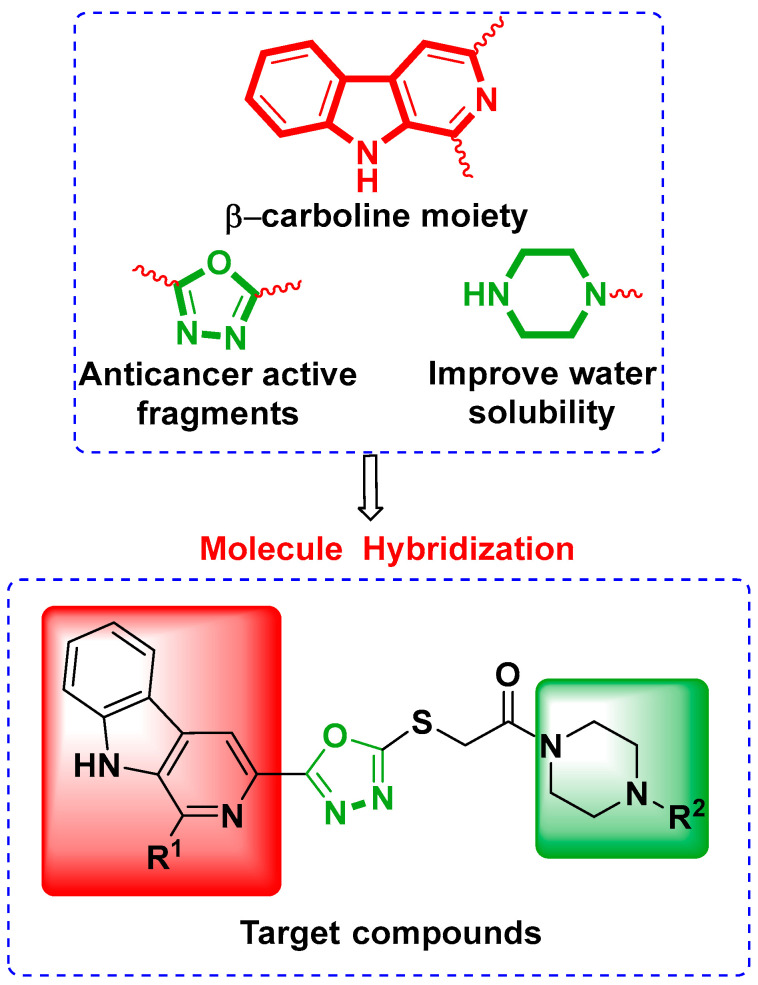
The design concept of β-carboline derivatives.

One commonly employed approach involves the derivation and modification of natural products to obtain more potent compounds. Based on the significant role of heterocycles in drug molecules, in this work, we designed, synthesized and evaluated 3-substituted nitrogen-containing heterocyclic β-carboline derivatives using a highly active fragment molecular hybridization method, with the aim of obtaining target compounds with higher activity (Figure 2).

## 2. Results and Discussion

### 2.1. Synthesis

The synthetic route of β-carboline derivatives containing nitrogen heterocycles is shown in Scheme 1. Firstly, the existing and widely existing commodity L-tryptophan and different substituted benzaldehydes are reacted through a Pictet–Spengler reaction to obtain three parallel rings, namely key intermediate **2a**. Subsequently, intermediate **5a** is obtained through the esterification, oxidation and hydrazine hydrolysis of intermediate **2a**. Intermediate **6a** is obtained through acidification [39,40], and finally, nucleophilic substitution is carried out with chloroacetylpiperazine to obtain the target compounds **7** series and **8** series [41]. Spectra of compounds (^1^H NMR, ^13^C NMR, HRMS) of series **7** and **8** are included in the Supplementary Materials (Figures S1–S103).

### 2.2. Biological Evaluation

#### 2.2.1. In Vitro Antitumor Activity of Target Compounds

To investigate the antitumor activity of β-carboline derivatives, their inhibitory ability against A549 (human lung cancer cell), K562 (human chronic myeloid leukemia cell), PC-3 (human prostate cancer cell), T47D (H]human breast cancer cell) and Hek-293T (human embryonic kidney cells) were evaluated using MTT assay according to previously reported methods [42]. Cells were purchased from the Cell Bank of the Chinese Academy of Sciences (Kunming, China). The initial screening results showed that most compounds only had poor anticancer activity (Table 1 and Table 2). From the activity data results, it could be seen that compounds with substituted R^1^ groups had significantly improved water solubility, which was the **8** series. This may be due to the appearance of substituent groups causing a change in steric hindrance. Subsequently, the rescreening process focused exclusively on compounds **8g** and **8q**, which belong to the more soluble **8** series. As shown in Table 3, only compounds **8g** and **8q** exhibit activity below 10 μM. The following structure–activity relationship is obtained from Table 3: (1) Substitution at the C-1 position is necessary. During the experiment, the water solubility of the **8** series was generally higher than that of the **7** series, possibly due to the change in steric hindrance caused by the introduction of functional groups. (2) The introduction of nitrogen-containing heterocycles is necessary. For example, compounds **8g** and **8q**, in addition to the oxadiazole bridge, also connected to the piperazine ring and the morpholine ring, respectively. They displayed promising antitumor activity against PC-3 cells, with IC_50_ values of 9.56 μM and 9.86 μM, respectively. However, the antitumor activity of **8k** (substituted with p-fluoroaniline) without either a piperazine ring or a morpholine ring was greatly reduced, and its inhibitory activity on PC-3 was greater than 40%. The results from Table 1 demonstrate that the majority of compounds belonging to series **7** and **8** exhibited a limited persistent impact on normal Hek-293T cells. Under microscopy, compound **8g** was observed to precipitate crystals, potentially compromising the accuracy of antitumor activity measurements. Consequently, compound **8q** was selected for further investigation, and detailed studies were conducted to elucidate its antitumor mechanisms in PC-3 cells.

#### 2.2.2. Colony-Forming Assay and Wound-Healing Assay

**Figure 3:**
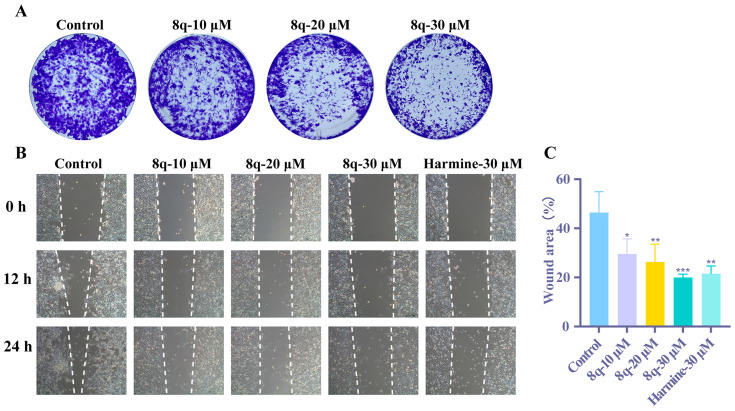
(**A**) Compound **8q** inhibited the colony formation of PC-3 cells (scale bar: 100 μm). (**B**) The effect of compound **8q** at 10 μM, 20 μM and 30 μM concentration and Harmine at 30 μM concentration on the migration of PC-3 cells. (**C**) Quantitative analysis of PC-3 cells’ migration rate using Image J software (2.1.0/1.53c). One-way ANOVA followed by Dunnett’s multiple-comparisons test was performed using GraphPad Prism 9 (* *p* < 0.05, ** *p* < 0.01, *** *p* < 0.001).

Upon conducting preliminary phenotype experiments, it was objectively observed that compound **8q** exhibited inhibitory effects on both the proliferation and migration of PC-3 cells. As evident from the results depicted in Figure 3A, the inhibitory action of compound **8q** on cancer cell proliferation occurred in a concentration-dependent manner. Given that cell migration plays a pivotal role in tumor cell invasion [43], to further validate the impact of compound **8q** on PC-3 cell motility, a wound healing assay was conducted. The data depicted in Figure 3B demonstrated that treatment with compound **8q** resulted in a concentration-dependent inhibition of PC-3 cell migration. Specifically, after 24 h of treatment, the migration rates at various concentrations were as follows: 29.56% at 10 μM, 26.32% at 20 μM, and 19.99% at 30 μM, compared to the control group rate of 46.41%. Notably, the migration rate of Harmine at a concentration of 30 μM was slightly lower, at 21.51%, than the maximum concentration of compound **8q**. These findings underscore the potent inhibitory effect of compound **8q** on the motility of PC-3 cells.

#### 2.2.3. Cell Apoptosis Analysis and Intracellular ROS Assay

**Figure 4:**
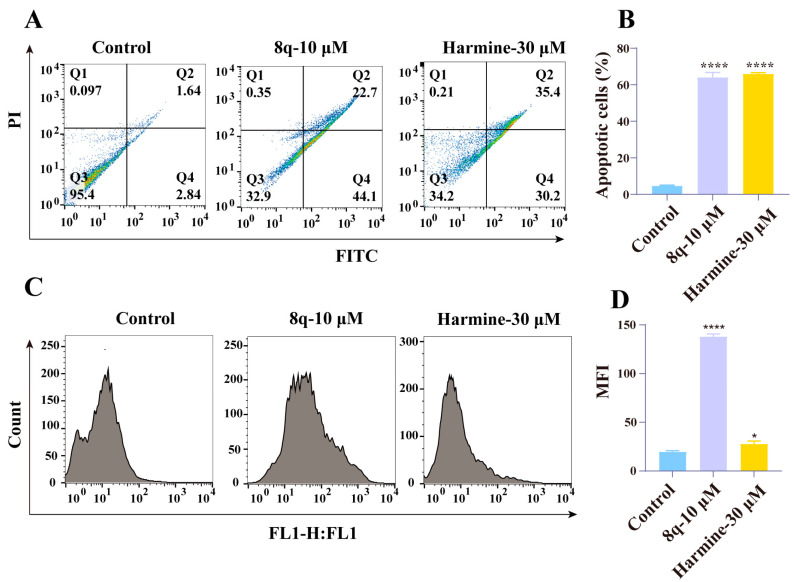
(**A**) Compound **8q** at 10 μM and Harmine at 30 μM concentrations induced apoptosis of PC-3 cells. (**B**) Quantitative analysis of PC-3 cell apoptosis using Flow Jo-V10 software. One-way ANOVA followed by Dunnett’s multiple-comparisons test was performed using GraphPad Prism 9 (**** *p* < 0.0001). (**C**) Compound **8q** at 10 μM concentration and Harmine at 30 μM increased the level of reactive oxygen species in PC-3 cells. (**D**) Quantitative analysis of reactive oxygen species in PC-3 cells using Flow Jo-V10 software. One-way ANOVA followed by Dunnett’s multiple-comparisons test was performed using GraphPad Prism 9 (* *p* < 0.05, **** *p* < 0.001).

Apoptosis is an important target for antitumor therapy. To explore the antitumor mechanism of compound **8q**, cell apoptosis was detected using flow cytometry. As shown in Figure 4A, at a concentration of 10 μM, compound **8q** caused a total apoptosis rate of 63.23% in PC-3 cells, which was equivalent to the apoptosis rate of a positive-drug-Harmine (65.96%) treatment at a concentration of 30 μM. Moreover, reactive oxygen species (ROS) play a significant role in cell growth, differentiation and apoptosis. Excessive ROS can cause oxidative damage and lead to cell apoptosis [44]. After treatment with compound **8q** at a concentration of 10 μM for 24 h, it was observed that the mean fluorescence intensity (MFI) value increased to 138; in comparison, the negative control exhibited an MFI value of 19.8, while the positive control, Harmine (30 μM), demonstrated an MFI value of 37.9 (Figure 4B). The results showed that compound **8q** at low concentrations could cause an increase in ROS in PC-3 cells, leading to cell apoptosis.

#### 2.2.4. Cell Cycle Assay and Western Blot

**Figure 5:**
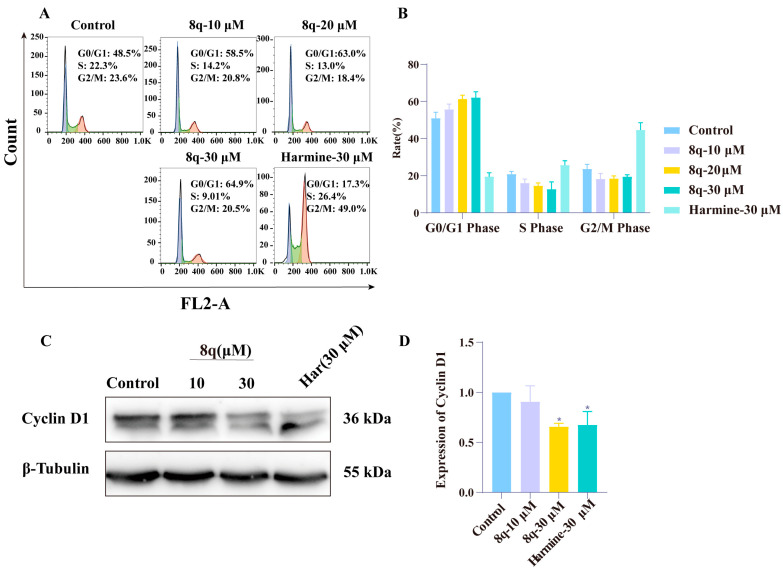
(**A**) Flow cytometry detection of the effect of compound **8q** on the distribution of PC-3 cell cycle at concentrations of 10 μM, 20 μM and 30 μM. (**B**) Quantitative analysis of cell cycles in PC-3 cells using Flow Jo-V10 software. β-Tubulin as an internal reference. One-way ANOVA followed by Dunnett’s multiple-comparisons test was performed using GraphPad Prism 9. (**C**) Western blot analysis of the effect of **8q** on the expression of Cyclin D1 in cells. (**D**) Analyzed protein grayscale values using Image J software (2.1.0/1.53c). β-Tubulin as an internal reference. One-way ANOVA followed by Dunnett’s multiple-comparisons test was performed using GraphPad Prism 9 (* *p* < 0.05).

To analyze the effect of compound **8q** on cell cycle, the cycle of PC-3 cells was detected after being treated with compound **8q** for 24 h (Figure 5). In the negative control group, the cycle showed a normal distribution, with distributions of 50.6%, 20.8% and 23.6% in G0/G1, S and G2/M phases, respectively. However, in the dosing group, the concentration of compound **8q** was 10 μM, 20 μM and 30 μM, and the distribution of the G0/G1 phase gradually increased, with values of 55.66%, 60.83% and 62.03%, respectively. The results indicated that compound **8q** arrested the PC-3 cell cycle in the G0/G1 phase. In addition, Cyclin D1, a cell cycle-related protein, is a key regulator of cycle progression, mainly regulating G1/S phase transition. Meanwhile, it is upregulated in various cancers. The Western blot assay result showed that after treating cells with compound **8q** at a concentration of 30 μM, the expression of Cyclin D1 was significantly downregulated. In summary, compound **8q** can arrest cells in the G0/G1 phase.

## 3. Conclusions

Inspired by the unique molecular structure and extensive pharmacological activity of β-carboline, a series of β-carboline derivatives containing oxadiazole and piperazine were designed, synthesized and screened for antitumor activity against four human tumor cell lines (A549, K562, PC-3, T47D). It was found that the substitution position and substituent groups of β-carboline derivatives were closely related to biological activity. The literature suggests that the incorporation of appropriate substituents at C-3 can enhance the antitumor activity of the compounds [45]. Ikeda et al. [46] conducted a synthesis of C-3-substituted β-carboline, and subsequently evaluated the antitumor efficacy of all synthesized compounds against HeLa S-3, Sarcoma 180 and 293T cancer cell lines using MTT assay. Most hybrids exhibited IC_50_ values ranging from 0.032 to 16 μM, indicating significant potency. Therefore, the antitumor activity of β-carboline was investigated by incorporating oxadiazole and piperazine moieties at the C-3 position of β-carboline using active splicing principles. The activity results indicated that most compounds exhibit lower antitumor activity due to their poor water solubility. Among all the target compounds, series **8** compounds containing 3,4,5-trimethoxyphenyl exhibited higher antitumor activity compared to series **7** compounds. These results indicate that the inclusion of 3,4,5-trimethoxyphenyl can enhance the water solubility of this compound series. Notably, only compounds **8g** and **8q** demonstrated significant antitumor activity. It is worth mentioning that compound **8q** has considerable antitumor activity against K562, PC-3 and T47D, with IC_50_ values of 11.17 µM, 9.86 µM and 9.96 µM, respectively. Therefore, PC-3 cells were chosen for further mechanistic studies. The colony formation and migration assay indicated that compound **8q** could inhibit the proliferation and motility of PC-3 in a dose-dependent manner. Exposure of PC-3 cells to 10 μM compound **8q** resulted in the induction of apoptosis and ROS accumulation. Meanwhile, compound **8q** could arrest the cell cycle in the G0/G1 phase. A Western blot assay demonstrated that compound **8q** downregulated the expression of oncoprotein Cyclin D1, confirming that compound **8q** arrested it in G0/G1 phase. However, further experimental exploration is needed to identify the target pathway, and the structure needs to be further optimized to improve its water solubility and antitumor activity.

## Figures and Tables

**Scheme 1:**
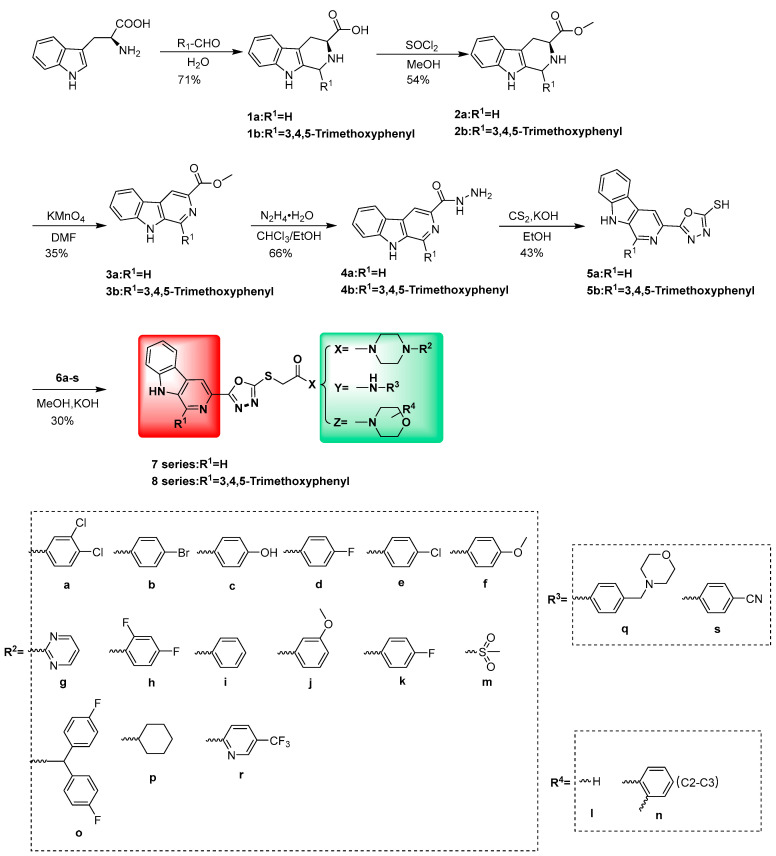
Synthetic route of target compounds **7** and **8** series.

**Table 1:** In vitro cytotoxicity (10 μM) of the compound **7** series.

Compds.	Inhibitory Activity ^a^/% at 10 μM				
	PC-3	A549	K562	T47D	Hek-293T
**7a**	29.58 ± 0.25	0.72 ± 1.63	0.85 ± 1.01	32.30 ± 3.36	18.56 ± 0.71
**7b**	29.09 ± 0.27	13.61 ± 7.88	8.98 ± 3.13	42.62 ± 1.65	33.29 ± 0.80
**7c**	22.69 ± 2.19	16.75 ± 2.39	18.08 ± 2.63	40.21 ± 0.90	47.29 ± 4.59
**7d**	26.47 ± 5.76	11.24 ± 0.69	17.82 ± 3.97	25.41 ± 4.82	36.90 ± 1.74
**7e**	24.97 ± 4.06	17.98 ± 2.06	12.65 ± 4.72	47.69 ± 3.36	26.36 ± 1.35
**7f**	11.54 ± 2.78	13.54 ± 6.97	21.49 ± 3.02	42.35 ± 4.23	56.84 ± 2.21
**7g**	14.81 ± 2.23	/ ^c^	0.05 ± 2.99	42.52 ± 3.19	34.11 ± 6.80
**7h**	10.65 ± 2.68	2.16 ± 3.56	8.23 ± 4.12	46.42 ± 2.12	47.51 ± 0.78
**7i**	35.65 ± 1.89	5.20 ± 7.21	23.60 ± 3.59	54.25 ± 3.88	84.14 ± 0.79
**7j**	14.72 ± 4.49	34.04 ± 0.87	13.61 ± 1.21	39.88 ± 3.50	13.44 ± 0.68
**7k**	35.84 ± 4.09	10.11 ± 6.19	12.24 ± 0.18	47.52 ± 1.39	30.77 ± 2.59
**7l**	39.56 ± 5.06	12.23 ± 6.14	9.28 ± 2.51	19.32 ± 5.03	11.20 ± 7.91
**7m**	20.01 ± 3.22	14.72 ± 3.64	32.99 ± 1.83	25.16 ± 4.10	29.48 ± 3.33
**7n**	36.00 ± 3.68	8.72 ± 2.57	35.08 ± 3.12	47.75 ± 0.82	61.79 ± 1.57
**7o**	5.36 ± 3.63	/	1.26 ± 3.66	25.01 ± 1.43	35.33 ± 3.81
**7p**	51.25 ± 3.11	17.00 ± 2.10	8.68 ± 6.83	50.69 ± 5.79	61.48 ± 3.24
**7q**	29.50 ± 1.38	8.67 ± 0.64	17.01 ± 1.86	46.42 ± 2.86	32.30 ± 2.21
**7r**	43.98 ± 2.62	3.03 ± 5.83	17.22 ± 2.35	38.40 ± 5.85	0.74 ± 3.77
Harmine ^b^	14.46 ± 1.42	30.99 ± 2.81	36.02 ± 2.77	51.28 ± 4.58	29.10 ± 3.19
5-Fluorouracil	— ^d^	40.58 ± 2.04	—	—	—
Gefitinib	—	37.06 ± 0.50	—	—	—

^a^ Average of three replicates. ^b^ Harmine used as a positive control. ^c^ “/” means no anticancer activity. ^d^ indicates the unmeasured values.

**Table 2:** In vitro cytotoxicity (10 μM) of the compound **8** series.

Compds.	Inhibitory Activity ^a^/% at 10 μM				
	PC-3	A549	K562	T47D	Hek-293T
**8a**	7.73 ± 1.33	7.02 ± 4.02	21.02 ± 1.65	7.49 ± 3.25	29.40 ± 2.16
**8b**	8.91 ± 5.34	5.03 ± 5.42	10.85 ± 2.32	10.64 ± 1.16	38.35 ± 3.85
**8d**	17.86 ± 2.80	10.08 ± 4.44	8.62 ± 5.59	/ ^c^	36.92 ± 2.63
**8e**	26.85 ± 4.39	26.77 ± 2.60	54.35 ± 2.32	/	23.36 ± 3.77
**8f**	34.50 ± 0.68	13.23 ± 0.56	7.87 ± 5.82	6.91 ± 5.13	20.21 ± 4.34
**8g**	45.44 ± 0.82	35.75 ± 1.17	6.38 ± 0.52	40.65 ± 4.48	28.96 ± 4.56
**8h**	12.21 ± 1.13	17.88 ± 2.91	2.46 ± 1.78	/	29.56 ± 3.04
**8i**	21.31 ± 4.80	18.41 ± 1.80	25.50 ± 2.72	0.56 ± 3.38	25.55 ± 3.44
**8j**	15.74 ± 3.93	0.13 ± 4.62	24.96 ± 3.95	/	20.37 ± 1.51
**8k**	44.72 ± 1.82	33.22 ± 0.45	33.46 ± 8.79	6.94 ± 3.38	29.62 ± 2.33
**8m**	46.58 ± 0.35	2.70 ± 1.78	30.19 ± 2.16	/	20.97 ± 2.61
**8n**	31.96 ± 1.17	7.62 ± 3.84	38.23 ± 3.73	25.64 ± 3.79	35.54 ± 1.75
**8p**	19.12 ± 2.79	8.84 ± 0.36	44.59 ± 4.10	54.16 ± 4.87	28.93 ± 3.22
**8q**	46.07 ± 0.74	32.86 ± 4.30	25.65 ± 14.89	14.53 ± 7.43	54.76 ± 0.99
**8s**	41.45 ± 0.19	10.72 ± 5.29	7.59 ± 2.67	/	60.65 ± 5.81
Harmine ^b^	14.46 ± 1.42	31.23 ± 2.67	36.02 ± 2.77	48.76 ± 2.37	29.10 ± 3.19
5-Fluorouracil	— ^d^	40.58 ± 2.04	—	—	—
Gefitinib	—	37.06 ± 0.50	—	—	—

^a^ Average of three replicates. ^b^ Harmine used as a positive control. ^c^ “/” means no anticancer activity. ^d^ indicates the unmeasured values.

**Table 3:** In vitro cytotoxicity (IC_50_) of the compounds.

Compds.	IC_50_ ^a^/(Mean ± S.D., μM)			
	PC-3	A549	K562	T47D
**8g**	9.56 ± 1.43	>40	>40	>40
**8q**	9.86 ± 1.03	26.18 ± 1.51	11.71 ± 0.21	9.96 ± 0.63
Harmine ^b^	24.43 ± 4.45	25.92 ± 4.78	15.68 ± 2.27	38.45 ± 1.17

^a^ Average of three replicates. ^b^ Harmine used as a positive control.

## Data Availability

Data will be made available on request.

## Supplementary Materials

The following supporting information can be downloaded at: https://www.mdpi.com/article/10.3390/molecules29215155/s1, Figures S1–S103: Spectra of compounds (^1^H NMR, ^13^C NMR, HRMS). Refs. [47,48] are cited and included in Supplementary Materials.
