# Supplementary material for: Design, Synthesis and Biological Activity Evaluation of β-Carboline Derivatives Containing Nitrogen Heterocycles
Source: Molecules. 2024 Oct 31;29(21):5155. doi: 10.3390/molecules29215155 (PMC11547513; doi:10.3390/molecules29215155)
Supplement: Supplementary file 1 [file molecules-29-05155-s001.zip › molecules-3238866-supplementary.pdf]

Design, Synthesis and Biological Activity Evaluation of β-Carboline Derivatives Containing Nitrogen Heterocycles

Guiyun Wu [1,†], Wenhang Wang [1,†], Fulian Li [1], Chenlu Xu [1], Yue Zhou [1], Zhurui Li [1], Bingqian Liu [1], Lihui Shao [2], Danping Chen [1], Song Bai [3,*] and Zhenchao Wang [1,2,*]

[1] College of Pharmacy, Guizhou Engineering Laboratory for Synthetic Drugs, Guizhou University,
Guiyang 550025, China; gs.gywu22@gzu.edu.cn (G.W.);
whwang2302@163.com (W.W.); lflian1632562531@163.com (F.L.);
leooswin@163.com (C.X.); yzhou8@gzu.edu.cn (Y.Z.);
zrli3@gzu.edu.cn (Z.L.); nqliu@gzu.edu.cn (B.L.); dpchen@gzu.edu.cn (D.C.)

[2] State Key Laboratory of Green Pesticide, Key Laboratory of Green Pesticide and Agricultural
Bioengineering, Ministry of Education, Center for R&D of Fine Chemicals, Guizhou University,
Guiyang 550025, China; slihuistar@163.com

[3] Research Center for Green Chemistry and Ecological Environment Technology, Guizhou Industry Polytechnic College, Guiyang 550008, China

* Correspondence: basonmail@163.com (S.B.); zcwang@gzu.edu.cn (Z.W.)

† These authors contributed equally to this work.

Content

1. Chemistry..3
1.1. The general preparation process of **1a**, **1b**..3
1.2. The general preparation process of **2a**, **2b**..4
1.3. The general preparation process of **3a**, **3b**..4
1.4. The general preparation process of **4a**, **4b**..4
1.5. The general preparation process of **6a- 6s**...5
1.6. The general preparation process of target compounds **7** and **8** series.................................6
2. Exploration of *in vitro* antitumor mechanisms 23
2.1. Colony formation assay..23
2.2. Wound healing assay...23
2.3. The effect of compound **8q** on cell apoptosis..24
2.4. The effect of compound **8q** on cellular reactive oxygen species levels 24
2.5. Cell cycle arrest of compound **8q** on PC-3...25
2.6. Effects of **8q** on expression of cell cycle related protein...25
3. Spectra of compounds **7** series and **8** series...26

1. Chemistry

Unless otherwise specified, all reagents are purchased and of analytical grade. The ^{1}H NMR and ^{13}C NMR are characterized using JEOLECX-500 NMR (JEOL, Japan), with DMSO-d_6 as the solvent and TMS as the internal standard. The corresponding chemical shifts and coupling constants (*J*) were recorded in parts per million (ppm) and hertz (Hz), respectively. The title compounds were dissolved in methanol and analyzed by HRMS-ESI spectra on a Thermo Scientific Q Exactive series instrument. The melting point is characterized by X-4D. Thin-layer chromatography (TLC) is used to monitor the reaction process under ultraviolet light.

1.1 The general preparation process of 1a, 1b.

L-tryptophan (10.2 g, 49.94 mmol) and NaOH (2 g, 49.94 mmol) were weighed and placed in a 250 mL flask. 80 mL of water was added and stirred at room temperature. After the solution was clarified, formaldehyde (1.65 g, 54.94 mmol) was added to the system and reacted at room temperature for 3.5 h, followed by a heated reflux reaction for 2.5 h. TLC was used to monitor reaction. After the reaction was completed, removed the flask and cooled it to room temperature. The pH was adjusted to around 6 with concentrated HCl and placed it in the refrigerator overnight. After the solid was filtered out, it was washed three times with water, then rinsed again with acetone, and dried to obtain intermediate **1a.** The synthesis of **1b** involved replacing the solvent with glacial acetic acid. The crude products **1a** and **1b** were not purified and were directly used for the next step.

1.2 The general preparation process of 2a, 2b.

1a or **1b** (4.5 g, 20.91 mmol) and an appropriate amount of anhydrous methanol were added to a 250 mL flask. Dichlorosulfoxide (6.74 mL, 56.66 mmol) was slowly added dropwise under ice bath conditions. After the dripping was completed, the temperature was raised to reflux, and TLC was used to track and monitor the reaction. After the reaction was completed, cooled to room temperature, and let it sit overnight in the refrigerator. A white needle like solid was precipitated, then filtered and dried to obtain pure intermediate **2a**, **2b**.

1.3 The general preparation process of 3a, 3b.

Intermediate **2a** or **2b** (1.4 g, 6.11 mmol) was placed in a 100 mL flask, and anhydrous DMF was added under ice cooling conditions. Potassium permanganate (0.97 g, 6.11 mmol) was added in batches and reacted overnight. When the reaction was completed, the reaction solution was filtered and poured into ice water and stirred for 10 min, then placeed it in the refrigerator and let it stand for 6 h to precipitate yellow solids. Intermediate **3a** and **3b** was obtained by filtration and drying.

1.4 The general preparation process of 4a, 4b.

Intermediate **3a** or **3b** (0.25 g, 1.11 mmol) was placed in a 25 mL flask, and a mixture of chloroform and hydrazine hydrate was added in a ratio of 1: 0.8. The mixture was then heated and refluxed for 6 h. After the complete reaction, the solid was retained and rinsed with methanol before drying to obtain the compound intermediate **4a** and

4b.

1.5 The general preparation process of 5a, 5b.

A mixture of 10 mL anhydrous ethanol and KOH (0.097 g, 1.73 mmol) was added to a 50 mL dry round bottom flask, and intermediate **4a** or **4b** (0.26 g, 1.15 mmol) was added under ice bath conditions. Then, CS_2 (0.44 g, 5.77 mmol) solution was added dropwise, and the reaction system was heated to reflux. TLC was used to monitor the reaction process. After completion (as determined by TLC), the solvent was removed by vacuum rotary evaporation, and an appropriate amount of water was added to dissolve the obtained solid. The pH was adjusted to around 3 using 10% hydrochloric acid. Intermediate **5a** and **5b** was obtained by vacuum filtration and overnight drying in a vacuum drying oven.

1.6 The general preparation process of 6a- 6s.

N-substituted piperazines (0.15 g, 0.83 mmol) were dissolved in dichloromethane (DCM) and placed in a dry and clean round bottom flask. The mixture was cooled to 0 °C. Subsequently, potassium carbonate (0.17 g, 1.25 mmol) was added to the mixed solution, followed by dropwise addition of chloroacetyl chloride (0.09 g, 0.83 mmol) dissolved in DCM. After complete addition of chloroacetyl chloride, the system temperature was raised to room temperature to achieve complete conversion of piperazine (as monitored by TLC point plate, usually lasting for 30 min-1 hour). After the reaction was completed, an appropriate amount of water was added to the solution. Finally, the organic phase was retained and anhydrous sodium sulfate was added to stand overnight to remove excess moisture. Then, anhydrous sodium sulfate was used

to remove excess water, and the solvent was removed under reduced pressure to obtain intermediate **6a- 6s**.

1.7 The general preparation process of target compounds 7 and 8 series.

Intermediate **4a** or **4b** (0.04 g, 0.15 mmol), intermediate **6a- 6s** (0.036 g, 0.15 mmol), and potassium carbonate (0.041 g, 0.3 mmol) were weighed and placed in a 25 mL flask. 3 mL of methanol was added as a solvent and stirred at room temperature for 4 - 8 h. After the TLC monitoring reaction was completed, the final target compounds were obtained by filtration. The crude solid was purified by recrystallization with methanol or silica gel column chromatography using methanol: dichloromethane (20 : 1).

2-((5-(9*H*-pyrido[3,4-b]indol-3-yl)-1,3,4-oxadiazol-2-yl)thio)-1-(4-(3,4-dichlorophenyl)piperazin-1-yl)ethan-1-one (7a):

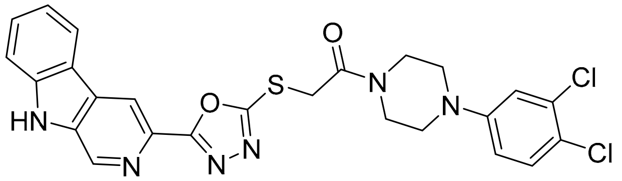

Light yellow solid, m.p.168.3−170.9 °C, yield 60%; ^{1}H NMR (400 MHz, DMSO-d_6) δ 12.08 (s, 1H), 9.00 (d, J = 20.8 Hz, 2H), 8.44 (d, J = 7.9 Hz, 1H), 7.65 (dt, J = 20.8, 8.0 Hz, 2H), 7.42 (d, J = 9.0 Hz, 1H), 7.33 (t, J = 7.4 Hz, 1H), 7.18 (d, J = 2.9 Hz, 1H), 6.97 (dd, J = 9.0, 2.9 Hz, 1H), 4.65 (s, 2H), 3.73 – 3.66 (m, 2H), 3.64 (t, J = 5.3 Hz, 2H), 3.23 (t, J = 5.3 Hz, 4H); ^{13}C NMR (101 MHz, DMSO-d6) δ 166.26, 165.33, 150.80, 141.57, 137.28, 135.03, 134.86, 132.02, 131.88, 131.02, 129.48, 128.42, 122.91, 121.09, 120.72, 120.43, 117.05, 116.04, 115.47, 112.90, 49.06, 47.71, 45.35, 41.74, 37.06; HRMS (ESI) m/z [M+H]+calcd for C25H21Cl2N6O2S : 539.0824, found: 539.0825.

2-((5-(9*H*-pyrido[3,4-b]indol-3-yl)-1,3,4-oxadiazol-2-yl)thio)-1-(4-(4-

bromophenyl)piperazin-1-yl)ethan-1-one (7b):

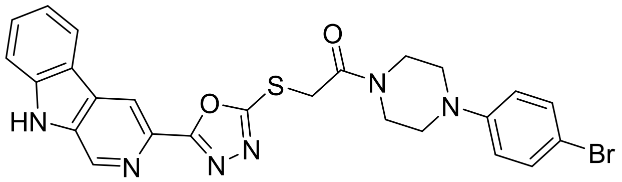

Light yellow solid, m.p.275.4‒277.7 °C, yield 70%; ^{1}H NMR (400 MHz, DMSO-d_6) δ 12.08 (s, 1H), 9.05 – 8.96 (m, 2H), 8.44 (d, *J* = 7.9 Hz, 1H), 7.70 – 7.60 (m, 2H), 7.40 – 7.31 (m, 3H), 6.96 – 6.91 (m, 2H), 4.65 (s, 2H), 3.70 (t, *J* = 5.2 Hz, 2H), 3.65 (t, *J* = 5.2 Hz, 2H), 3.27 (t, *J* = 5.2 Hz, 2H), 3.17 (t, *J* = 5.4 Hz, 2H); ^{13}C NMR (101 MHz, DMSO-d_6) δ 166.28, 165.26, 163.84, 150.28, 141.55, 137.29, 134.89, 132.05, 131.93, 129.43, 128.40, 122.90, 121.09, 120.71, 118.18, 115.46, 112.88, 110.95, 48.52, 48.17, 45.51, 41.88, 37.08; HRMS (ESI) m/z [M+H]$^+$calcd for $C_{25}H_{22}BrN_6O_2S$: 549.0708, found: 549.0709.

2-((5-(9*H*-pyrido[3,4-b]indol-3-yl)-1,3,4-oxadiazol-2-yl)thio)-1-(4-(4-hydroxyphenyl)piperazin-1-yl)ethan-1-one (7c):

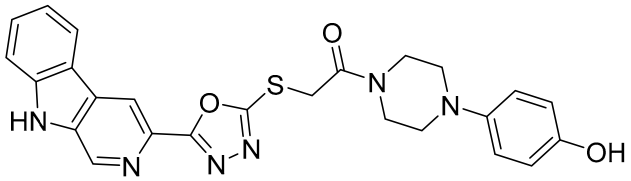

Light brown solid, m.p.163.9‒165.8 °C, yield 38%; ^{1}H NMR (400 MHz, DMSO-d_6) δ 9.04 (d, *J* = 1.2 Hz, 1H), 8.98 (d, *J* = 1.2 Hz, 1H), 8.44 (d, *J* = 7.9 Hz, 1H), 7.69 (d, *J* = 8.2 Hz, 1H), 7.63 (ddd, *J* = 8.3, 6.8, 1.2 Hz, 1H), 7.33 (td, *J* = 7.4, 6.8, 1.2 Hz, 1H), 6.85 – 6.81 (m, 2H), 6.70 – 6.65 (m, 2H), 4.64 (s, 2H), 3.72 – 3.66 (m, 2H), 3.64 (t, *J* = 5.1 Hz, 2H), 3.06 (t, *J* = 5.1 Hz, 2H), 2.96 (t, *J* = 5.2 Hz, 2H); ^{13}C NMR (101 MHz, DMSO-d_6) δ 165.15, 151.90, 144.31, 141.57, 137.30, 134.91, 131.96, 128.41, 122.92, 121.11, 120.72, 118.97, 115.99, 115.47, 112.89, 50.94, 50.52, 46.01, 42.33, 37.16; HRMS (ESI) m/z [M+H]$^+$calcd for $C_{25}H_{22}N_6O_3S$: 487.1552, found: 487.1547.

2-((5-(9*H*-pyrido[3,4-b]indol-3-yl)-1,3,4-oxadiazol-2-yl)thio)-1-(4-(4-fluorophenyl)piperazin-1-yl)ethan-1-one (7d):

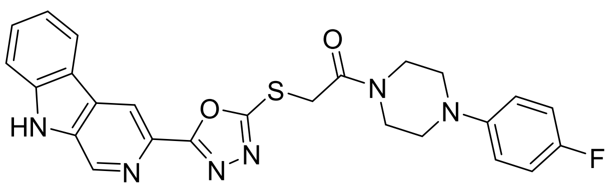

Light yellow solid, m.p.278.4–281.5 °C, yield 33%; ^{1}H NMR (400 MHz, DMSO-d_6) δ 12.08 (s, 1H), 9.03 (d, *J* = 1.1 Hz, 1H), 8.98 (d, *J* = 1.1 Hz, 1H), 8.44 (d, *J* = 7.9 Hz, 1H), 7.71 – 7.59 (m, 2H), 7.33 (ddd, *J* = 8.1, 6.9, 1.2 Hz, 1H), 7.11 – 7.04 (m, 2H), 7.00 (ddd, *J* = 7.0, 5.2, 3.1 Hz, 2H), 4.65 (s, 2H), 3.71 (t, *J* = 5.0 Hz, 2H), 3.65 (t, *J* = 5.3 Hz, 2H), 3.20 (t, *J* = 5.1 Hz, 2H), 3.09 (t, *J* = 5.3 Hz, 2H); ^{13}C NMR (101 MHz, DMSO-d_6) δ 165.15, 151.90, 144.31, 141.57, 137.30, 134.91, 131.96, 128.41, 122.92, 121.11, 120.72, 118.97, 115.99, 115.47, 112.89, 50.94, 50.52, 46.01, 42.33, 37.16; HRMS (ESI) m/z $[M+H]^+$calcd for $C_{25}H_{22}FN_6O_2S$: 489.1509, found: 489.1502.

2-((5-(9*H*-pyrido[3,4-b]indol-3-yl)-1,3,4-oxadiazol-2-yl)thio)-1-(4-(4-chlorophenyl)piperazin-1-yl)ethan-1-one (7e):

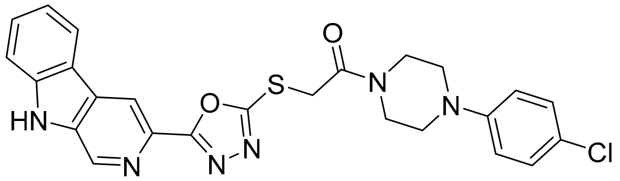

Yellow solid, m.p.246.4–248.3 °C, yield 54%; ^{1}H NMR (400 MHz, DMSO-d_6) δ 12.12 (d, *J* = 12.3 Hz, 1H), 9.03 (d, *J* = 1.2 Hz, 1H), 8.98 (s, 1H), 8.45 (dd, *J* = 8.1, 5.2 Hz, 1H), 7.70 – 7.60 (m, 2H), 7.33 (ddt, *J* = 8.0, 6.9, 1.7 Hz, 1H), 7.28 – 7.23 (m, 2H), 6.99 (dd, *J* = 9.7, 2.8 Hz, 2H), 4.65 (s, 2H), 3.71 (t, *J* = 5.2 Hz, 2H), 3.65 (t, *J* = 5.3 Hz, 2H), 3.27 (t, *J* = 5.2 Hz, 2H), 3.19 – 3.15 (m, 2H); ^{13}C NMR (101 MHz, DMSO-d_6) δ 166.29, 165.26, 163.84, 149.95, 141.56, 137.30, 134.89, 131.93, 129.44, 129.19, 128.39, 123.29, 122.91, 121.10, 120.71, 117.76, 115.47, 112.89, 48.68, 48.33, 45.55, 41.91, 37.09; HRMS (ESI) m/z $[M+H]^+$calcd for $C_{25}H_{22}ClN_6O_2S$: 505.1213, found: 505.1208.

2-((5-(9*H*-pyrido[3,4-b]indol-3-yl)-1,3,4-oxadiazol-2-yl)thio)-1-(4-(4-methoxyphenyl)piperazin-1-yl)ethan-1-one (7f):

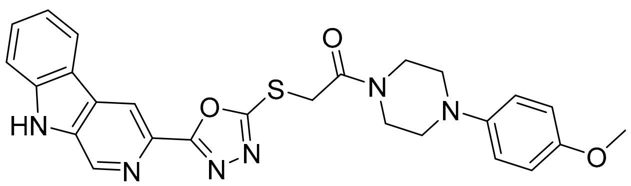

Yellow solid, m.p.190.3−191.9 °C, yield 45%; ^{1}H NMR (400 MHz, DMSO-d_6) δ 12.08 (s, 1H), 9.04 – 8.95 (m, 2H), 8.44 (d, J = 7.9 Hz, 1H), 7.70 – 7.59 (m, 2H), 7.33 (ddd, J = 7.9, 6.9, 1.2 Hz, 1H), 7.14 (t, J = 8.2 Hz, 1H), 6.56 (dd, J = 8.2, 2.3 Hz, 1H), 6.49 (t, J = 2.3 Hz, 1H), 6.41 (dd, J = 8.1, 2.3 Hz, 1H), 4.65 (s, 2H), 3.72 (s, 3H), 3.70 (t, J = 5.2 Hz, 2H), 3.64 (t, J = 5.1 Hz, 2H), 3.26 (s, 2H), 3.16 (t, J = 5.2 Hz, 2H); ^{13}C NMR (101 MHz, DMSO-d_6) δ 166.27, 165.22, 163.86, 160.69, 152.51, 141.55, 137.28, 134.89, 131.93, 130.20, 129.44, 128.40, 122.90, 120.72, 115.45, 112.88, 108.90, 105.13, 102.47, 55.38, 48.88, 48.55, 45.67, 42.05, 37.08; HRMS (ESI) m/z [M+H]$^+$calcd for $C_{26}H_{25}N_6O_3S$: 500.1709, found: 500.1707.

2-((5-(9*H*-pyrido[3,4-b]indol-3-yl)-1,3,4-oxadiazol-2-yl)thio)-1-(4-(pyrimidin-2-yl)piperazin-1-yl)ethan-1-one (7g):

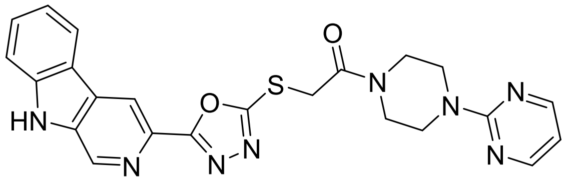

Yellow solid, m.p.212.6−213.3 °C, yield 58%; 1H NMR (400 MHz, DMSO-d6) δ 12.08 (s, 1H), 9.04 – 8.96 (m, 2H), 8.42 (dd, J = 13.7, 6.3 Hz, 3H), 7.70 – 7.60 (m, 2H), 7.32 (ddd, J = 8.0, 6.9, 1.1 Hz, 1H), 6.69 (t, J = 4.7 Hz, 1H), 4.66 (s, 2H), 3.88 (dd, J = 6.6, 3.9 Hz, 2H), 3.78 (dd, J = 6.7, 3.9 Hz, 2H), 3.66 (dd, J = 6.7, 3.8 Hz, 2H), 3.60 (dd, J = 6.7, 4.0 Hz, 2H); ^{13}C NMR (101 MHz, DMSO-d_6) δ 166.28, 165.42, 163.87, 161.55, 158.49, 141.55, 137.29, 134.86, 131.93, 129.43, 128.40, 122.91, 121.10, 120.70, 115.46, 112.88, 110.99, 45.60, 43.67, 43.42, 42.00; HRMS (ESI) m/z [M+H]$^+$calcd for $C_{23}H_{21}N_8O_2S$: 473.1508, found: 473.1511.

2-((5-(9*H*-pyrido[3,4-b]indol-3-yl)-1,3,4-oxadiazol-2-yl)thio)-1-(4-(2,4-difluorophenyl)piperazin-1-yl)ethan-1-one (7h):

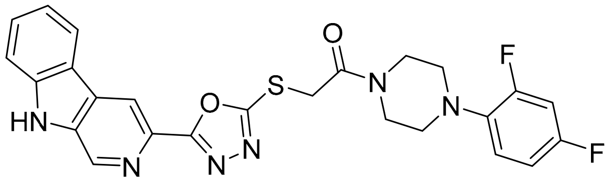

Yellow solid, m.p.148.8−149.8 °C, yield 70%; ^{1}H NMR (400 MHz, DMSO-d_6) δ 12.09 (s, 1H), 9.01 (dd, J = 20.5, 1.0 Hz, 2H), 8.44 (d, J = 7.9 Hz, 1H), 7.71 – 7.59 (m, 2H), 7.36 – 7.31 (m, 1H), 7.23

(ddd, J = 12.2, 9.0, 3.0 Hz, 1H), 7.09 (dt, J = 9.5, 4.8 Hz, 1H), 7.01 (tt, J = 8.9, 1.8 Hz, 1H), 4.65 (s, 2H), 3.72 (t, J = 5.0 Hz, 2H), 3.67 (t, J = 5.1 Hz, 2H), 3.06 (t, J = 5.0 Hz, 2H), 3.00 – 2.94 (m, 2H); ^{13}C NMR (101 MHz, DMSO-d_6) δ 166.38, 165.34, 163.86, 141.91, 137.62, 135.13, 131.76, 129.40, 128.43, 122.94, 121.19, 120.64, 115.52, 113.15, 111.77, 105.48, 105.27, 105.07, 51.08, 50.75, 46.06, 42.35, 37.27; HRMS (ESI) m/z [M+H]$^+$calcd for $C_{25}H_{21}F_2N_6O_2S$: 507.1415, found: 507.1418.

2-((5-(9*H*-pyrido[3,4-b]indol-3-yl)-1,3,4-oxadiazol-2-yl)thio)-1-(4-phenylpiperazin-1-yl)ethan-1-one (7i):

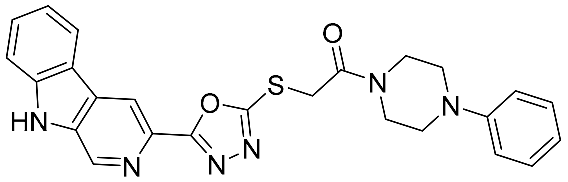

Yellow solid, m.p.148.9−150.6 °C, yield 33%; ^{1}H NMR (400 MHz, DMSO-d_6) δ 9.00 (dd, J = 18.5, 1.0 Hz, 2H), 8.44 (dd, J = 7.9, 1.1 Hz, 1H), 7.71 – 7.58 (m, 2H), 7.32 (ddd, J = 8.0, 6.9, 1.1 Hz, 1H), 7.28 – 7.21 (m, 2H), 7.01 – 6.93 (m, 2H), 6.85 – 6.79 (m, 1H), 4.65 (s, 2H), 3.71 (t, J = 5.2 Hz, 2H), 3.66 (t, J = 5.2 Hz, 2H), 3.25 (s, 2H), 3.16 (d, J = 10.4 Hz, 2H); ^{13}C NMR (101 MHz, DMSO-d_6) δ 166.28, 165.22, 163.86, 151.17, 141.56, 137.29, 134.89, 131.94, 129.49, 129.43, 128.39, 122.91, 121.10, 120.70, 119.84, 116.36, 115.46, 112.88, 48.95, 48.60, 45.72, 42.08, 37.12; HRMS (ESI) m/z [M+H]$^+$calcd for $C_{25}H_{23}N_6O_2S$: 471.1603, found: 471.1601.

2-((5-(9*H*-pyrido[3,4-b]indol-3-yl)-1,3,4-oxadiazol-2-yl)thio)-1-(4-(3-methoxyphenyl)piperazin-1-yl)ethan-1-one (7j):

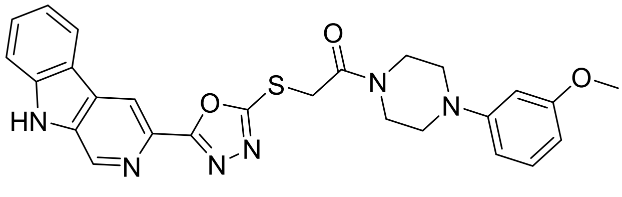

Yellow solid, m.p.156.7−157.8 °C, yield 55%; ^{1}H NMR (400 MHz, DMSO-d_6) δ 12.08 (s, 1H), 9.00 (dd, J = 19.1, 1.1 Hz, 2H), 8.44 (dt, J = 7.9, 1.1 Hz, 1H), 7.72 – 7.57 (m, 2H), 7.33 (ddd, J = 8.0, 6.9, 1.1 Hz, 1H), 7.14 (t, J = 8.2 Hz, 1H), 6.59 – 6.54 (m, 1H), 6.50 (t, J = 2.4 Hz, 1H), 6.44 – 6.39 (m, 1H), 4.65 (s, 2H), 3.73 (s, 3H), 3.69 (d, J = 5.3 Hz, 2H), 3.65 (t, J = 5.2 Hz, 2H), 3.26 (t, J = 5.2 Hz, 2H), 3.16 (t, J = 5.3 Hz, 2H); ^{13}C NMR (101 MHz, DMSO-d_6) δ 166.32, 165.27, 163.95, 160.74, 152.58,

141.60, 137.33, 134.95, 131.96, 130.27, 129.51, 128.45, 123.00, 121.15, 120.78, 115.53, 112.95, 108.96, 105.16, 102.52, 55.42, 48.93, 48.60, 45.71, 42.08, 37.22; HRMS (ESI) m/z $[M+H]^+$calcd for $C_{26}H_{25}N_6O_3S$: 501.1709, found: 501.1701.

2-((5-(9*H*-pyrido[3,4-b]indol-3-yl)-1,3,4-oxadiazol-2-yl)thio)-N-(4-fluorophenyl)acetamide (7k):

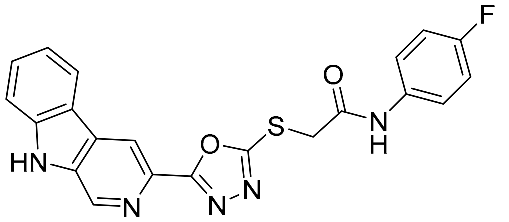

Light yellow solid, m.p.296.4−297.9 °C, yield 47%; ^{1}H NMR (400 MHz, DMSO-d_6) δ 12.08 (s, 1H), 10.58 (s, 1H), 8.94 (d, *J* = 1.0 Hz, 1H), 8.84 (d, *J* = 1.1 Hz, 1H), 8.43 (dt, *J* = 8.0, 1.0 Hz, 1H), 7.67 (dt, *J* = 8.3, 1.0 Hz, 1H), 7.61 (ddd, *J* = 8.3, 6.9, 1.2 Hz, 1H), 7.49 (ddt, *J* = 8.3, 5.5, 2.7 Hz, 2H), 7.40 (dd, *J* = 9.9, 7.7 Hz, 2H), 7.31 (ddd, *J* = 8.0, 6.9, 1.1 Hz, 1H), 4.26 (s, 2H); ^{13}C NMR (101 MHz, DMSO-d_6) δ 171.69, 163.22, 161.27, 161.24, 159.09, 141.58, 139.12, 137.80, 133.15, 131.57, 131.55, 131.13, 131.06, 129.32, 128.71, 122.96, 121.38, 120.67, 116.72, 116.53, 114.86, 112.86, 33.62; HRMS (ESI) m/z $[M+H]^+$calcd for $C_{21}H_{15}FN_5O_2S$: 420.0930, found: 420.0934.

2-((5-(9*H*-pyrido[3,4-b]indol-3-yl)-1,3,4-oxadiazol-2-yl)thio)-1-morpholinoethan-1-one (7l):

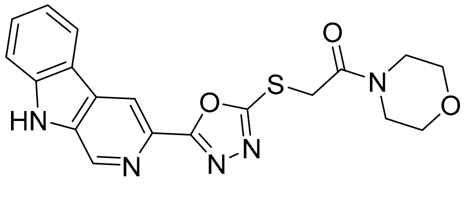

Yellow solid, m.p.228.3−229.6 °C, yield 43%; ^{1}H NMR (400 MHz, DMSO-d_6) δ 12.10 (s, 1H), 9.01 (dd, *J* = 25.0, 2.3 Hz, 2H), 8.45 (dd, *J* = 7.9, 2.3 Hz, 1H), 7.66 (dd, *J* = 20.2, 7.8 Hz, 2H), 7.33 (t, *J* = 7.5 Hz, 1H), 4.60 (d, *J* = 2.0 Hz, 2H), 3.67 (t, *J* = 4.8 Hz, 2H), 3.58 (dt, *J* = 12.9, 4.7 Hz, 4H), 3.49 (t, *J* = 4.8 Hz, 2H); ^{13}C NMR (101 MHz, DMSO-d_6) δ 166.32, 165.45, 163.91, 141.60, 137.34, 134.94, 131.94, 129.51, 128.44, 122.99, 121.14, 120.78, 115.52, 112.95, 66.43, 46.37, 42.61, 37.11; HRMS (ESI) m/z $[M+H]^+$calcd for $C_{19}H_{18}N_5O_3S$: 396.1130, found: 396.1129.

2-((5-(9*H*-pyrido[3,4-b]indol-3-yl)-1,3,4-oxadiazol-2-yl)thio)-1-(4-

(methylsulfonyl)piperazin-1-yl)ethan-1-one (7m):

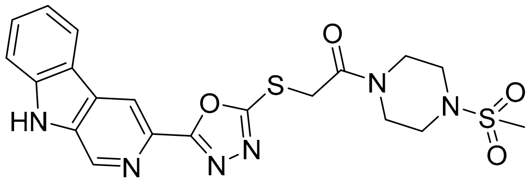

Yellow solid, m.p.174.3−175.6 °C, yield 47%; ^{1}H NMR (400 MHz, DMSO-d_6) δ 12.09 (s, 1H), 9.01 (d, J = 22.7 Hz, 2H), 8.45 (d, J = 7.9 Hz, 1H), 7.66 (dd, J = 19.5, 7.8 Hz, 2H), 7.34 (t, J = 7.5 Hz, 1H), 4.63 (s, 2H), 3.65 (dt, J = 23.1, 5.0 Hz, 4H), 3.25 (d, J = 5.1 Hz, 2H), 3.18 – 3.11 (m, 2H), 2.92 (s, 3H); ^{13}C NMR (101 MHz, DMSO-d_6) δ 166.36, 165.51, 163.84, 141.60, 137.34, 134.94, 131.94, 129.52, 128.45, 123.01, 121.14, 120.79, 115.55, 112.95, 45.83, 45.56, 41.84, 37.34, 34.76; HRMS (ESI) m/z [M+H]$^{+}$calcd for $C_{20}H_{21}N_6O_4S_2$: 473.1066, found: 473.1068.

2-((5-(9*H*-pyrido[3,4-b]indol-3-yl)-1,3,4-oxadiazol-2-yl)thio)-1-(2,3-dihydro-4H-benzo[b][1,4]oxazin-4-yl)ethan-1-one (7n):

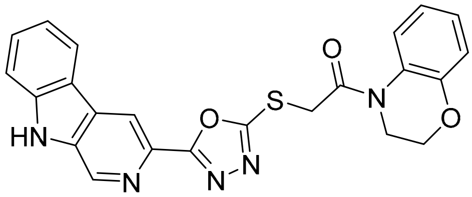

Yellow solid, m.p.232.4−232.8 °C, yield 49%; ^{1}H NMR (400 MHz, DMSO-d_6) δ 12.08 (s, 1H), 9.03 (s, 1H), 8.96 (s, 1H), 8.43 (d, J = 7.9 Hz, 1H), 7.93 (d, J = 32.8 Hz, 1H), 7.72 – 7.57 (m, 2H), 7.33 (t, J = 7.4 Hz, 1H), 7.10 (s, 1H), 6.93 (dd, J = 12.1, 7.5 Hz, 2H), 4.81 (s, 2H), 4.37 (t, J = 4.6 Hz, 2H), 3.98 (t, J = 4.6 Hz, 2H); ^{13}C NMR (101 MHz, DMSO-d_6) δ 166.33, 165.81, 163.71, 141.55, 137.30, 134.87, 131.86, 129.44, 128.39, 126.09, 122.89, 121.09, 120.71, 120.41, 117.49, 115.44, 112.88; HRMS (ESI) m/z [M+H]$^{+}$calcd for $C_{23}H_{18}N_5O_3S$: 444.1130, found: 444.1135.

2-((5-(9*H*-pyrido[3,4-b]indol-3-yl)-1,3,4-oxadiazol-2-yl)thio)-1-(4-(bis(4-fluorophenyl)methyl)piperazin-1-yl)ethan-1-one (7o):

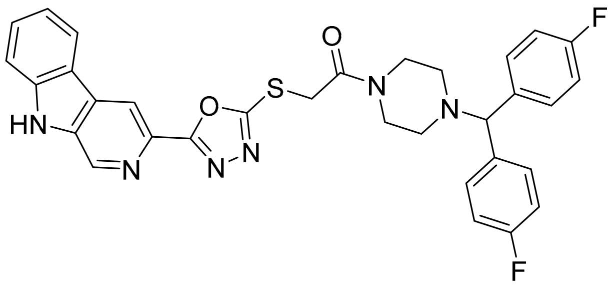

Light yellow solid, m.p.179.8−181.6 °C, yield 30%;^{1}H NMR (400 MHz, DMSO-d_6) δ 12.09 (s, 1H), 9.04 (d, J = 1.0 Hz, 1H), 8.97 (s, 1H), 8.45 (d, J = 8.0 Hz, 1H), 7.69 (d, J = 8.2 Hz, 1H), 7.63 (ddd, J = 8.2, 6.8, 1.2 Hz, 1H), 7.49 – 7.41 (m, 4H), 7.34 (ddd, J = 8.0, 6.9, 1.1 Hz, 1H), 7.19 – 7.08 (m, 4H), 4.55 (s, 2H), 4.44 (s, 1H), 3.57 (t, J = 4.9 Hz, 2H), 3.52 (t, J = 5.0 Hz, 2H), 2.36 (t, J = 5.0 Hz, 2H), 2.28 (t, J = 5.2 Hz, 2H); ^{13}C NMR (101 MHz, DMSO-d_6) δ 166.26, 165.05, 163.81, 162.80, 160.38, 141.56, 138.80, 137.29, 134.88, 131.93, 129.94, 129.86, 129.44, 128.39, 122.91, 122.80, 121.10, 120.71, 115.98, 115.77, 115.46, 112.89, 73.00, 51.70, 51.27, 45.89, 42.29, 37.07; HRMS (ESI) m/z [M+H]$^+$calcd for $C_{32}H_{27}F_2N_6O_2S$: 597.1884, found: 597.1887.

2-((5-(9*H*-pyrido[3,4-b]indol-3-yl)-1,3,4-oxadiazol-2-yl)thio)-1-(4-cyclohexylpiperazin-1-yl)ethan-1-one (7p):

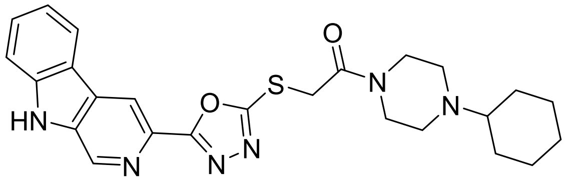

Yellow solid, m.p.158.8−161.4 °C, yield 30%; ^{1}H NMR (400 MHz, DMSO-d_6) δ 12.09 (s, 1H), 9.04 (s, 1H), 8.96 (d, J = 11.3 Hz, 1H), 8.42 (dd, J = 23.0, 7.9 Hz, 1H), 7.72 – 7.59 (m, 2H), 7.31 (dt, J = 18.2, 7.4 Hz, 1H), 4.58 (s, 2H), 3.52 (t, J = 5.0 Hz, 2H), 3.48 (t, J = 5.1 Hz, 2H), 2.59 (s, 2H), 2.30 (s, 2H), 1.82 – 1.64 (m, 4H), 1.57 (d, J = 12.2 Hz, 1H), 1.31 – 0.94 (m, 6H); ^{13}C NMR (101 MHz, DMSO-d_6) δ 166.25, 164.97, 163.85, 141.55, 137.29, 134.89, 131.93, 129.43, 128.39, 122.89, 121.09, 120.71, 115.45, 112.88, 63.23, 48.94, 48.54, 46.19, 42.52, 37.17, 28.57, 26.21, 25.69; HRMS (ESI) m/z [M+Na]$^+$calcd for $C_{25}H_{28}N_6O_2SNa$: 499.1892, found: 499.1888.

2-((5-(9*H*-pyrido[3,4-b]indol-3-yl)-1,3,4-oxadiazol-2-yl)thio)-N-(4-(morpholinomethyl)phenyl)acetamide (7q):

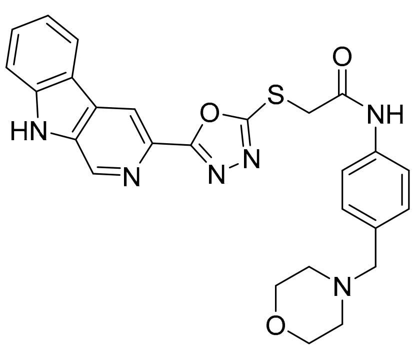

Black solid, m.p.108.9−109.4 °C, yield 65%; ^{1}H NMR (400 MHz, DMSO-d_6) δ 12.09 (s, 1H), 10.46 (s, 1H), 9.03 (s, 1H), 8.94 (s, 1H), 8.39 (d, J = 7.9 Hz, 1H), 7.72 – 7.59 (m, 2H), 7.58 – 7.52 (m, 2H), 7.33 (t, J = 7.5 Hz, 1H), 7.25 (d, J = 8.3 Hz, 2H), 4.38 (s, 2H), 3.55 (t, J = 4.7 Hz, 4H), 3.40 (s, 2H), 2.32 (t, J = 4.6 Hz, 4H); ^{13}C NMR (101 MHz, DMSO-d_6) δ 166.40, 165.27, 163.60, 141.54, 138.01, 137.28, 134.91, 133.43, 131.87, 129.93, 129.43, 128.36, 122.82, 121.06, 120.71, 119.53, 115.41, 112.89, 66.67, 66.60, 62.40, 53.71, 53.53, 37.38; HRMS (ESI) m/z [M+H]$^+$calcd for $C_{26}H_{25}N_6O_3S$: 501.1709, found: 501.1711.

2-((5-(9*H*-pyrido[3,4-b]indol-3-yl)-1,3,4-oxadiazol-2-yl)thio)-1-(4-(5-(trifluoromethyl)pyridin-2-yl)piperazin-1-yl)ethan-1-one (7r):

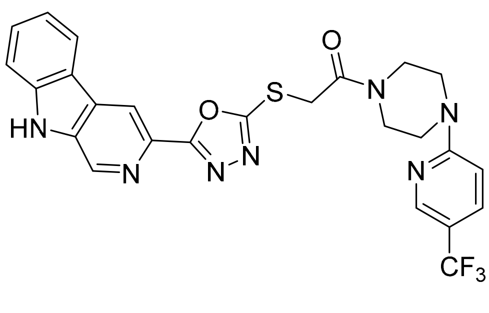

White solid, m.p.300.7−302.9 °C, yield 83%; ^{1}H NMR (400 MHz, DMSO-d_6) δ 12.08 (s, 1H), 9.05 – 9.00 (m, 1H), 8.98 (s, 1H), 8.44 (d, J = 7.5 Hz, 2H), 7.84 (dd, J = 9.1, 2.6 Hz, 1H), 7.70 – 7.59 (m, 2H), 7.33 (t, J = 7.4 Hz, 1H), 7.01 (d, J = 9.1 Hz, 1H), 4.66 (s, 2H), 3.85 – 3.78 (m, 2H), 3.70 (t, J = 5.4 Hz, 4H), 3.64 (dd, J = 6.9, 3.8 Hz, 2H); ^{13}C NMR (101 MHz, DMSO-d6) δ 166.28, 165.50, 163.85, 160.42, 141.55, 137.29, 134.88, 131.92, 129.44, 128.40, 122.90, 121.09, 120.71, 115.46, 112.88, 106.95, 45.28, 44.38, 44.21, 41.76, 37.18; HRMS (ESI) m/z [M+H]$^+$calcd for $C_{25}H_{21}F_3N_7O_2S$: 540.1430, found: 540.1435.

1-(4-(3,4-dichlorophenyl)piperazin-1-yl)-2-((5-(1-(3,4,5-trimethoxyphenyl)-9*H*-pyrido[3,4-b]indol-3-yl)-1,3,4-oxadiazol-2-yl)thio)ethan-1-one (8a):

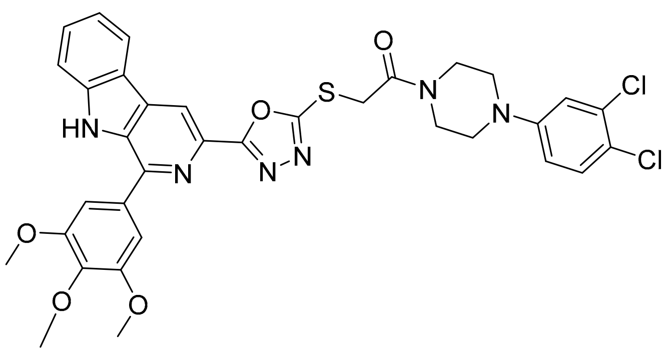

White solid, m.p.147.1−149.2 °C, yield 74%; ^{1}H NMR (400 MHz, DMSO-d_6) δ 11.94 (s, 1H), 8.95 (s, 1H), 8.46 (d, *J* = 7.9 Hz, 1H), 7.69 (d, *J* = 8.2 Hz, 1H), 7.65 – 7.59 (m, 1H), 7.41 (d, *J* = 9.0 Hz, 1H), 7.34 (t, *J* = 7.4 Hz, 1H), 7.24 (s, 2H), 7.17 (d, *J* = 2.9 Hz, 1H), 6.95 (dd, *J* = 9.0, 2.9 Hz, 1H), 4.65 (s, 2H), 3.93 (s, 6H), 3.79 (s, 3H), 3.69 (t, *J* = 5.3 Hz, 2H), 3.63 (t, *J* = 5.4 Hz, 2H), 3.31 (s, 2H), 3.22 (d, *J* = 6.5 Hz, 2H); ^{13}C NMR (101 MHz, DMSO-d_6) δ 166.25, 165.34, 163.92, 153.62, 150.78, 143.66, 142.01, 138.76, 134.51, 133.19, 132.02, 131.98, 131.00, 129.82, 129.37, 122.71, 121.40, 120.87, 120.43, 117.04, 116.02, 114.58, 113.25, 106.51, 60.57, 56.45, 48.04, 47.70, 45.34, 41.73, 37.07; HRMS (ESI) m/z $[M+Na]^+$calcd for $C_{34}H_{30}Cl_2N_6O_5SNa$: 727.1273, found: 727.1265.

1-(4-(4-bromophenyl)piperazin-1-yl)-2-((5-(1-(3,4,5-trimethoxyphenyl)-9*H*-pyrido[3,4-b]indol-3-yl)-1,3,4-oxadiazol-2-yl)thio)ethan-1-one (8b):

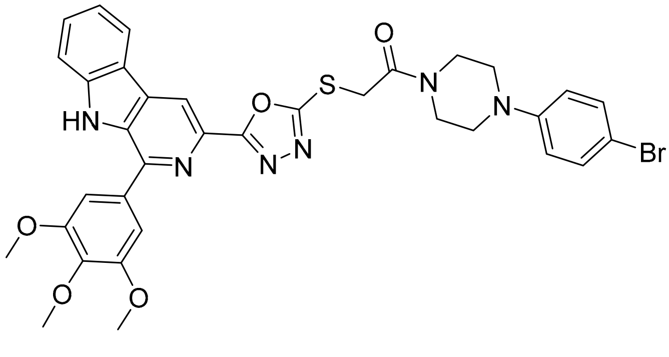

Yellow solid, m.p.155.8−157.9 °C, yield 81%; ^{1}H NMR (400 MHz, DMSO-d_6) δ 12.00 (s, 1H), 8.95 (s, 1H), 8.46 (d, *J* = 7.9 Hz, 1H), 7.74 – 7.70 (m, 1H), 7.64 – 7.59 (m, 1H), 7.38 – 7.30 (m, 3H), 7.24 (s, 2H), 6.94 – 6.88 (m, 2H), 4.65 (s, 2H), 3.94 (s, 6H), 3.79 (s, 3H), 3.70 (t, *J* = 5.2 Hz, 2H), 3.64 (t, *J* = 5.1 Hz, 2H), 3.25 (t, *J* = 5.3 Hz, 2H), 3.17 – 3.12 (m, 2H); ^{13}C NMR (101 MHz, DMSO-d_6) δ 166.25, 165.28, 163.92, 153.61, 150.25, 143.63, 142.04, 138.75, 134.49, 133.19, 132.03, 131.96, 129.83, 129.34, 122.69, 121.40, 120.86, 118.17, 114.57, 113.31, 110.94, 106.51, 60.57, 56.45, 48.52, 48.16, 45.50, 41.87, 37.08; HRMS (ESI) m/z $[M+H]^+$calcd for $C_{34}H_{32}BrN_6O_5S$: 715.1338, found: 715.1331.

1-(4-(4-fluorophenyl)piperazin-1-yl)-2-((5-(1-(3,4,5-trimethoxyphenyl)-9H-pyrido[3,4-b]indol-3-yl)-1,3,4-oxadiazol-2-yl)thio)ethan-1-one (8d):

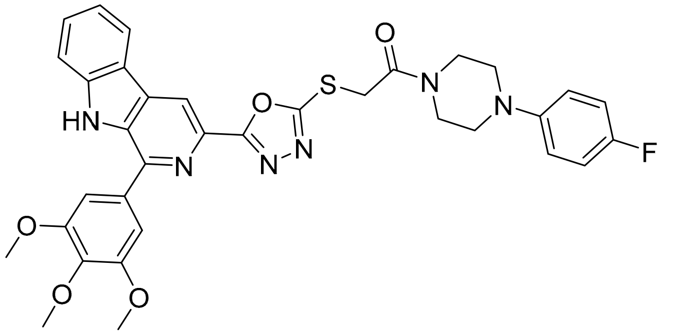

White solid, m.p.156.8−158.1 °C, yield 85%; ^{1}H NMR (400 MHz, DMSO-d_6) δ 11.97 (s, 1H), 8.92 (d, *J* = 1.3 Hz, 1H), 8.42 (d, *J* = 7.9 Hz, 1H), 7.72 – 7.67 (m, 1H), 7.58 (t, *J* = 7.7 Hz, 1H), 7.39 (s, 2H), 7.29 (t, *J* = 7.5 Hz, 1H), 7.06 (tdd, *J* = 9.4, 3.5, 2.2 Hz, 2H), 6.97 (ddd, *J* = 9.2, 4.6, 1.9 Hz, 2H), 4.64 (d, *J* = 1.4 Hz, 2H), 3.93 (s, 6H), 3.79 (s, 3H), 3.72 – 3.61 (m, 4H), 3.17 (t, *J* = 5.0 Hz, 2H), 3.07 (d, *J* = 5.7 Hz, 2H); ^{13}C NMR (101 MHz, DMSO-d_6) δ 165.25, 155.61, 153.56, 148.07, 143.56, 138.69, 129.88, 118.28, 118.20, 115.95, 115.73, 114.54, 106.58, 60.57, 56.45, 49.80, 49.39, 45.75, 42.09, 37.08.; HRMS (ESI) m/z $[M+H]^+$calcd for $C_{34}H_{32}FN_6O_5S$: 655.2139, found: 655.2132.

1-(4-(4-chlorophenyl)piperazin-1-yl)-2-((5-(1-(3,4,5-trimethoxyphenyl)-9*H*-pyrido[3,4-b]indol-3-yl)-1,3,4-oxadiazol-2-yl)thio)ethan-1-one (8e):

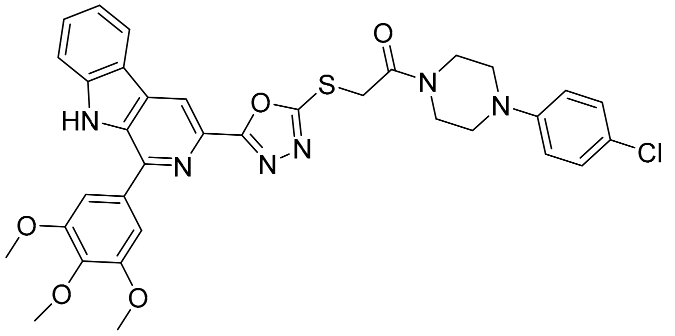

Light yellow solid, m.p.156.2−157.8 °C, yield 76%; ^{1}H NMR (400 MHz, DMSO-d_6) δ 11.94 (s, 1H), 8.95 (s, 1H), 8.46 (d, *J* = 7.8 Hz, 1H), 7.69 (dt, *J* = 8.3, 1.0 Hz, 1H), 7.62 (ddd, *J* = 8.3, 7.0, 1.2 Hz, 1H), 7.34 (ddd, *J* = 8.0, 6.9, 1.1 Hz, 1H), 7.27 – 7.22 (m, 4H), 6.99 – 6.94 (m, 2H), 4.65 (s, 2H), 3.93 (s, 6H), 3.79 (s, 3H), 3.70 (t, *J* = 5.1 Hz, 2H), 3.64 (t, *J* = 5.3 Hz, 2H), 3.25 (t, *J* = 5.2 Hz, 2H), 3.14 (t, *J* = 5.3 Hz, 2H); ^{13}C NMR (101 MHz, DMSO-d_6) δ 166.25, 165.27, 163.93, 153.62, 149.92, 143.65, 142.02,

138.75, 134.51, 133.19, 131.98, 129.83, 129.36, 129.17, 123.29, 122.71, 121.41, 120.87, 117.74, 114.58, 113.25, 106.51, 60.57, 56.45, 48.68, 48.32, 45.53, 41.90, 37.08; HRMS (ESI) m/z $[M+H]^+$calcd for $C_{34}H_{32}ClN_6O_5S$: 671.1843, found: 671.1838.

1-(4-(4-methoxyphenyl)piperazin-1-yl)-2-((5-(1-(3,4,5-trimethoxyphenyl)-9*H*-pyrido[3,4-b]indol-3-yl)-1,3,4-oxadiazol-2-yl)thio)ethan-1-one (8f):

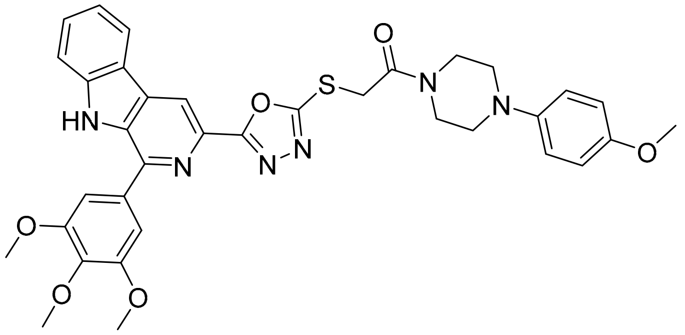

Yellow solid, m.p.156.5−158.6 °C, yield 34%; ^{1}H NMR (400 MHz, DMSO-d_6) δ 11.96 (s, 1H), 8.96 (s, 1H), 8.46 (d, J = 8.0 Hz, 1H), 7.70 (dd, J = 9.3, 2.0 Hz, 1H), 7.62 (ddt, J = 10.0, 7.7, 2.2 Hz, 1H), 7.40 – 7.31 (m, 2H), 7.24 (s, 1H), 6.93 – 6.88 (m, 2H), 6.85 – 6.80 (m, 2H), 4.65 (s, 2H), 3.93 (s, 6H), 3.79 (d, J = 3.1 Hz, 3H), 3.69 (s, 3H), 3.67 – 3.49 (m, 4H), 3.09 (t, J = 5.1 Hz, 2H), 2.99 (t, J = 5.2 Hz, 2H); ^{13}C NMR (101 MHz, DMSO-d_6) δ 166.25, 165.18, 163.95, 153.82, 153.62, 145.50, 143.65, 142.02, 138.76, 134.51, 133.19, 131.99, 129.84, 129.36, 122.72, 121.41, 120.87, 118.53, 114.76, 114.58, 113.25, 106.51, 60.57, 56.45, 55.64, 50.52, 50.11, 45.91, 42.23, 37.11; HRMS (ESI) m/z $[M+H]^+$calcd for $C_{35}H_{35}N_6O_6S$: 667.2339, found: 667.2334.

1-(4-(pyrimidin-2-yl)piperazin-1-yl)-2-((5-(1-(3,4,5-trimethoxyphenyl)-9*H*-pyrido[3,4-b]indol-3-yl)-1,3,4-oxadiazol-2-yl)thio)ethan-1-one (8g):

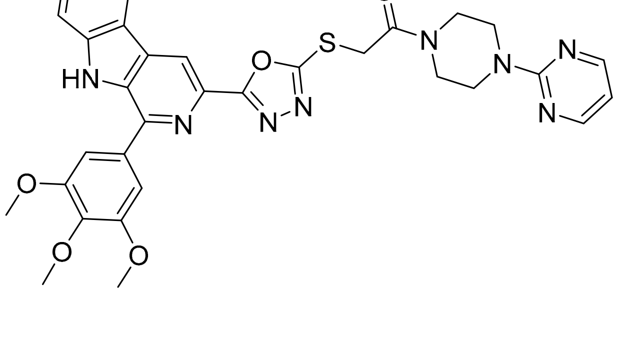

White solid, m.p.249.7−250.7 °C, yield 58%; ^{1}H NMR (400 MHz, DMSO-d_6) δ 11.94 (s, 1H), 8.96 (s, 1H), 8.46 (d, J = 7.9 Hz, 1H), 8.40 (d, J = 4.8 Hz, 2H), 7.69 (d, J = 8.2 Hz, 1H), 7.62 (t, J = 7.6 Hz, 1H), 7.34 (t, J = 7.5 Hz, 1H), 7.24 (s, 2H), 6.68 (t, J = 4.7 Hz, 1H), 4.67 (s, 2H), 3.93 (s, 6H), 3.86 (t, J = 5.1 Hz, 2H), 3.79 (s, 3H), 3.75 (d, J = 5.6 Hz, 2H), 3.65 (t, J = 5.1 Hz, 2H), 3.59 (t, J = 5.3 Hz, 2H); ^{13}C NMR (101 MHz, DMSO-d_6) δ 166.24, 165.41, 163.96, 161.53, 158.48, 153.61, 143.66, 142.01, 138.76, 134.51, 133.19, 131.98, 129.82, 129.36, 122.71, 121.41, 120.86, 114.57, 113.25, 110.98, 106.51, 60.56, 56.45, 45.57, 43.65, 43.40, 41.98, 37.34, 31.16; HRMS (ESI) m/z [M+H]$^+$calcd for $C_{32}H_{31}N_8O_5S$: 639.2138, found: 639.2141.

1-(4-(2,4-difluorophenyl)piperazin-1-yl)-2-((5-(1-(3,4,5-trimethoxyphenyl)-9*H*-pyrido[3,4-b]indol-3-yl)-1,3,4-oxadiazol-2-yl)thio)ethan-1-one (8h):

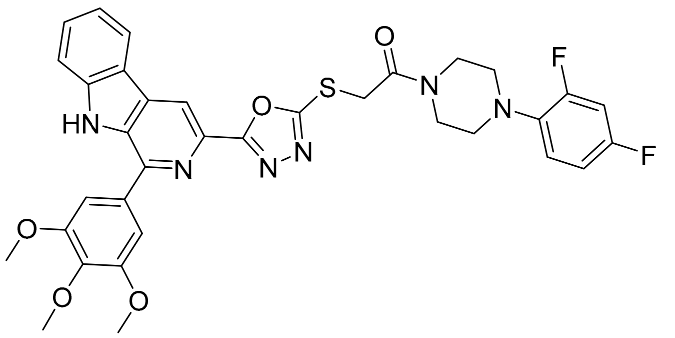

Light brown solid, m.p.178.1−179.4 °C, yield 85%; ^{1}H NMR (400 MHz, DMSO-d_6) δ 12.00 (s, 1H), 8.96 (s, 1H), 8.46 (d, J = 7.9 Hz, 1H), 7.72 (d, J = 8.2 Hz, 1H), 7.62 (ddd, J = 8.3, 7.0, 1.2 Hz, 1H), 7.34 (ddd, J = 8.0, 7.0, 1.0 Hz, 1H), 7.26 – 7.19 (m, 3H), 7.10 – 6.97 (m, 2H), 4.65 (s, 2H), 3.94 (s, 6H), 3.79 (s, 3H), 3.71 (t, J = 5.0 Hz, 2H), 3.68 – 3.63 (m, 2H), 3.04 (t, J = 5.1 Hz, 2H), 2.95 (t, J = 5.0 Hz, 2H); ^{13}C NMR (101 MHz, DMSO-d_6) δ 166.26, 165.29, 163.92, 153.61, 143.64, 142.04, 138.75, 134.49, 133.19, 131.96, 129.83, 129.35, 122.70, 121.40, 121.00, 120.87, 114.59, 113.32, 111.46, 106.51, 105.19, 104.93, 60.56, 56.45, 51.03, 50.68, 46.01, 42.31, 37.16; HRMS (ESI) m/z [M+H]$^+$calcd for $C_{34}H_{31}F_2N_6O_5S$: 673.2045, found: 673.2038.

1-(4-phenylpiperazin-1-yl)-2-((5-(1-(3,4,5-trimethoxyphenyl)-9*H*-pyrido[3,4-b]indol-3-yl)-1,3,4-oxadiazol-2-yl)thio)ethan-1-one (8i):

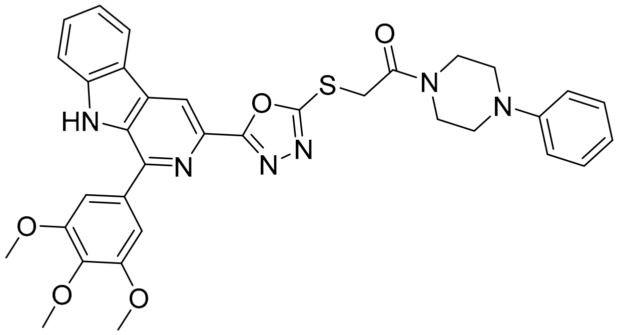

White solid, m.p.219.5−220.7 °C, yield 33%; ^{1}H NMR (400 MHz, DMSO-d_6) δ 11.99 (d, J = 4.2 Hz, 1H), 8.97 – 8.93 (m, 1H), 8.46 (d, J = 8.0 Hz, 1H), 7.71 (d, J = 8.3 Hz, 1H), 7.62 (ddd, J = 8.2, 6.9, 1.2 Hz, 1H), 7.34 (t, J = 7.5 Hz, 1H), 7.26 – 7.21 (m, 4H), 6.96 (d, J = 8.2 Hz, 2H), 6.82 (t, J = 7.3 Hz, 1H), 4.66 (s, 2H), 3.94 (s, 6H), 3.79 (s, 3H), 3.71 (t, J = 4.9 Hz, 2H), 3.67 – 3.62 (m, 2H), 3.24 (t, J = 5.1 Hz, 2H), 3.14 (t, J = 5.3 Hz, 2H); ^{13}C NMR (101 MHz, DMSO-d_6) δ 166.25, 165.24, 163.94, 153.61, 151.14, 143.64, 142.06, 138.75, 133.20, 131.95, 129.83, 129.48, 129.34, 122.70, 121.41, 120.85, 119.83, 116.35, 114.57, 113.30, 106.52, 60.57, 56.45, 48.95, 48.59, 45.71, 42.06, 37.11; HRMS (ESI) m/z [M+H]$^{+}$calcd for $C_{34}H_{33}N_6O_5S$: 637.2233, found: 637.2228.

1-(4-(3-methoxyphenyl)piperazin-1-yl)-2-((5-(1-(3,4,5-trimethoxyphenyl)-9*H*-pyrido[3,4-b]indol-3-yl)-1,3,4-oxadiazol-2-yl)thio)ethan-1-one (8j):

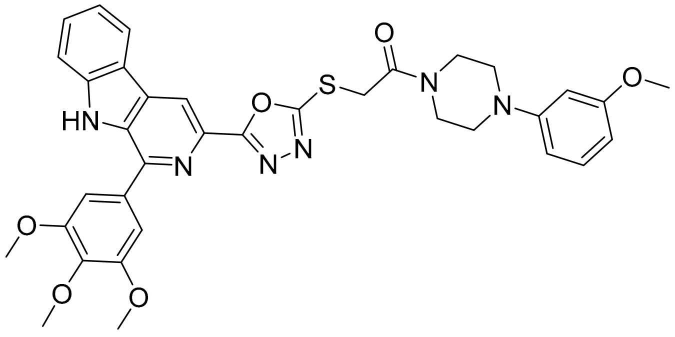

Yellow solid, m.p.140.4−141.6 °C, yield 44%; ^{1}H NMR (400 MHz, DMSO-d_6) δ 11.98 (s, 1H), 8.96 (d, J = 2.2 Hz, 1H), 8.46 (d, J = 7.9 Hz, 1H), 7.71 (d, J = 8.2 Hz, 1H), 7.62 (ddd, J = 8.2, 6.9, 1.2 Hz, 1H), 7.34 (td, J = 7.5, 7.0, 1.0 Hz, 1H), 7.24 (s, 2H), 7.13 (t, J = 8.2 Hz, 1H), 6.54 (dd, J = 8.3, 2.3 Hz, 1H), 6.48 (q, J = 2.2 Hz, 1H), 6.40 (dd, J = 8.1, 2.3 Hz, 1H), 4.66 (s, 2H), 3.93 (s, 6H), 3.79 (s, 3H), 3.72 (s, 3H), 3.71 – 3.65 (m, 2H), 3.64 (t, J = 5.2 Hz, 2H), 3.24 (t, J = 5.2 Hz, 2H), 3.15 (d, J = 5.3 Hz, 2H); ^{13}C NMR (101 MHz, DMSO-d_6) δ 166.25, 165.23, 163.95, 160.69, 153.61, 152.49, 143.64, 142.03, 138.75, 134.49, 133.19, 131.97, 130.18, 129.83, 129.35, 122.70, 121.40, 120.87, 114.57, 113.30, 108.89,

106.51, 105.11, 102.47, 60.57, 56.45, 55.38, 48.88, 48.54, 45.65, 42.03, 37.12; HRMS (ESI) m/z [M+H]$^+$calcd for $C_{35}H_{35}N_6O_6S$: 667.2339, found: 667.2338.

N-(4-fluorophenyl)-2-((5-(1-(3,4,5-trimethoxyphenyl)-9*H*-pyrido[3,4-b]indol-3-yl)-1,3,4-oxadiazol-2-yl)thio)acetamide (8k):

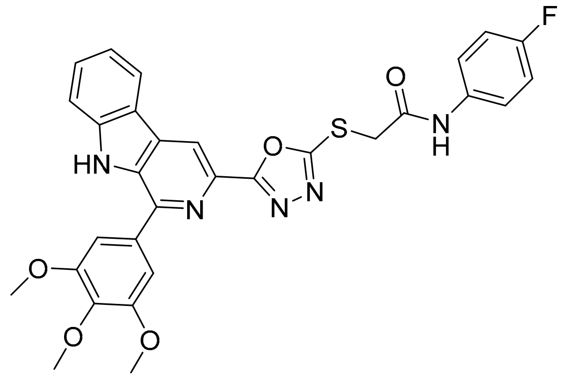

Yellow solid, m.p.235.8–237.4 °C, yield 74%; ^{1}H NMR (400 MHz, DMSO-d_6) δ 11.97 (s, 1H), 10.65 (d, J = 3.2 Hz, 1H), 8.93 (s, 1H), 8.42 (d, J = 7.9 Hz, 1H), 7.70 (d, J = 8.2 Hz, 1H), 7.66 – 7.59 (m, 3H), 7.34 (ddd, J = 8.1, 7.0, 1.1 Hz, 1H), 7.23 (s, 2H), 7.19 – 7.10 (m, 2H), 4.40 (s, 2H), 3.93 (s, 6H), 3.80 (s, 3H); ^{13}C NMR (101 MHz, DMSO-d_6) δ 166.37, 165.35, 163.69, 157.47, 153.61, 143.69, 142.01, 138.74, 135.57, 134.50, 133.18, 131.92, 129.80, 129.36, 122.66, 121.55, 121.47, 121.38, 120.86, 116.01, 115.79, 114.58, 113.28, 106.50, 60.56, 56.44, 37.28; HRMS (ESI) m/z [M+H]$^+$calcd for $C_{30}H_{25}FN_5O_5S$: 586.1560, found: 586.1556.

1-(4-(methylsulfonyl)piperazin-1-yl)-2-((5-(1-(3,4,5-trimethoxyphenyl)-9*H*-pyrido[3,4-b]indol-3-yl)-1,3,4-oxadiazol-2-yl)thio)ethan-1-one (8m):

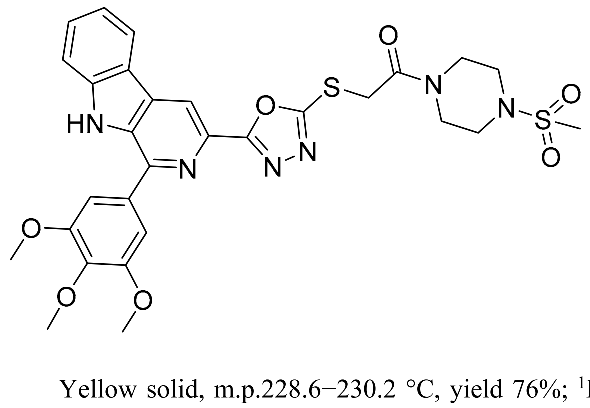

Yellow solid, m.p.228.6–230.2 °C, yield 76%; ^{1}H NMR (400 MHz, DMSO-d_6) δ 12.00 (s, 1H), 8.96 (d, J = 1.4 Hz, 1H), 8.47 (d, J = 7.9 Hz, 1H), 7.72 (d, J = 8.3 Hz, 1H), 7.62 (ddd, J = 8.3, 7.0, 1.2

Hz, 1H), 7.35 (ddd, J = 8.0, 7.0, 1.0 Hz, 1H), 7.24 (s, 2H), 4.64 (s, 2H), 3.94 (s, 6H), 3.80 (s, 3H), 3.67 (t, J = 5.1 Hz, 2H), 3.61 (t, J = 5.2 Hz, 2H), 3.23 (t, J = 5.0 Hz, 2H), 3.13 (t, J = 5.2 Hz, 2H), 2.91 (s, 3H); ^{13}C NMR (101 MHz, DMSO-d_6) δ 166.28, 165.46, 163.86, 153.62, 143.65, 142.04, 138.76, 134.50, 133.19, 131.95, 129.84, 129.36, 122.73, 121.40, 120.88, 114.60, 113.32, 106.52, 60.58, 56.47, 45.76, 45.50, 41.80, 37.26, 34.78; HRMS (ESI) m/z [M+H]$^+$calcd for $C_{29}H_{31}N_6O_7S_2$: 639.1696, found: 639.1700.

1-(2,3-dihydro-4*H*-benzo[b][1,4]oxazin-4-yl)-2-((5-(1-(3,4,5-trimethoxyphenyl)-9*H*-pyrido[3,4-b]indol-3-yl)-1,3,4-oxadiazol-2-yl)thio)ethan-1-one (8n):

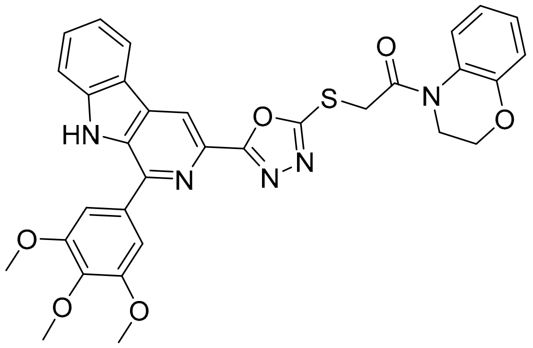

White solid, m.p.225.9–226.8 °C, yield 58%; ^{1}H NMR (400 MHz, DMSO-d_6) δ 11.94 (s, 1H), 8.94 (s, 1H), 8.45 (d, J = 7.9 Hz, 1H), 7.74 – 7.57 (m, 3H), 7.34 (td, J = 7.5, 6.9, 1.1 Hz, 1H), 7.23 (s, 2H), 7.09 (s, 1H), 6.99 – 6.79 (m, 2H), 4.81 (s, 2H), 4.34 (t, J = 4.6 Hz, 2H), 4.00 – 3.95 (m, 2H), 3.92 (s, 6H), 3.80 (s, 3H); ^{13}C NMR (101 MHz, DMSO-d_6) δ 166.31, 163.82, 153.61, 143.68, 142.01, 138.75, 134.52, 133.19, 131.92, 129.80, 129.36, 126.09, 122.70, 121.40, 120.87, 120.38, 117.49, 114.56, 113.25, 106.51, 60.57, 56.44; HRMS (ESI) m/z [M+H]$^+$calcd for $C_{32}H_{28}N_5O_6S$: 610.1760, found: 610.1575.

1-(4-cyclohexylpiperazin-1-yl)-2-((5-(1-(3,4,5-trimethoxyphenyl)-9*H*-pyrido[3,4-b]indol-3-yl)-1,3,4-oxadiazol-2-yl)thio)ethan-1-one (8p):

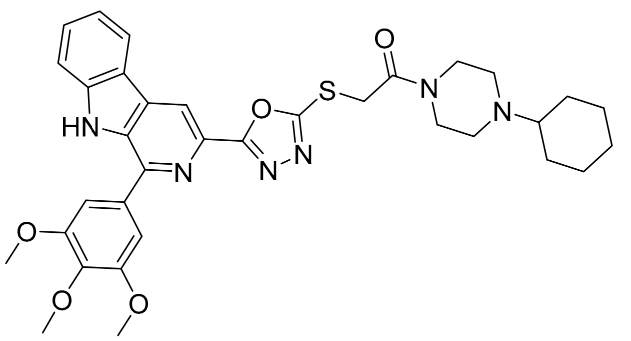

Yellow solid, m.p.125.5−126.8 °C, yield 40%; ^{1}H NMR (400 MHz, DMSO-d_6) δ 11.96 (d, J = 4.3 Hz, 1H), 8.96 (s, 1H), 8.47 (d, J = 7.9 Hz, 1H), 7.71 – 7.67 (m, 1H), 7.62 (ddd, J = 8.3, 7.0, 1.2 Hz, 1H), 7.35 (ddd, J = 8.0, 6.9, 1.1 Hz, 1H), 7.24 (d, J = 2.5 Hz, 2H), 4.58 (s, 2H), 3.93 (s, 6H), 3.80 (s, 3H), 3.50 (t, J = 5.0 Hz, 2H), 3.46 (t, J = 5.0 Hz, 2H), 2.54 (d, J = 4.7 Hz, 2H), 2.45 (t, J = 5.1 Hz, 2H), 2.23 (d, J = 8.9 Hz, 1H), 1.71 (d, J = 10.6 Hz, 4H), 1.55 (d, J = 12.3 Hz, 1H), 1.23 (s, 1H), 1.20 – 1.13 (m, 3H), 1.11 – 1.01 (m, 1H); ^{13}C NMR (101 MHz, DMSO-d_6) δ 166.22, 164.96, 163.96, 153.62, 143.64, 142.02, 138.76, 134.50, 133.19, 132.00, 129.84, 129.37, 122.72, 121.41, 120.87, 114.58, 113.26, 106.50, 63.12, 60.57, 56.45, 49.00, 48.57, 42.71, 37.16, 28.67, 26.28, 25.73; HRMS (ESI) m/z [M+H]$^+$calcd for $C_{34}H_{39}N_6O_5S$: 643.2703, found: 643.2697.

N-(4-(morpholinomethyl)phenyl)-2-((5-(1-(3,4,5-trimethoxyphenyl)-9*H*-pyrido[3,4-b]indol-3-yl)-1,3,4-oxadiazol-2-yl)thio)acetamide (8q):

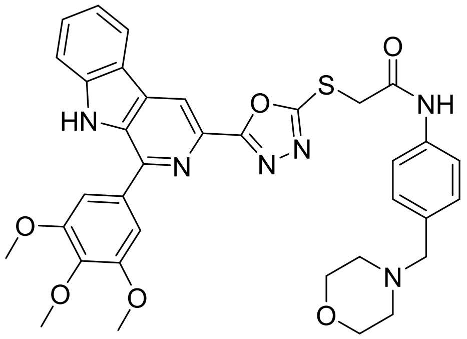

Black solid, m.p.181.8−182.7 °C, yield 72%; ^{1}H NMR (400 MHz, DMSO-d_6) δ 11.97 (s, 1H), 10.64 – 10.55 (m, 1H), 8.94 (s, 1H), 8.42 (d, J = 7.9 Hz, 1H), 7.70 (d, J = 8.2 Hz, 1H), 7.62 (t, J = 7.6 Hz, 1H), 7.56 (dt, J = 8.4, 1.6 Hz, 2H), 7.34 (t, J = 7.5 Hz, 1H), 7.22 (d, J = 5.6 Hz, 4H), 4.41 (d, J = 2.1 Hz, 2H), 3.92 (s, 6H), 3.79 (s, 3H), 3.54 (t, J = 4.6 Hz, 4H), 3.39 (s, 2H), 2.30 (d, J = 4.9 Hz, 4H); ^{13}C NMR (101 MHz, DMSO-d_6) δ 166.34, 165.30, 163.70, 153.60, 143.68, 142.01, 138.74, 134.48, 133.18, 131.92, 129.90, 129.80, 129.34, 122.67, 121.39, 120.85, 119.52, 114.59, 113.29, 106.50, 66.58, 62.39, 60.57, 56.44, 53.51, 37.35; HRMS (ESI) m/z [M+H]$^+$calcd for $C_{35}H_{35}N_6O_6S$: 667.2339, found: 667.2341.

4-(4-(2-((5-(1-(3,4,5-trimethoxyphenyl)-9*H*-pyrido[3,4-b]indol-3-yl)-1,3,4-oxadiazol-2-yl)thio)acetyl)piperazin-1-yl)benzonitrile (8s):

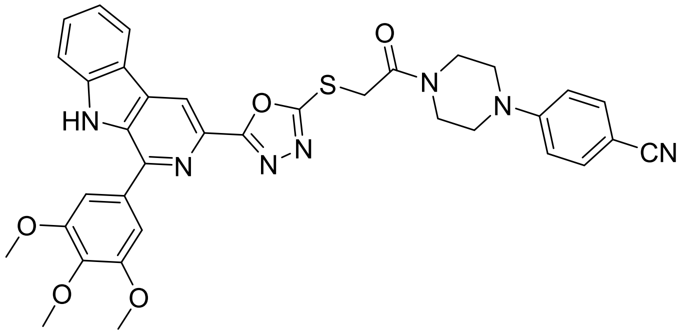

Brown solid, m.p.150.3−151.5 °C, yield 40%; ^{1}H NMR (400 MHz, DMSO-d_6) δ 12.00 (s, 1H), 8.96 (s, 1H), 8.46 (d, J = 7.9 Hz, 1H), 7.72 (d, J = 8.3 Hz, 1H), 7.64 – 7.58 (m, 3H), 7.34 (t, J = 7.5 Hz, 1H), 7.24 (s, 2H), 7.03 (d, J = 8.6 Hz, 2H), 4.66 (s, 2H), 3.93 (s, 6H), 3.79 (s, 3H), 3.72 (t, J = 5.1 Hz, 2H), 3.67 – 3.60 (m, 2H), 3.50 (d, J = 5.2 Hz, 2H), 3.40 (d, J = 5.5 Hz, 2H); ^{13}C NMR (101 MHz, DMSO-d_6) δ 166.26, 165.47, 163.92, 153.62, 153.17, 143.64, 142.04, 138.75, 134.50, 133.86, 133.19, 131.96, 129.84, 121.39, 120.87, 114.56, 113.32, 106.51, 98.95, 60.57, 56.46, 46.56, 46.34, 45.12, 41.63, 37.05; HRMS (ESI) m/z [M+H]$^+$calcd for $C_{35}H_{32}N_7O_5S$: 662.2186, found: 662.2189.

2. Exploration of *in vitro* antitumor mechanisms

2.1. Colony formation assay

PC-3 cells were seeded in six-well plate with 1000 cells per well, incubated for 24 h at 37 ℃ and 5% CO_2. Subsequently, different concentrations of **8q** and positive drug were added to the wells and cultured for 14 days. During this period, serum RPMI-1640 medium was supplemented. After 14 days, the culture medium was removed, fixed with 4% paraformaldehyde fixative for 20 min, washed twice with PBS, and finally stained with 0.1% crystal violet. The formation of cell colonies was observed by taking photos.

2.2.Wound healing assay

To evaluate the effect of compound **8q** on the anti-metastatic ability of PC-3 cells,

a wound healing assay was performed using the method we previously reported[1]. In short, PC-3 cells were seeded in a six-well plate at a density of 10 × 10^5 and incubated at a constant temperature of 37 °C and 5% CO_2 for 24 h. Then the pipette tip was used to create a uniform wound on the surface of cells. Subsequently, after cleaning twice with PBS, the scratch healing of the **8q** treatment group and the positive drug treatment group were observed at different concentrations at 0 h, 12 h, and 24 h. Image J software was used to measure and quantify cell migration.

2.3.The effect of compound 8q on cell apoptosis

We used Annexin V-FITC/ PI double staining to evaluate cellular apoptosis via flow cytometry to figure out if apoptosis participates in the inhibition of cell growth. PC-3 cells were seeded in a six-well plate at a density of 3 × 10^5, and cultured at 37 °C and 5% CO_2 for 24 h before adding 10 μM. 20 μM and 30 μM compound **8q** and 30 μM Harmine to each well. After 48 h, the cells were collected and stained and incubated flowing the manufacturer's protocol of the Annexin V-FITC/PI Apoptosis Detection Kit (Solarbio, Beijing, China). After completion, the cells were immediately detected on the Flow Cytometry (Solarbio, Beijing, China). The experimental results were processed using Flow Jo and Prism 7.04 software.

2.4.The effect of compound 8q on cellular reactive oxygen species levels

PC-3 cells were seeded at a density of 3 × 10^5 in a six-well plate and cultured for 24 h under 37 °C and 5% CO_2 conditions. Then, different concentrations of compound **8q** and Harmine were added and cultured for 24 h. The cells were collected and washed once with PBS. Diluted DCFH-DA staining solution (1:1000) was added and incubated

at 37 ° C for 20 min. Subsequently, the staining solution was wash off with basic culture medium and detected the fluorescence intensity using the flow cytometer. The data was analyzed and obtained through Flow Jo and Prism 7.04 software.

2.5. Cell cycle arrest of compound 8q on PC-3

To determine the effect of compound **8q** on the cell cycle distribution of PC-3 cell, we used our previous method with slight modifications[2]. Briefly, cells were incubated in a six-well plate with a density of 3×10^5 at 37 °C and 5% CO_2 for 24 h, followed by the addition of different concentrations of compound **8q** for further incubation for 24 h. Next, the cells were collected and washed twice with PBS. 75% anhydrous ethanol was used to immobilize the cells under overnight conditions at 4 °C. Subsequently, PBS washed cells, and RNase from the cell cycle assay kit (Solarbio, Beijing, China) was added and incubated in a constant temperature (37 °C) water bath for 30 min. Then PI was added under dark conditions and incubated at 4 °C for another half hour to detect. The distribution of cycles was analyzed by Flow Jo and Prism 7.04 software.

2.6.Effects of 8q on expression of cell cycle related protein

PC-3 cells were seeded in six-well plates at a density of 3×10^5 per well and incubated in 5 % CO_2 incubator at 37 °C for 24 h. Then different concentrations of **8q** and Harmine were added to continue cultivation for 24 h. The prepared 1× RIPA lysis buffer (Solarbio, Beijing, China), as well as protease (Solarbio, Beijing, China) and phosphatase inhibitors (Solarbio, Beijing, China), were added to the well in a ratio of 100:1:1. The cells were scraped off and then lysed on ice for 40 min. Mixed liquid was centrifuged, and the supernatant used the BCA protein assay kit (Beyotime, Shanghai,

China) to measure protein concentration. 12.5% SDS-PAGE gel was used to separate the extracted total protein, which was transferred onto a PVDF membrane and sealed with 5% skim milk for 2.5 h. After that, primary antibodies included β-Tubulin and Cyclin D1 were added and incubated overnight at 4 °C. Subsequently, the second antibody (Cell signaling Technology, Type: Mouse and Ribbit; Cat No.: #7076 and #7074) was added and incubated at room temperature for 1 hour. Finally, chemiluminescence was used to assist in observing the bands.

3. Spectra of compounds 7 series and 8 series.

Spectra of compounds (^{1}H NMR, ^{13}C NMR, HRMS)

^{1}H NMR, ^{13}C NMR and HRMS (AP-ESI) spectra of target compounds 7 series and 8 series.

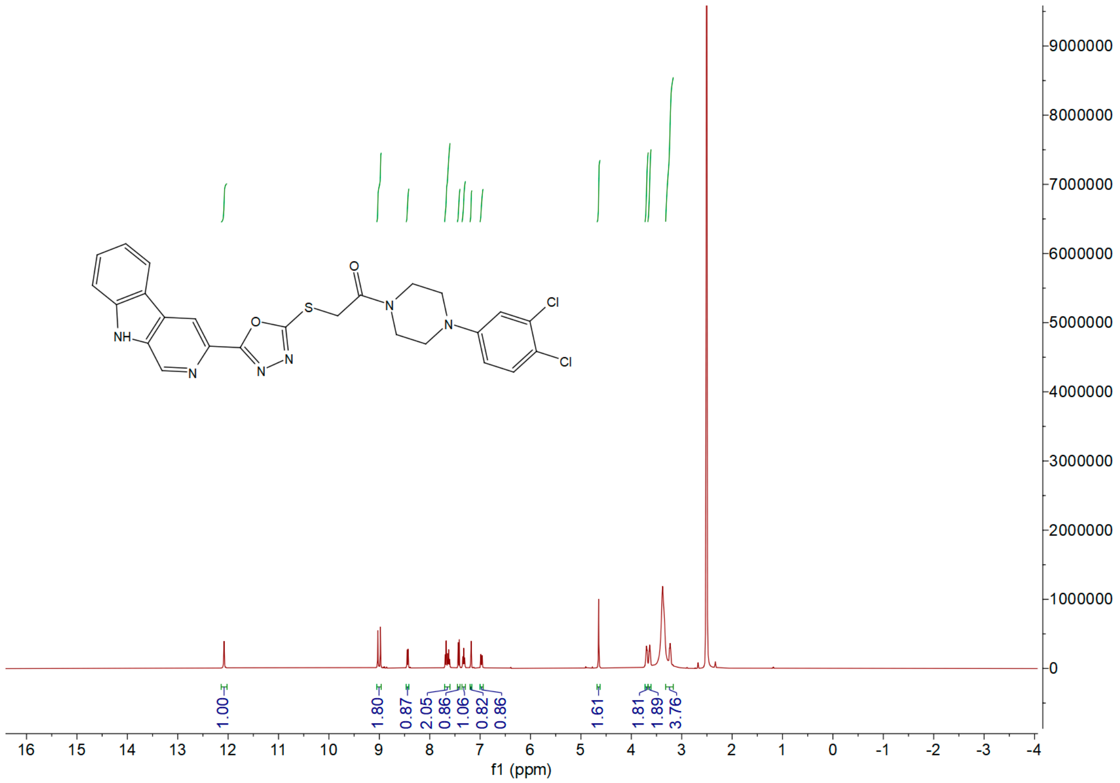

Figure S1 The ^{1}H NMR Spectrum of compound **7a**

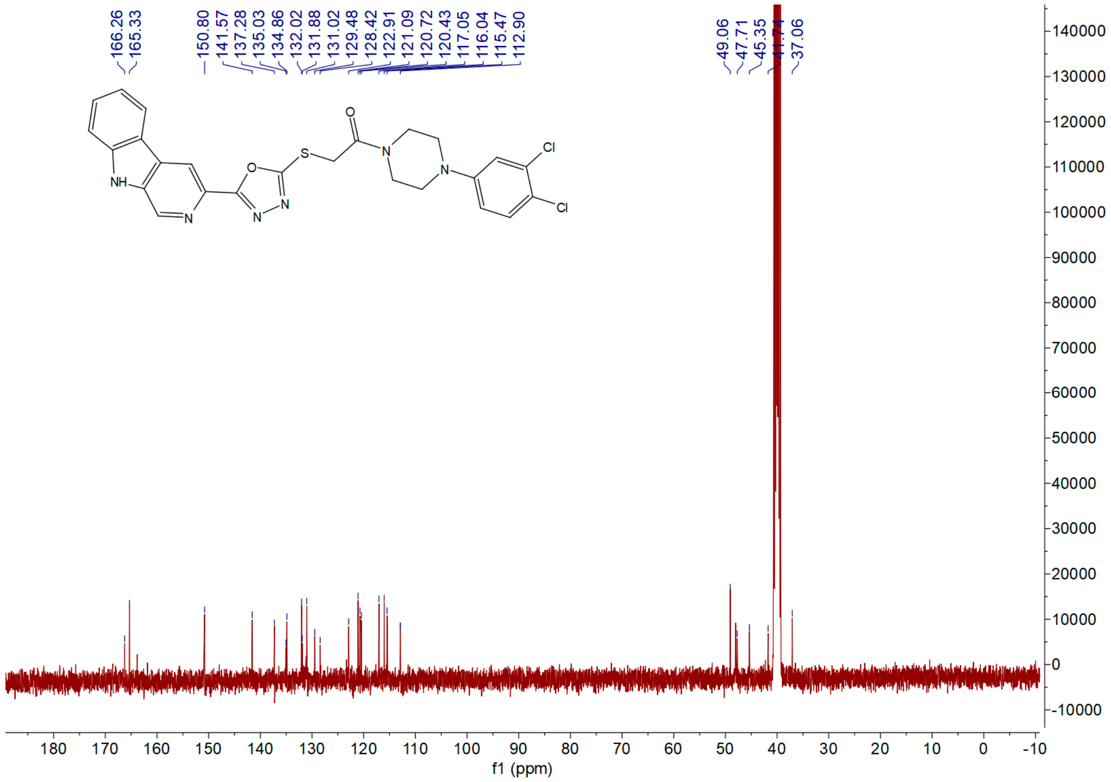

Figure S2 The ^{13}C NMR Spectrum of compound **7a**

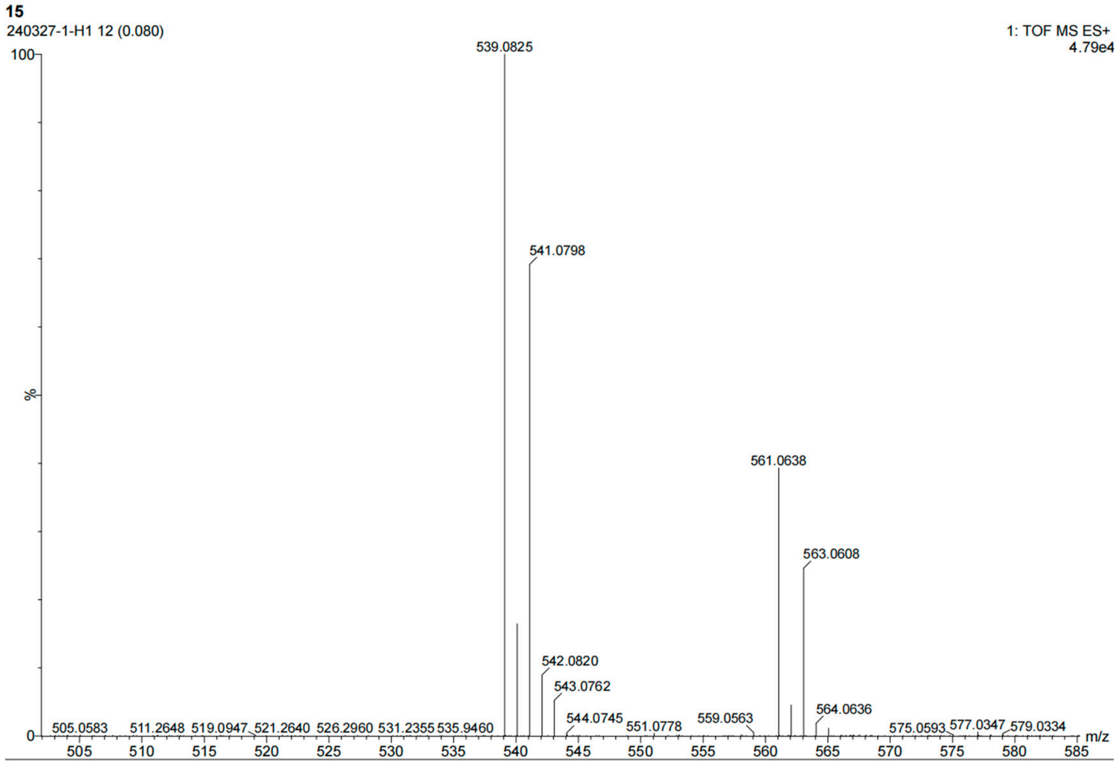

Figure S3 The HRMS spectrum of compound **7a**

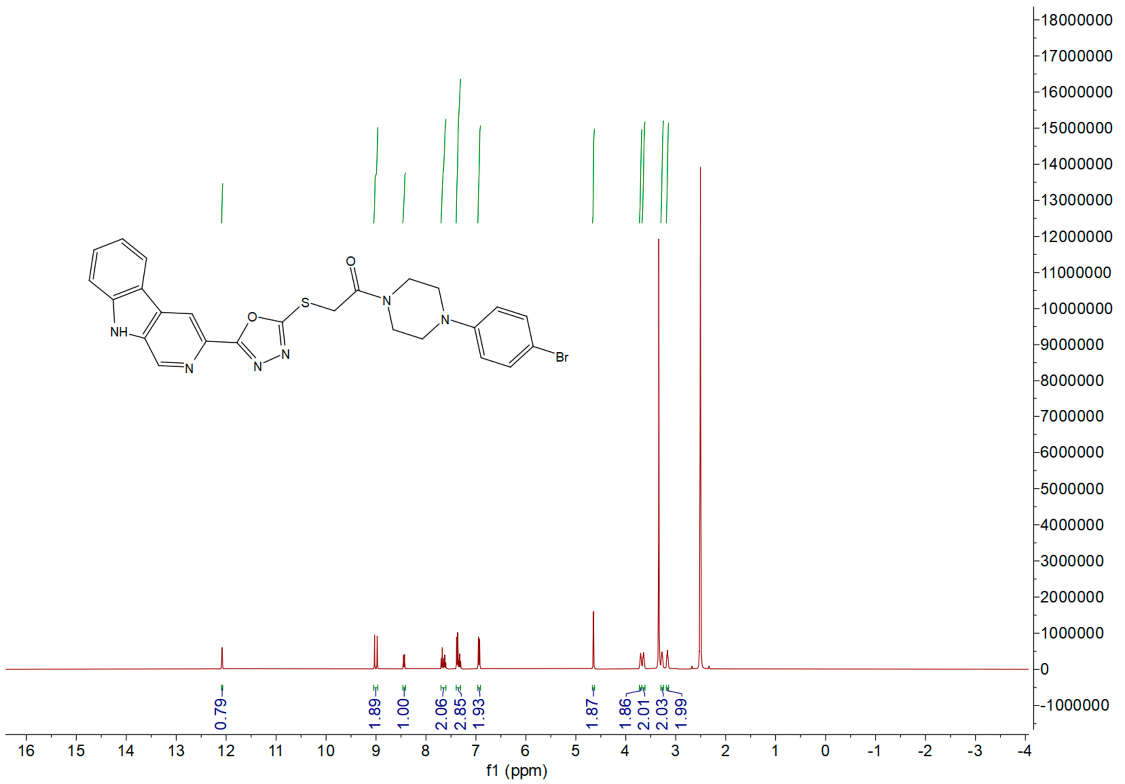

Figure S4 The ^{1}H NMR Spectrum of compound **7b**

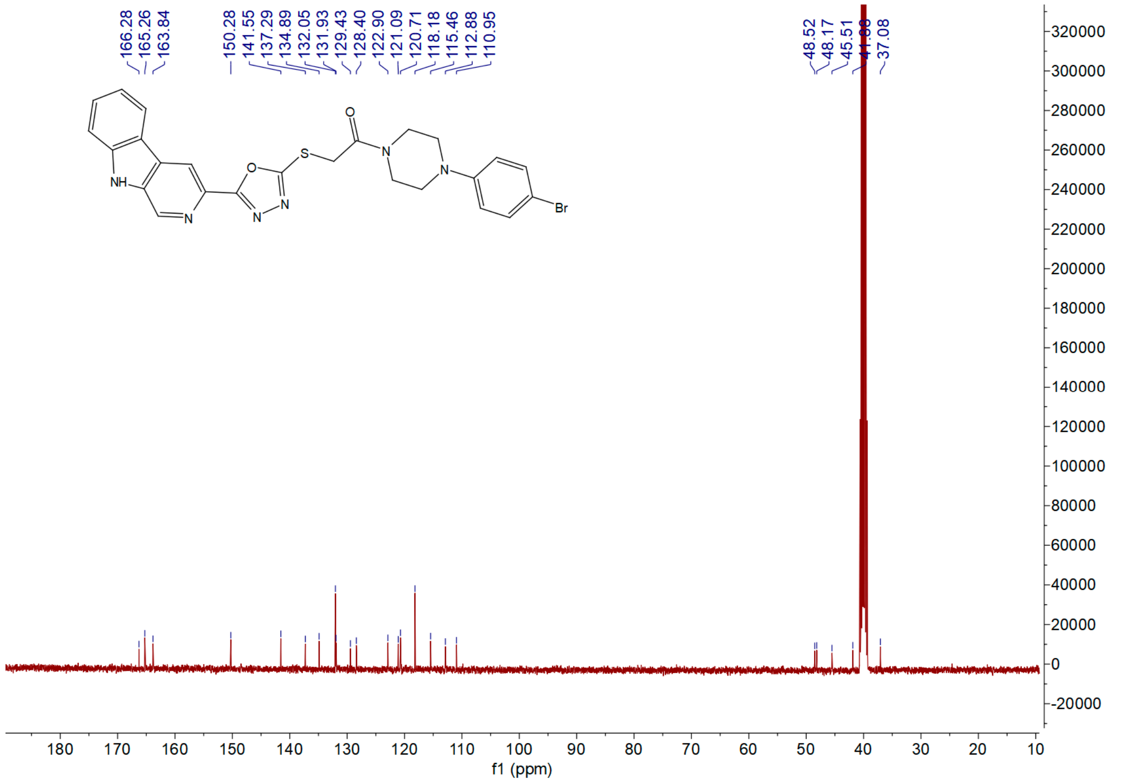

Figure S5 The ^{13}C NMR Spectrum of compound **7b**

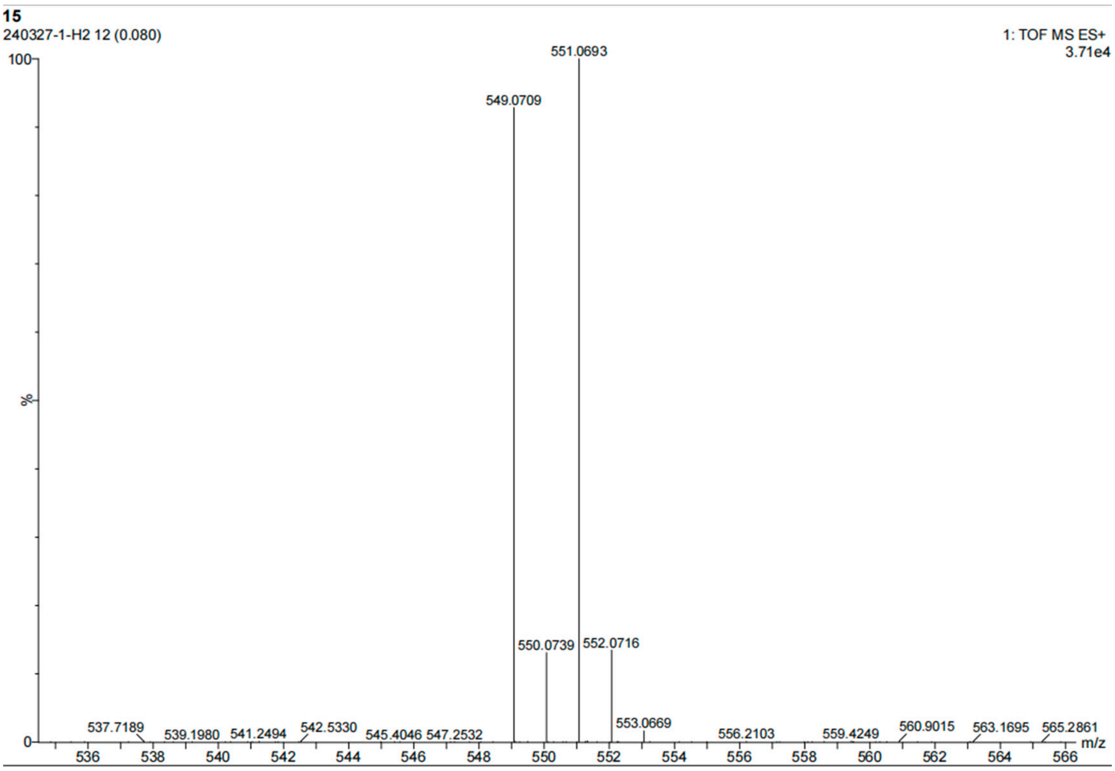

Figure S6 The HRMS spectrum of compound **7b**

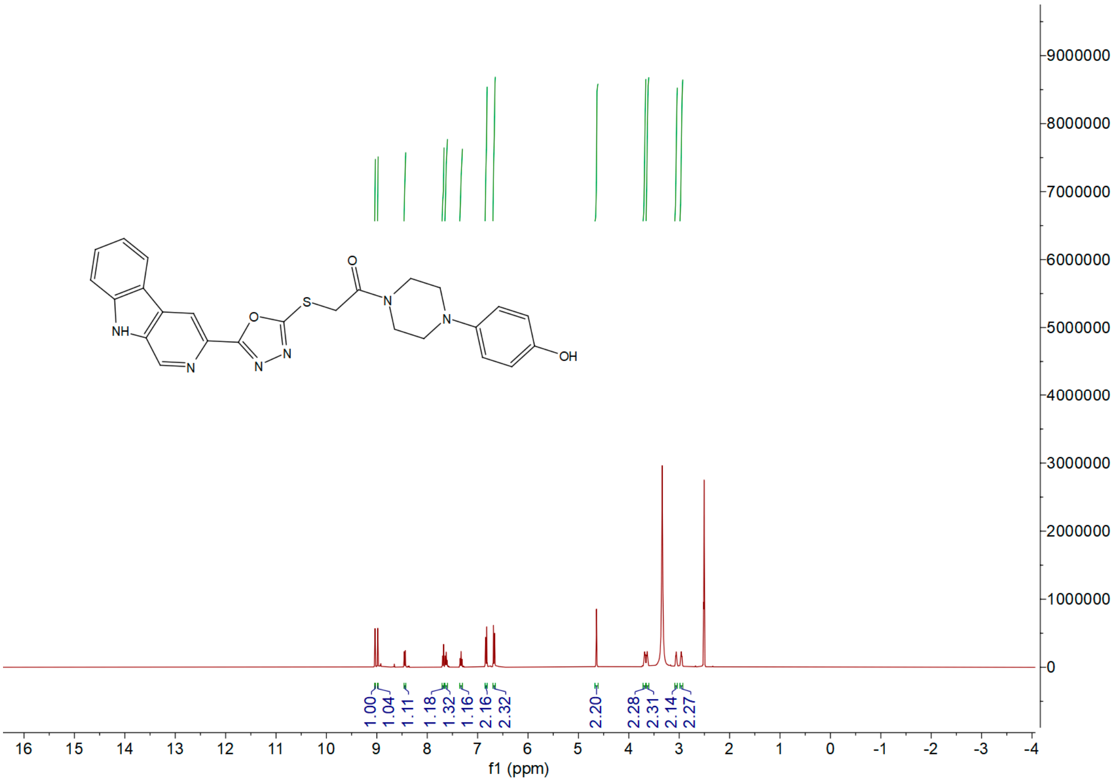

Figure S7 The ^{1}H NMR Spectrum of compound **7c**

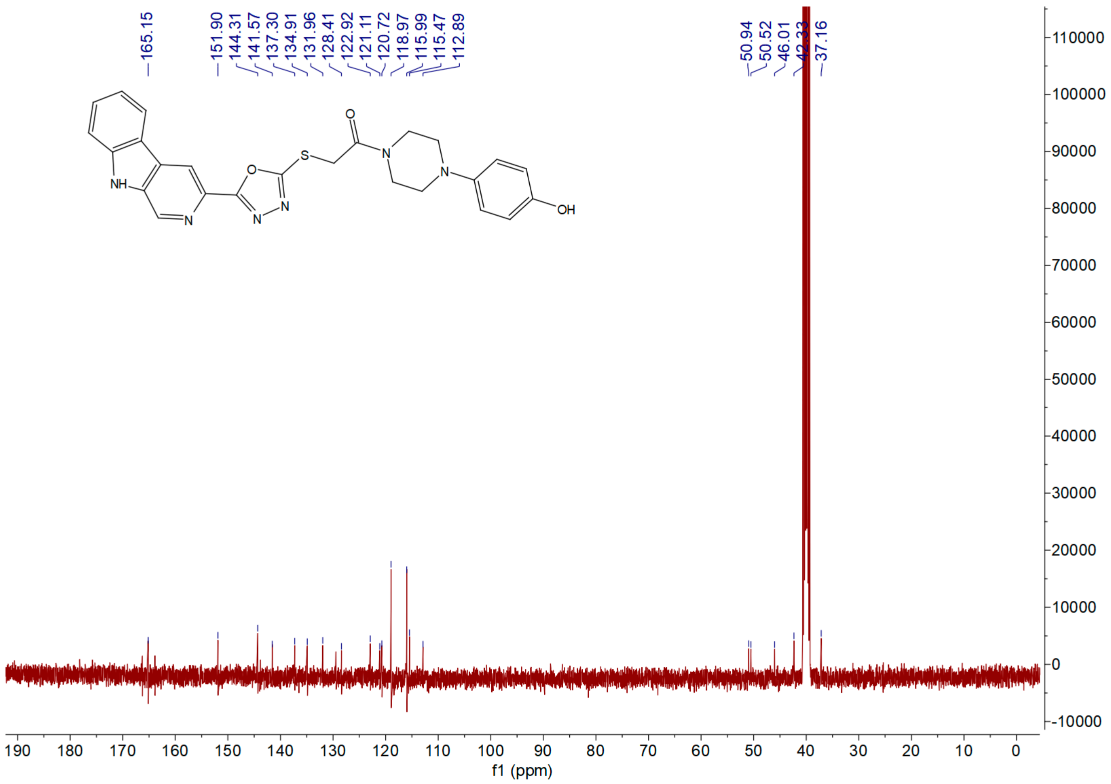

Figure S8 The ^{13}C NMR Spectrum of compound **7c**

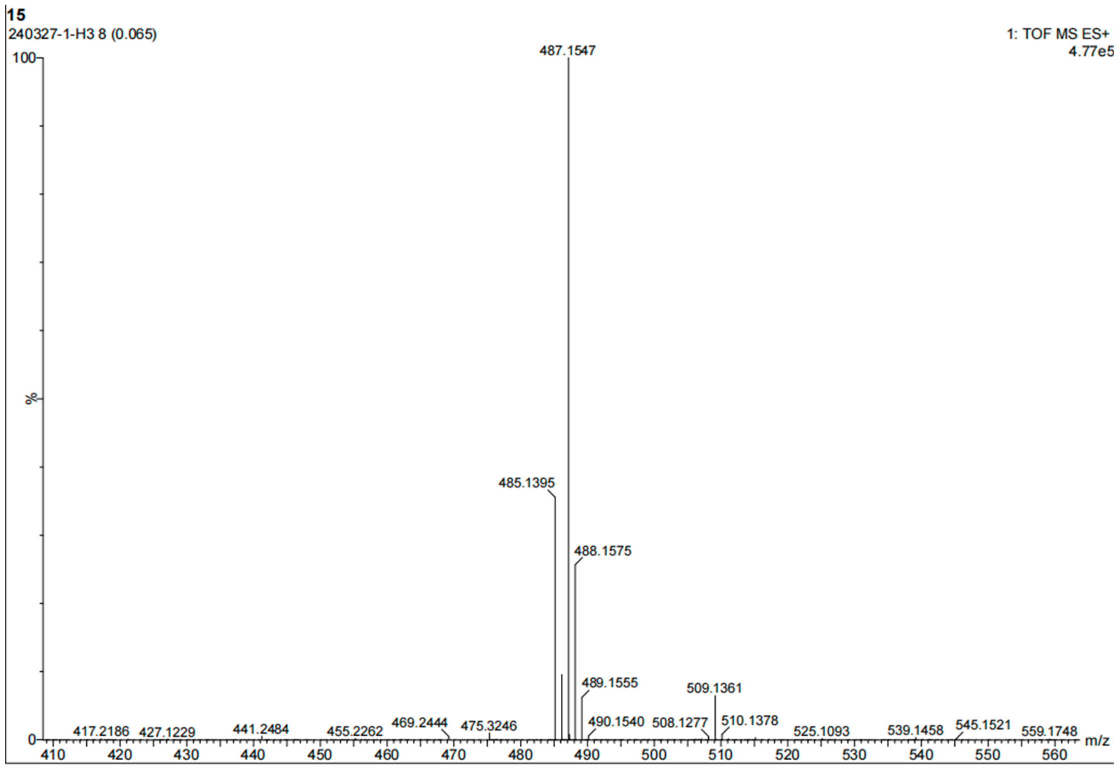

Figure S9 The HRMS spectrum of compound **7c**

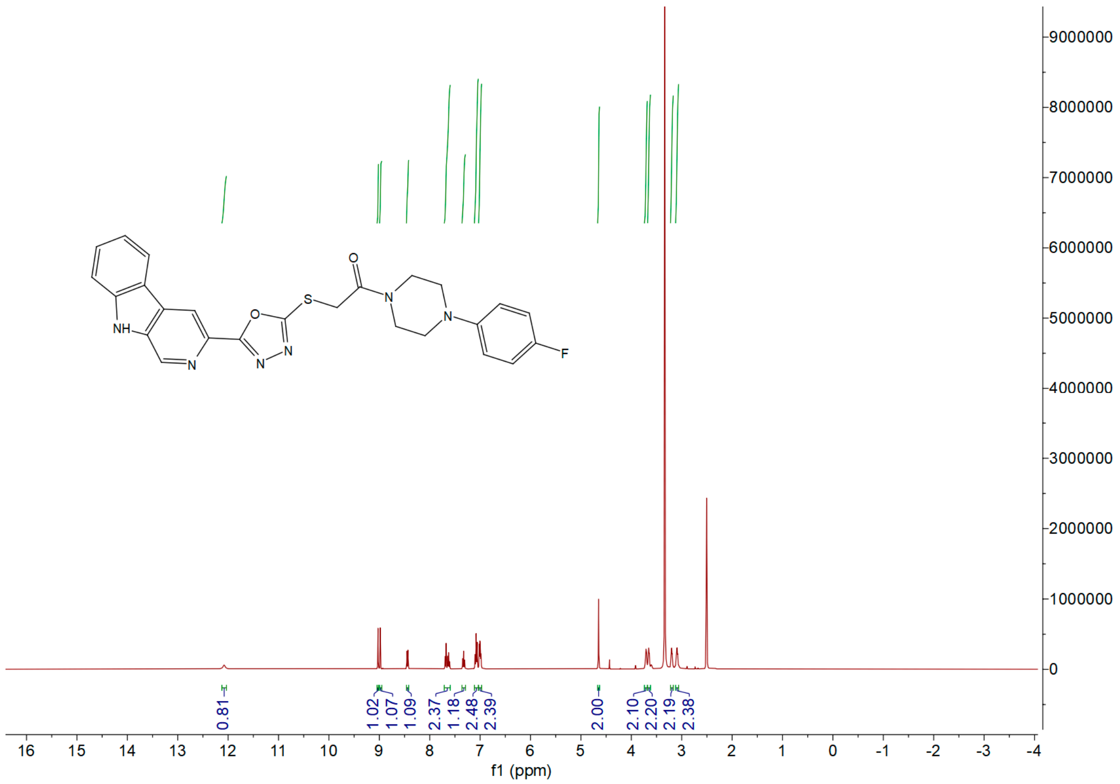

Figure S10 The ^{1}H NMR Spectrum of compound **7d**

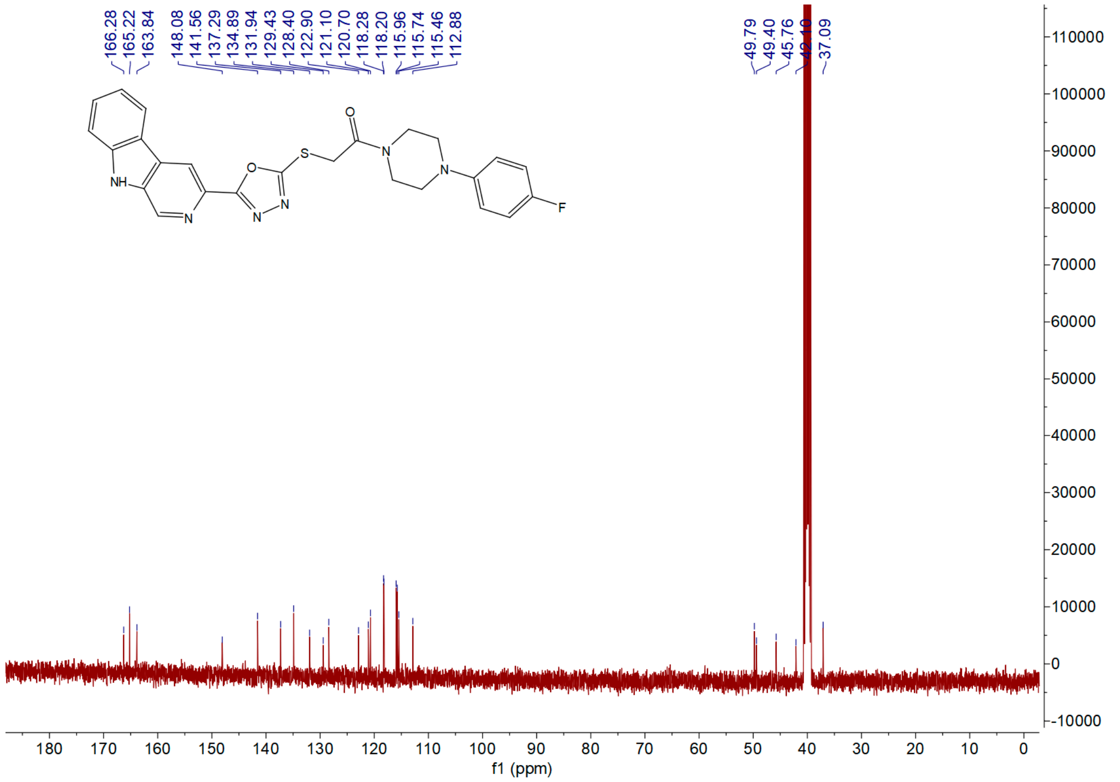

Figure S11 The ^{13}C NMR Spectrum of compound **7d**

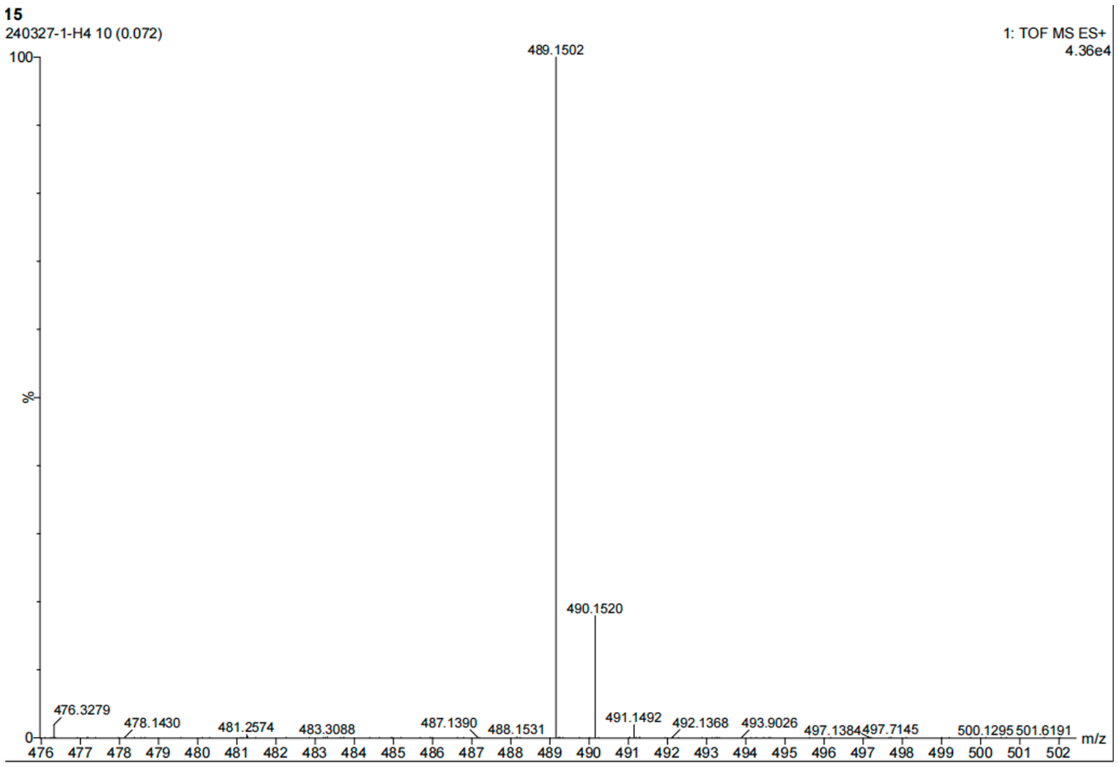

Figure S12 The HRMS spectrum of compound **7d**

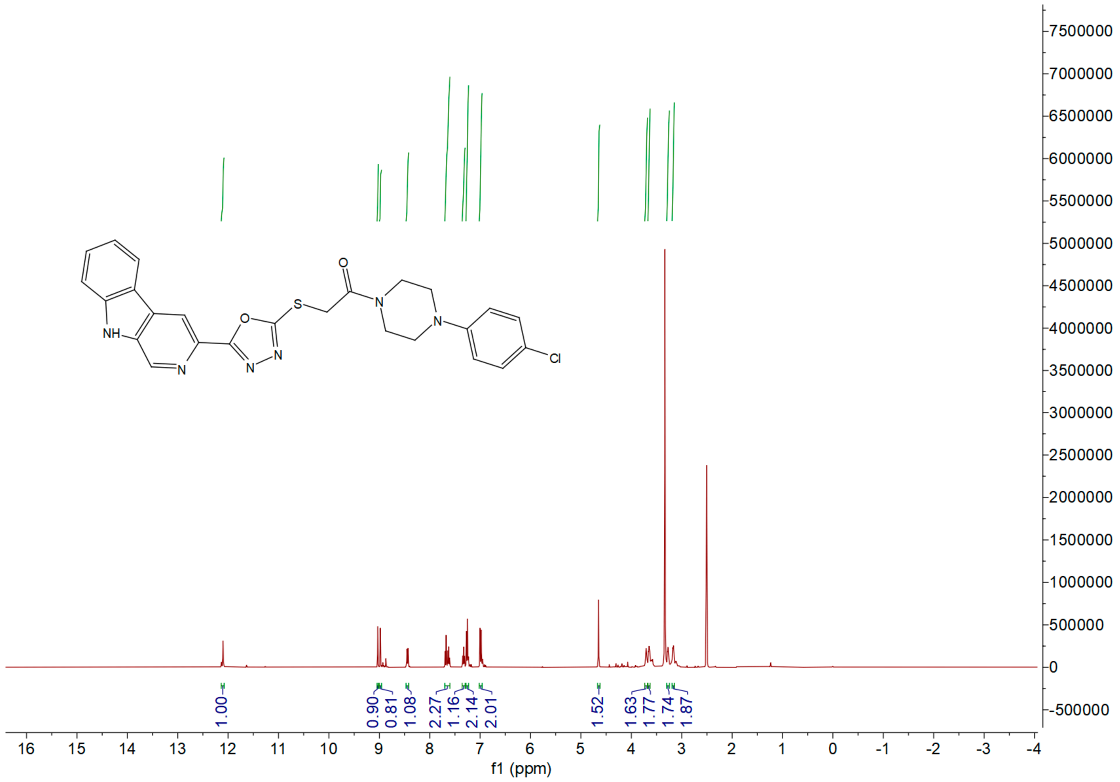

Figure S13 The ^{1}H NMR Spectrum of compound **7e**

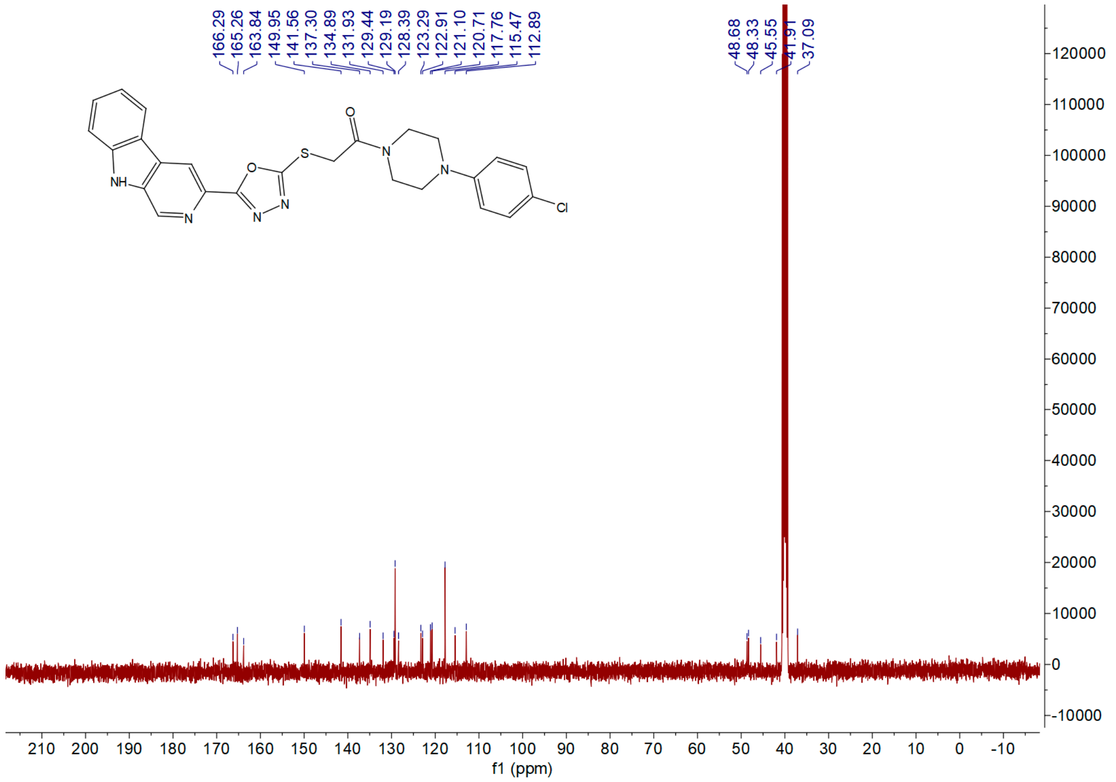

Figure S14 The ^{13}C NMR Spectrum of compound **7e**

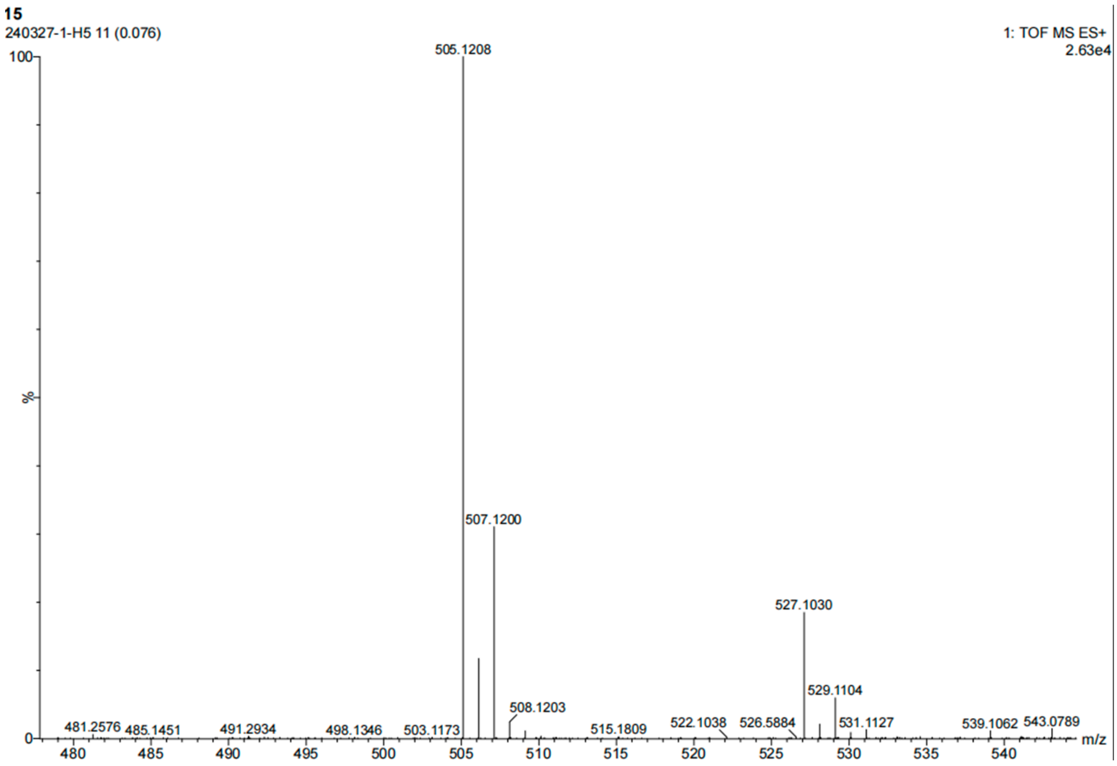

Figure S15 The HRMS spectrum of compound **7e**

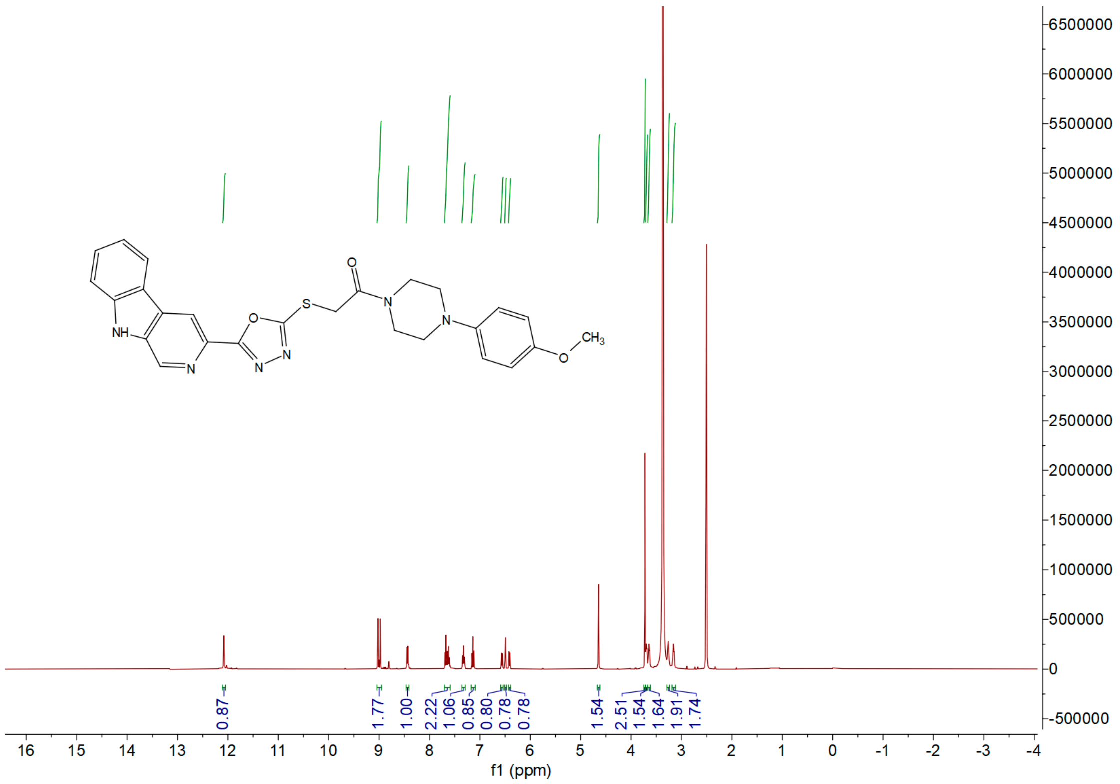

Figure S16 The ^{1}H NMR Spectrum of compound **7f**

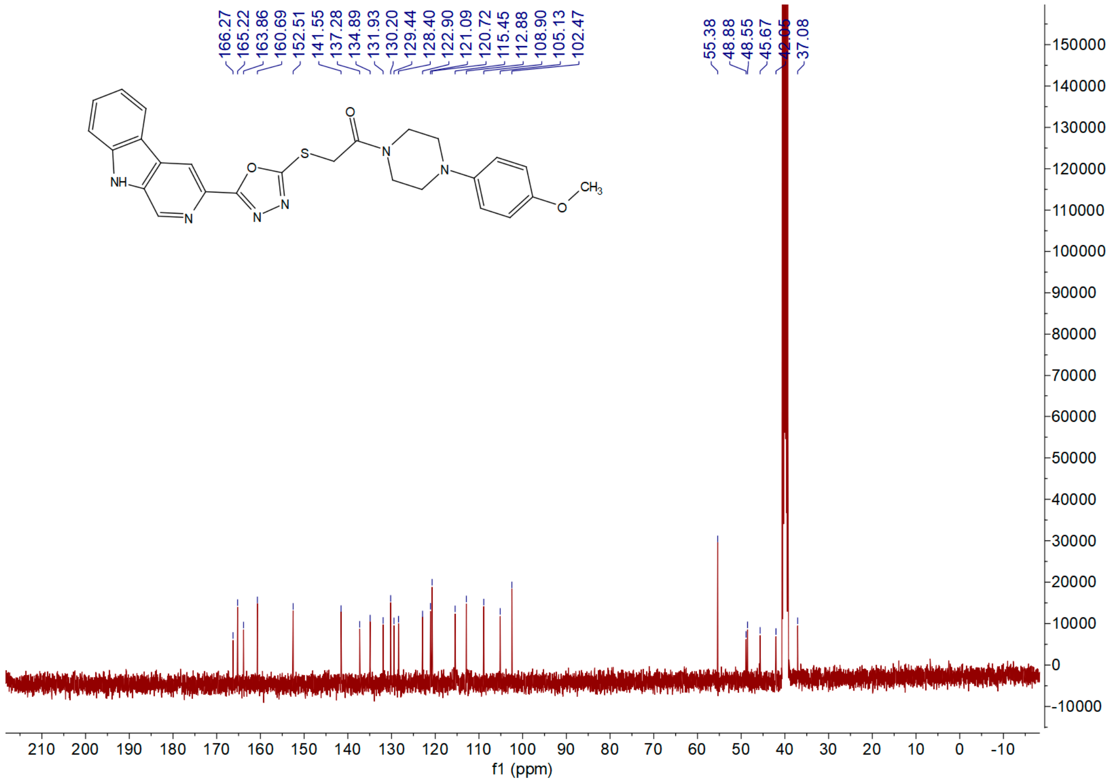

Figure S17 The ^{13}C NMR Spectrum of compound **7f**

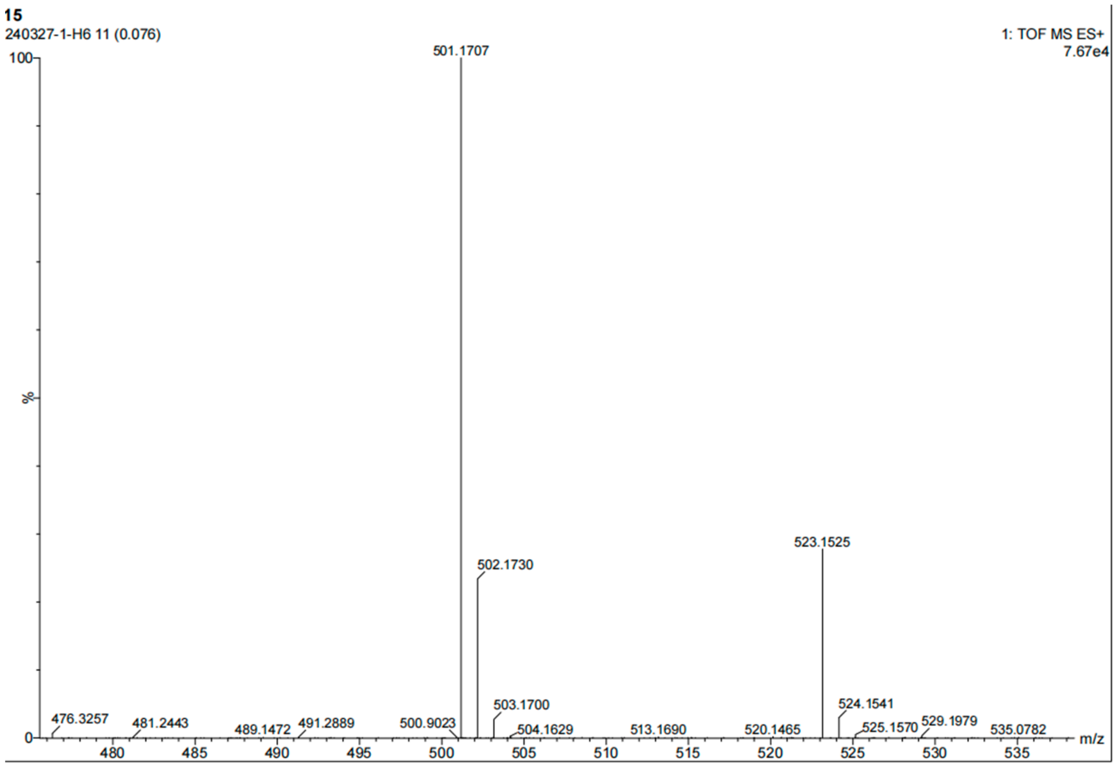

Figure S18 The HRMS spectrum of compound **7f**

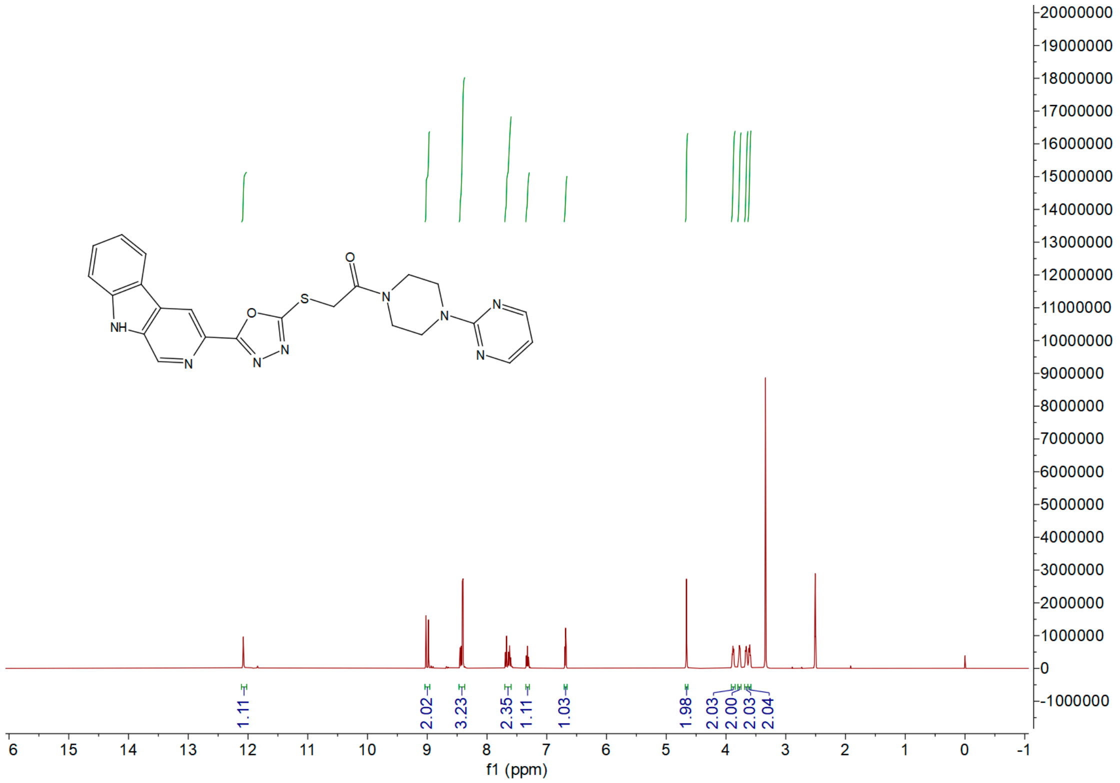

Figure S19 The ^{1}H NMR Spectrum of compound **7g**

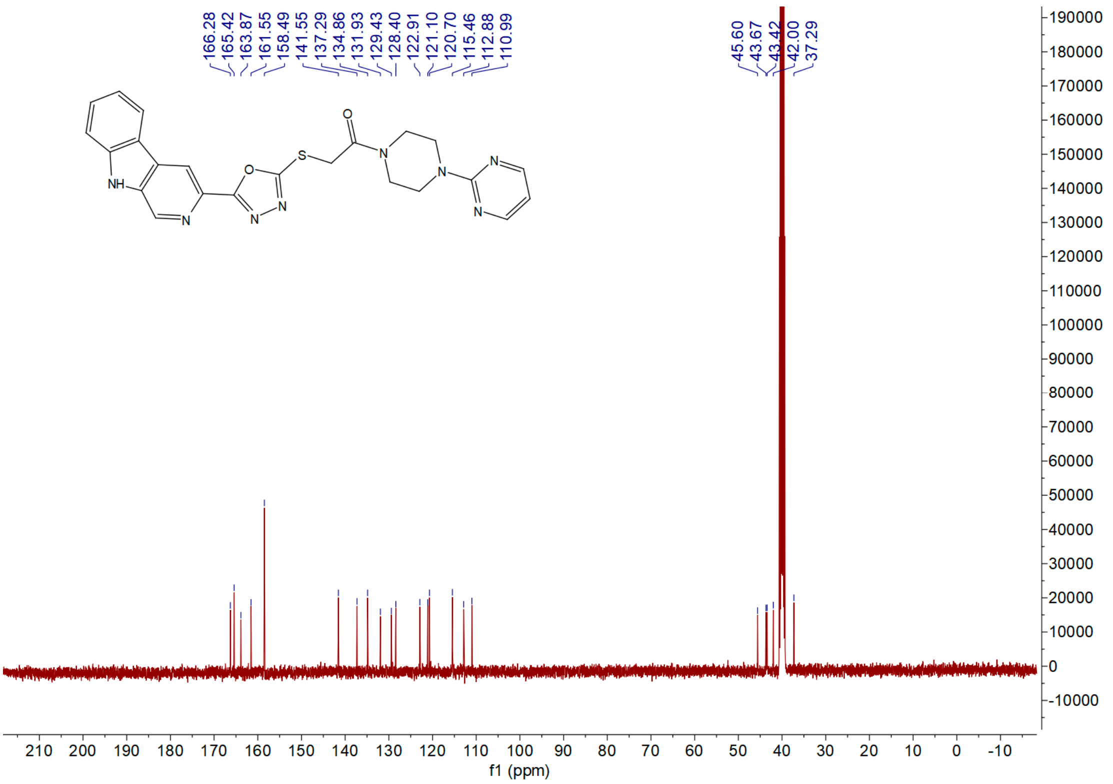

Figure S20 The ^{13}C NMR Spectrum of compound **7g**

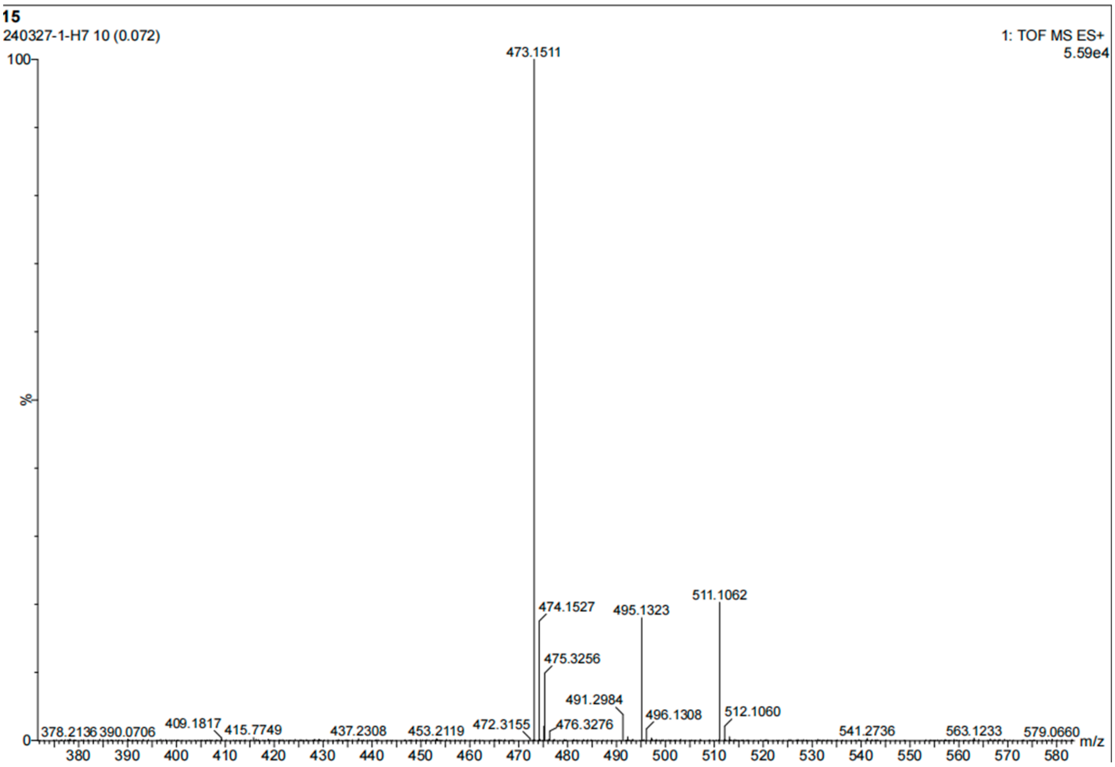

Figure S21 The HRMS spectrum of compound **7g**

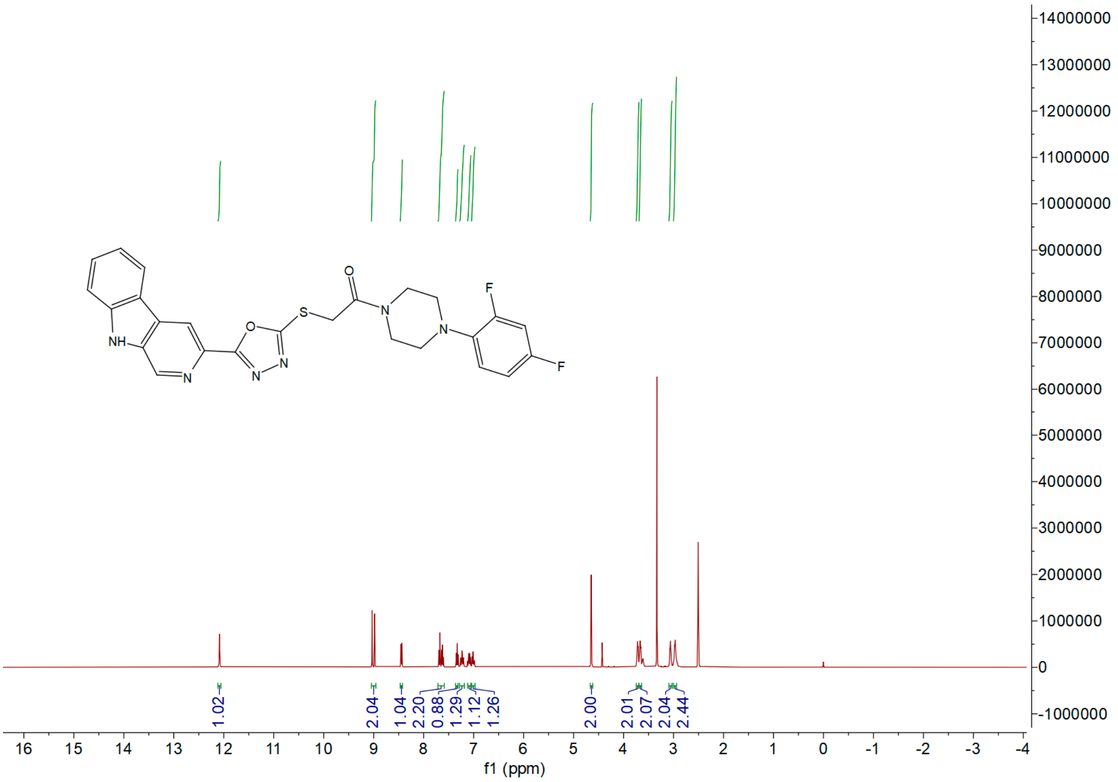

Figure S22 The ^{1}H NMR Spectrum of compound **7h**

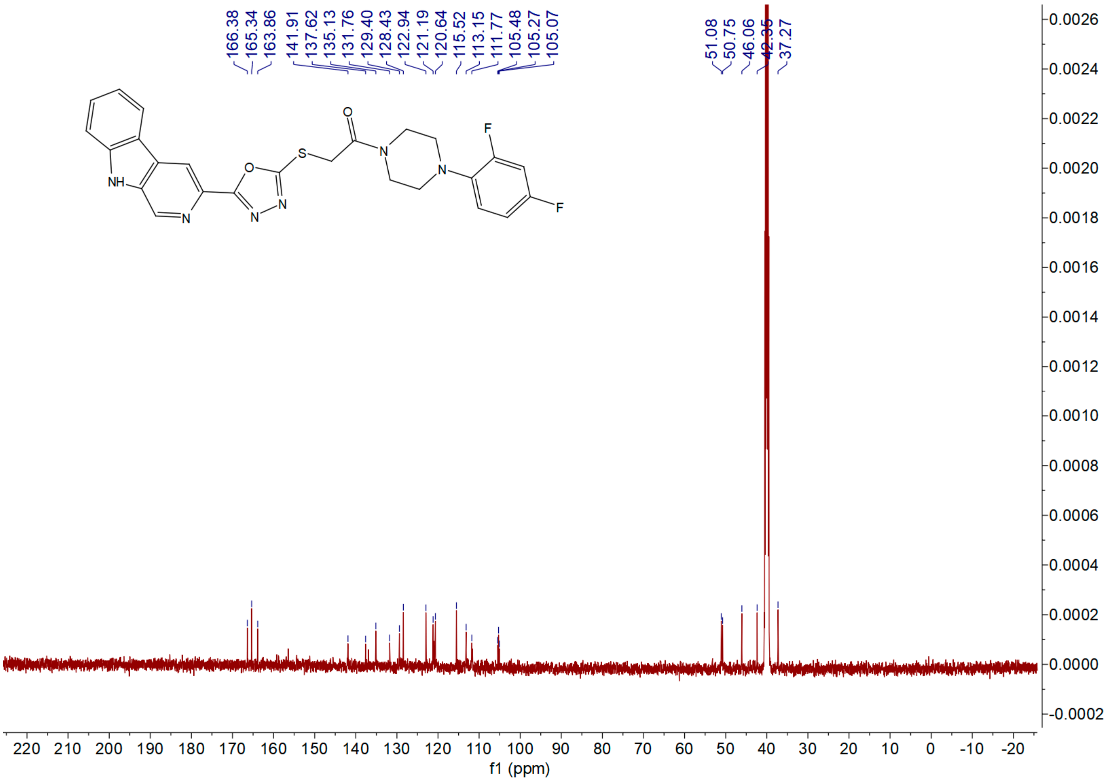

Figure S23 The ^{13}C NMR Spectrum of compound **7h**

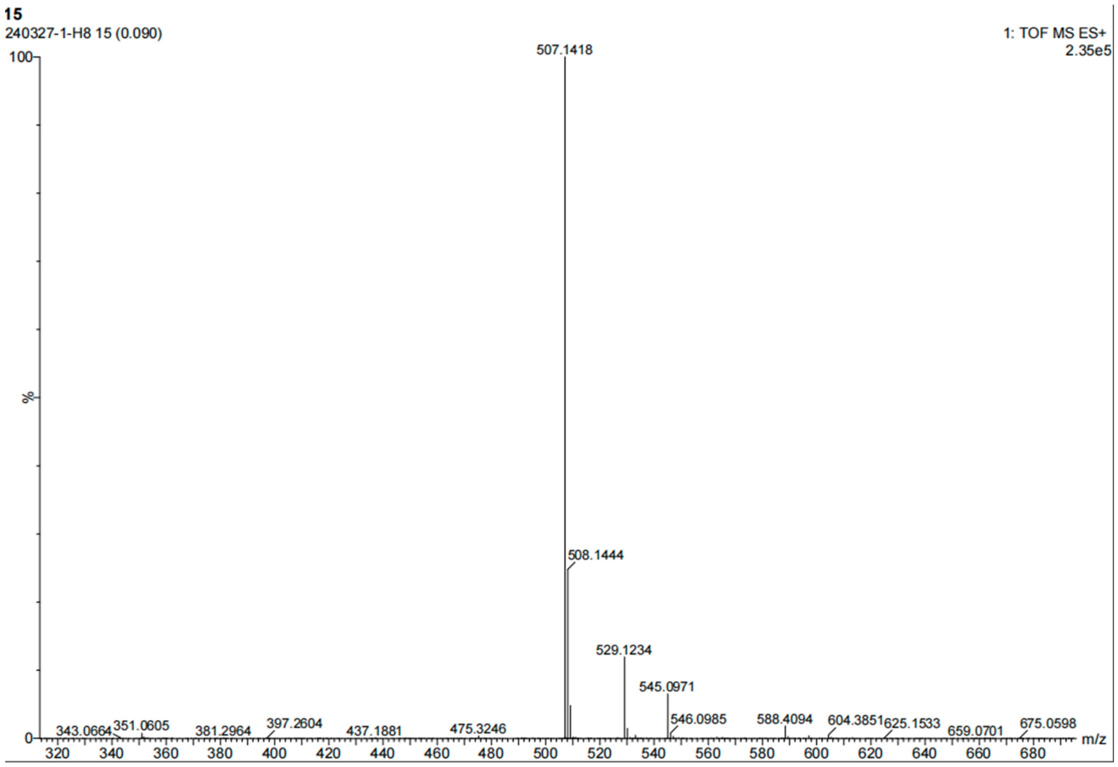

Figure S24 The HRMS spectrum of compound **7h**

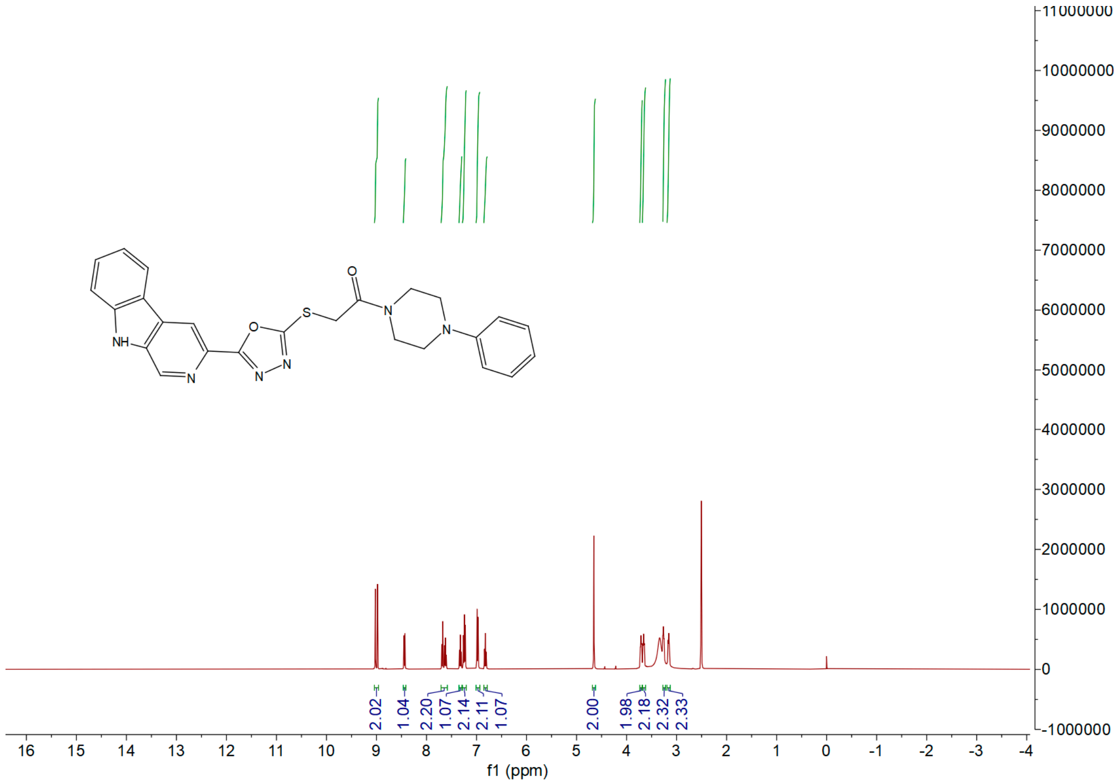

Figure S25 The ^{1}H NMR Spectrum of compound **7i**

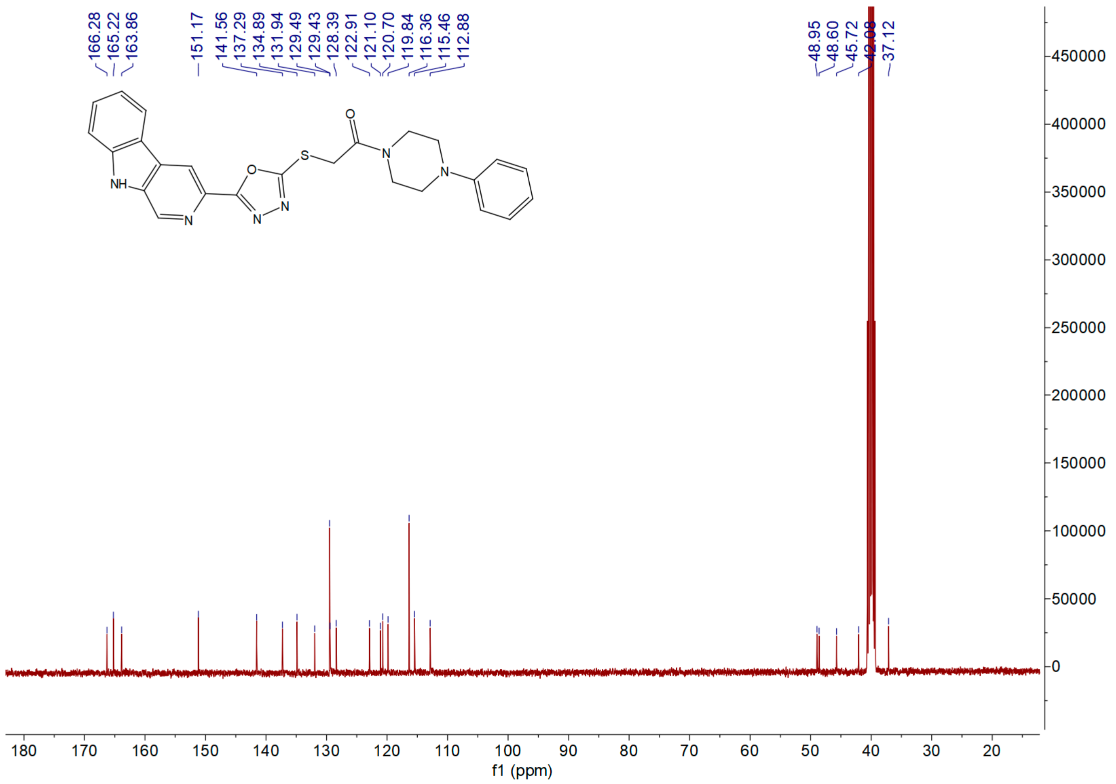

Figure S26 The ^{13}C NMR Spectrum of compound **7i**

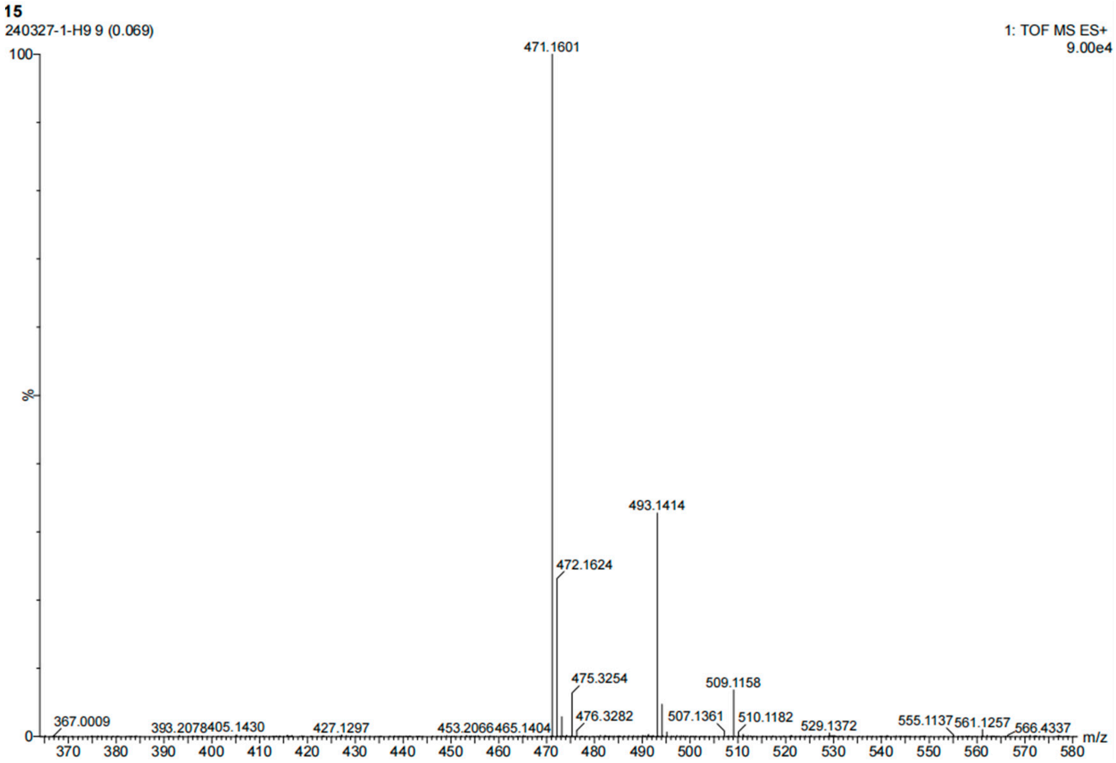

Figure S27 The HRMS spectrum of compound **7i**

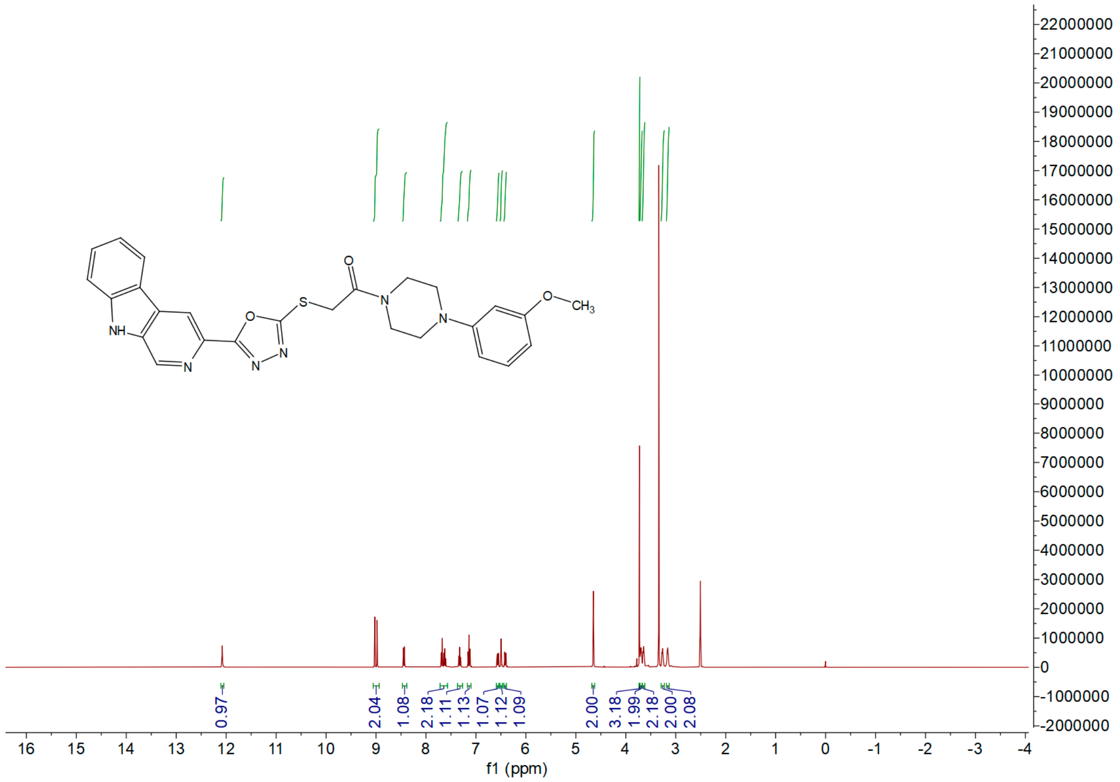

Figure S28 The ^{1}H NMR Spectrum of compound **7J**

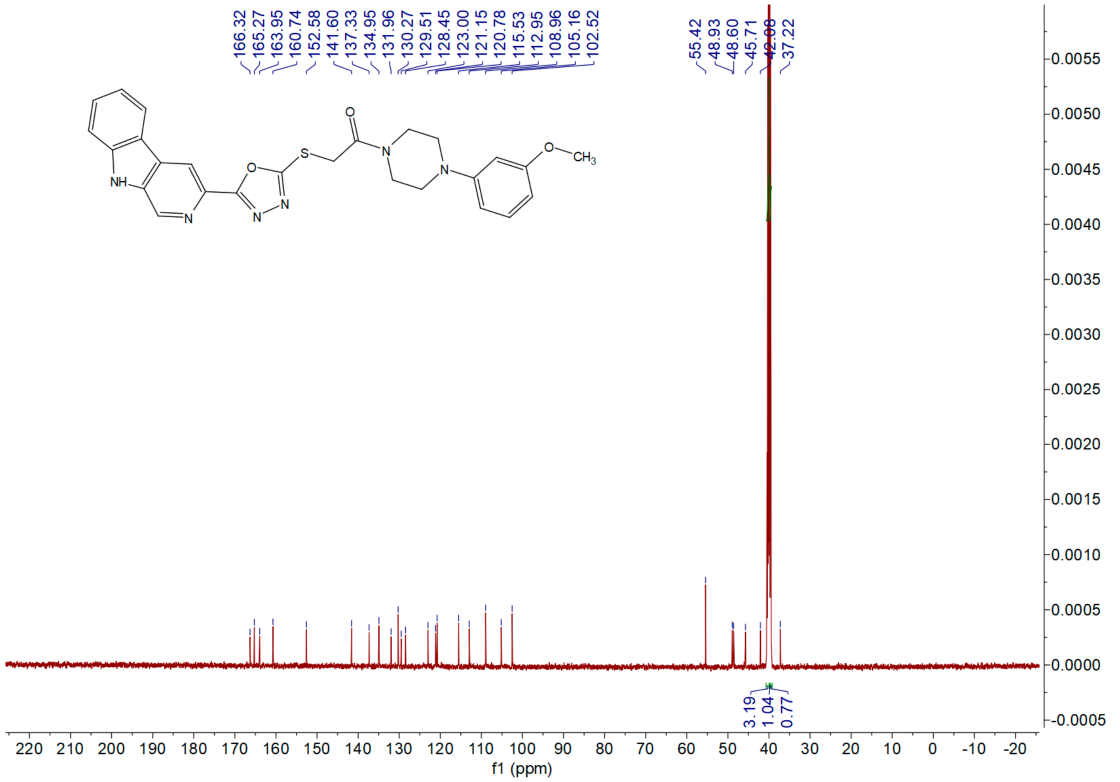

Figure S29 The ^{13}C NMR Spectrum of compound **7J**

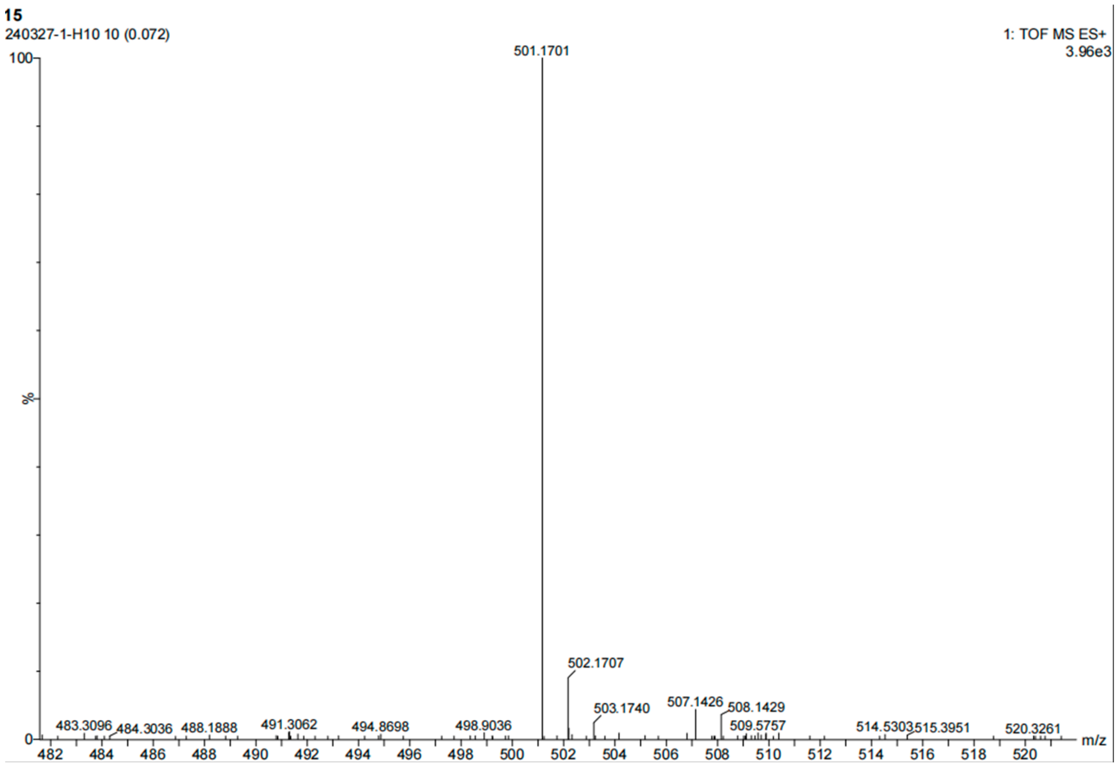

Figure S30 The HRMS spectrum of compound **7J**

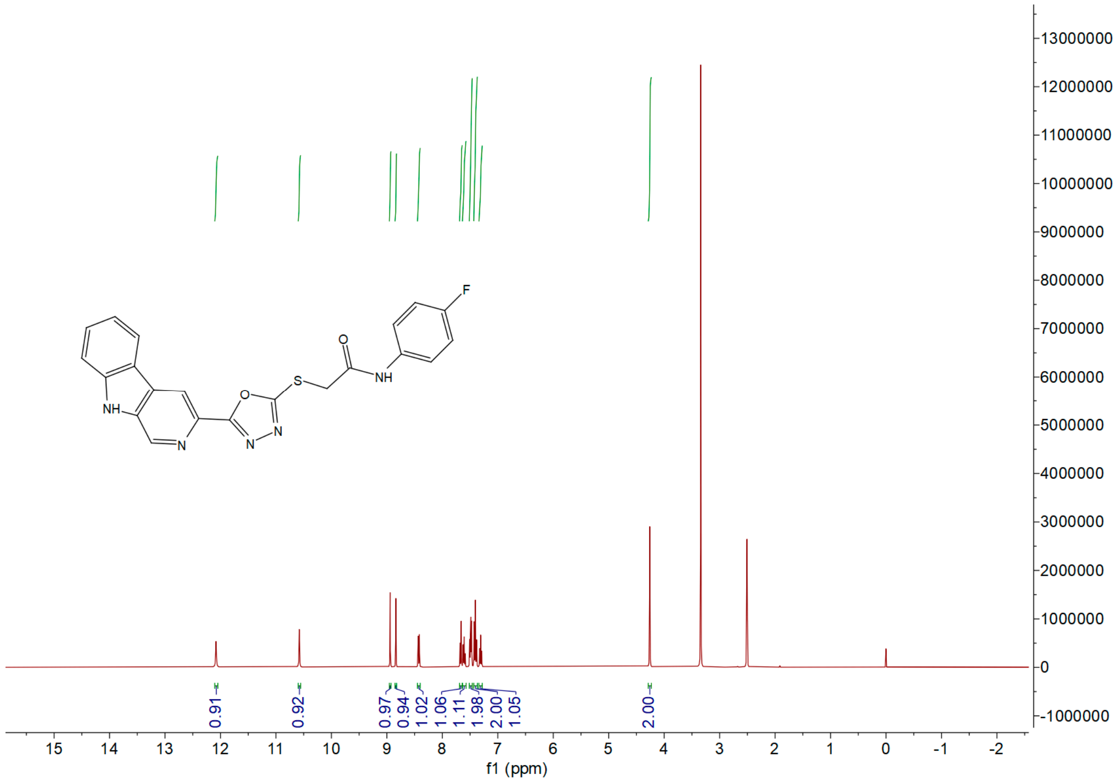

Figure S31 The ^{1}H NMR Spectrum of compound **7k**

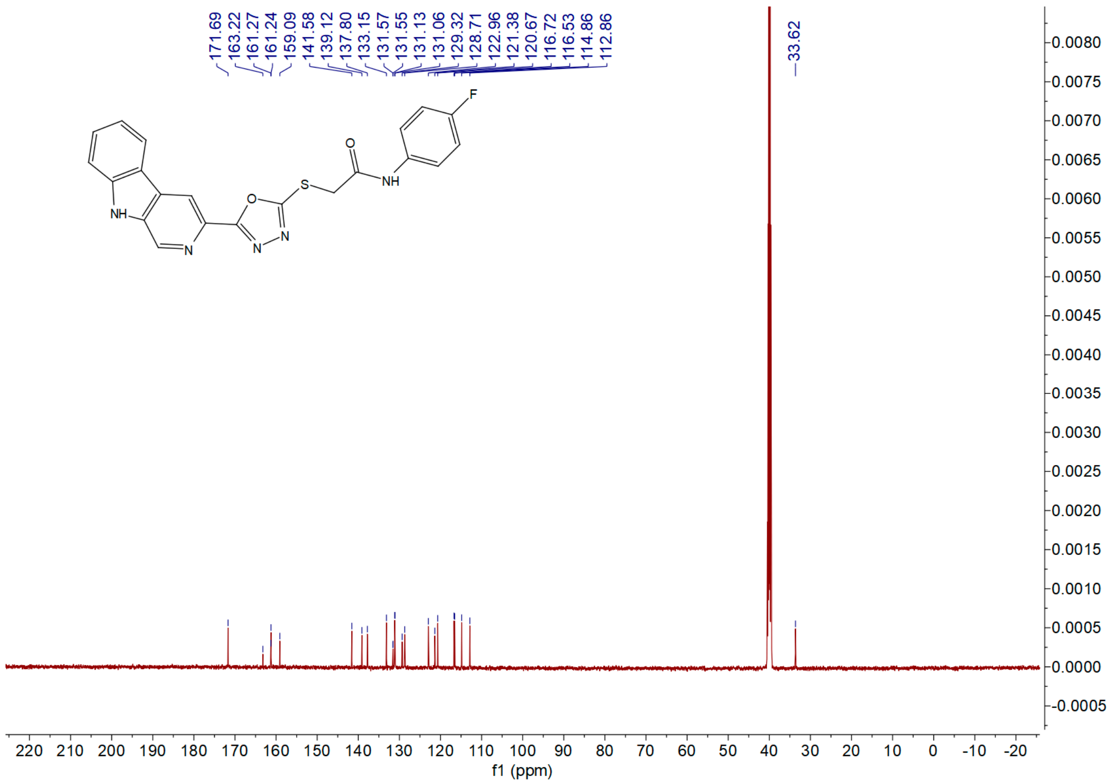

Figure S32 The ^{13}C NMR Spectrum of compound **7k**

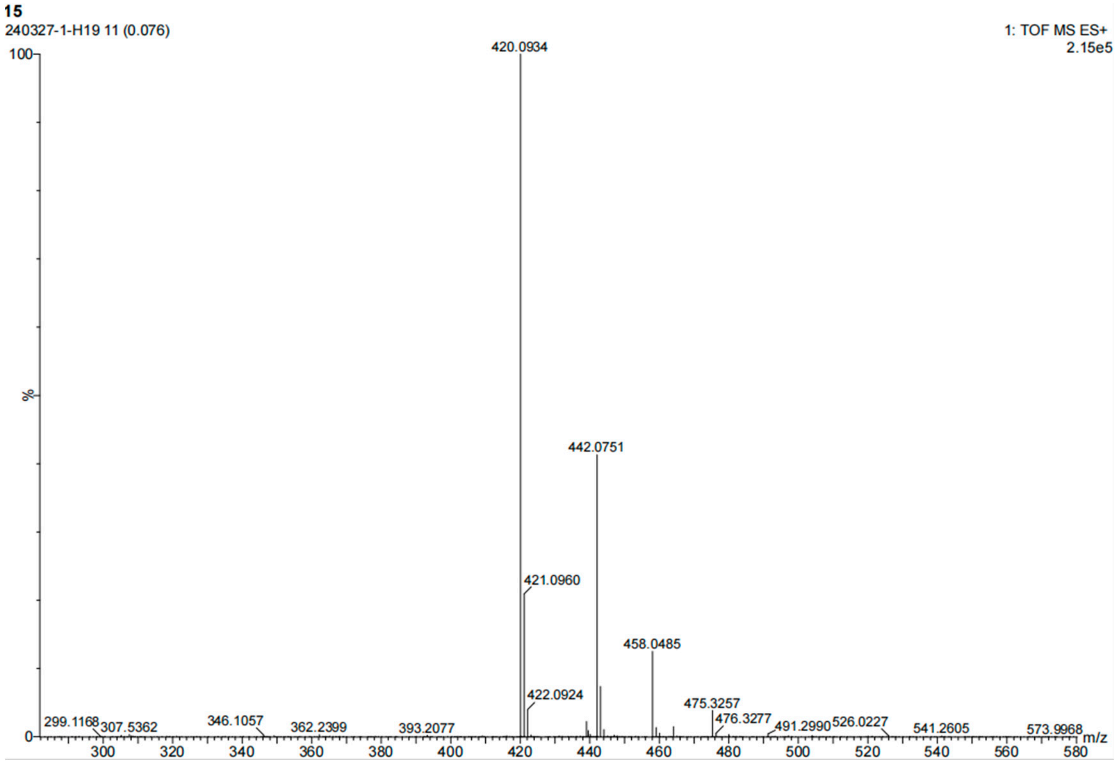

Figure S33 The HRMS spectrum of compound **7k**

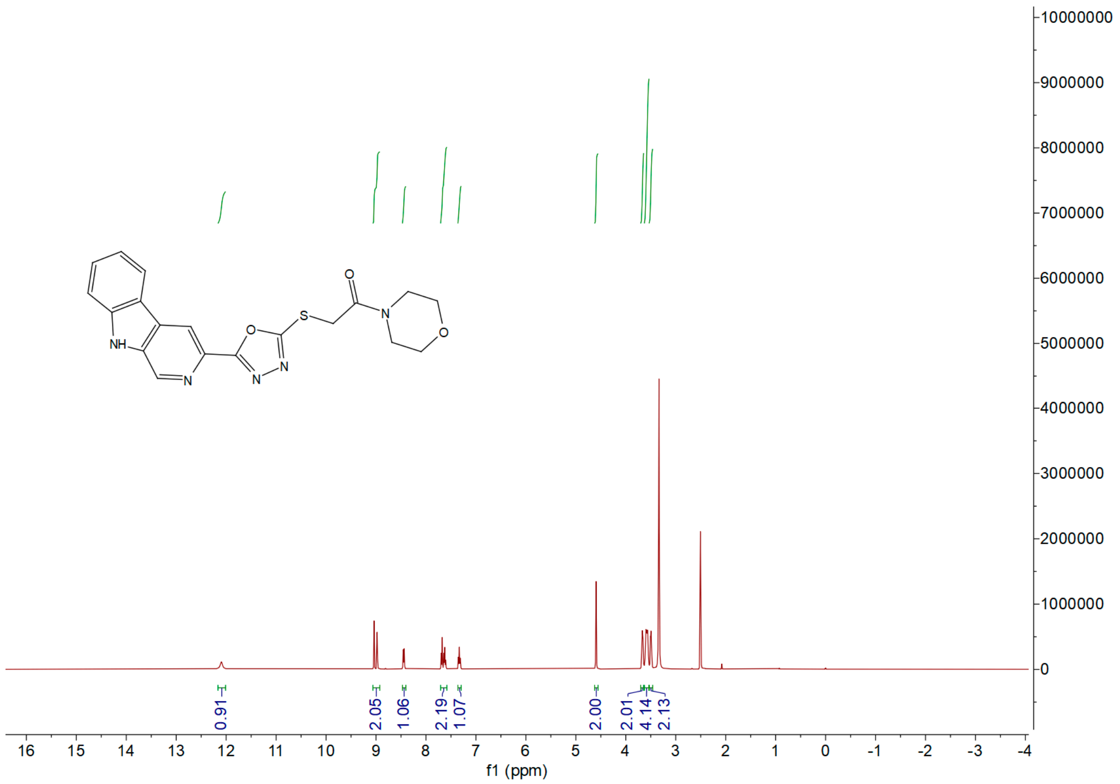

Figure 34 The ^{1}H NMR Spectrum of compound **7l**

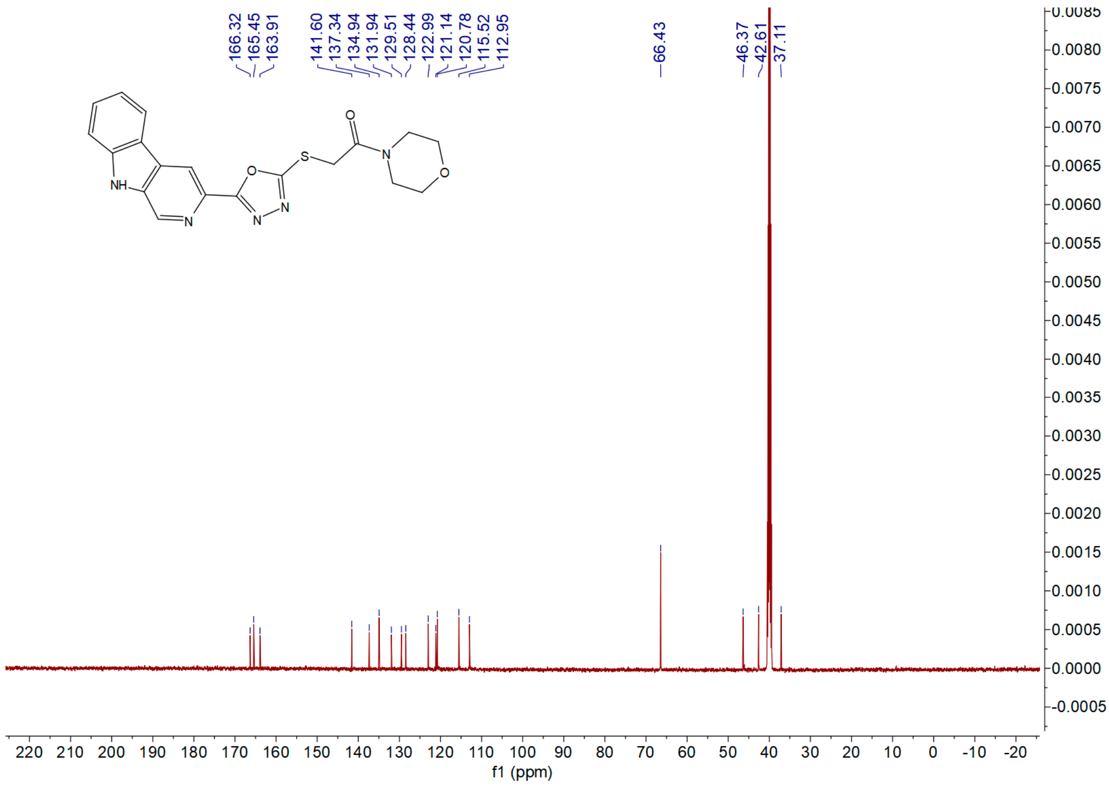

Figure S35 The ^{13}C NMR Spectrum of compound **7l**

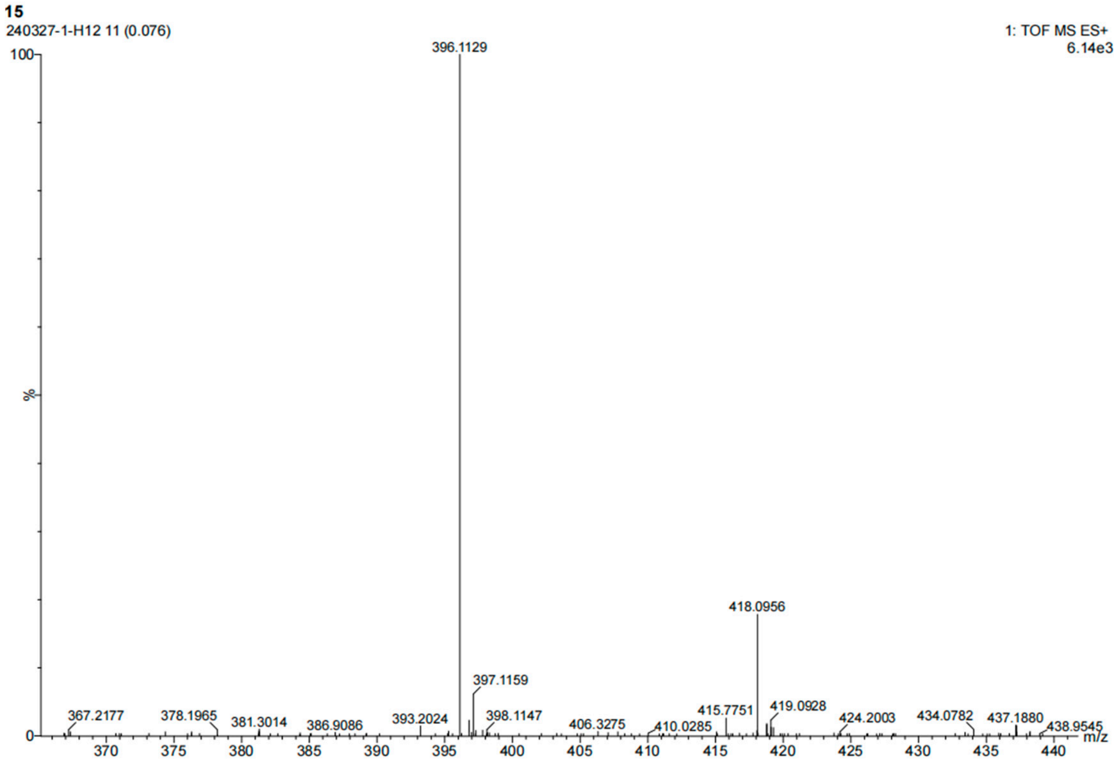

Figure S36 The HRMS spectrum of compound **7l**

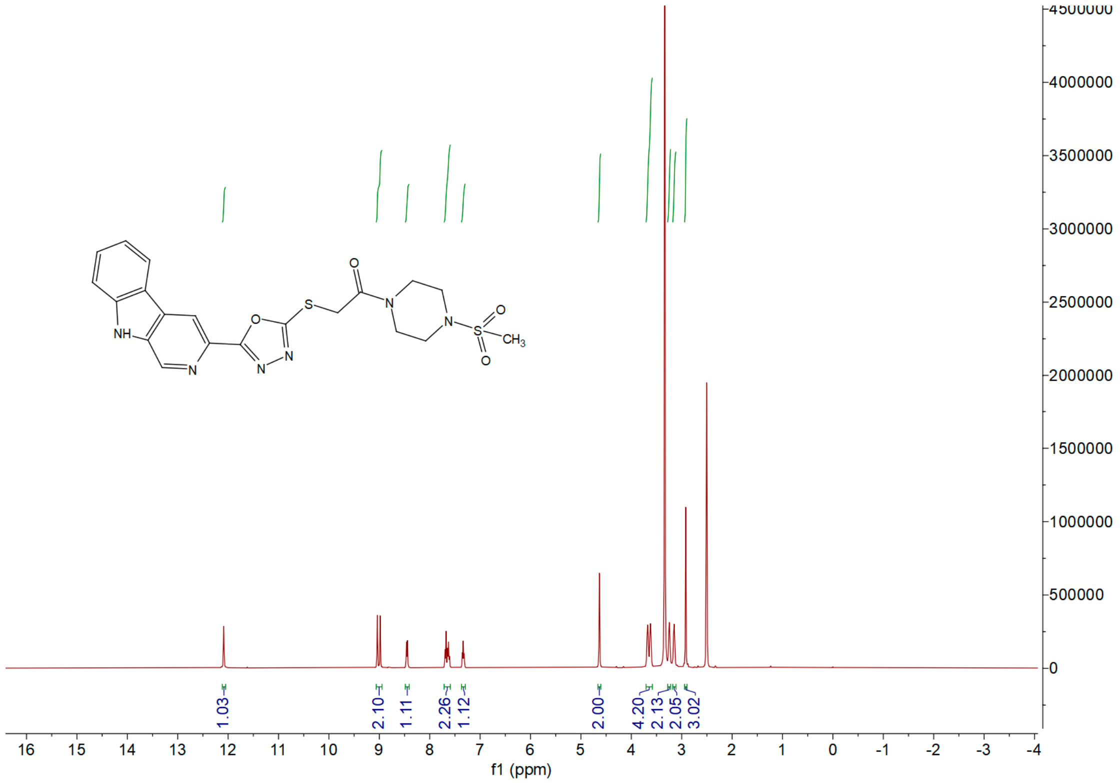

Figure S37 The ^{1}H NMR Spectrum of compound **7m**

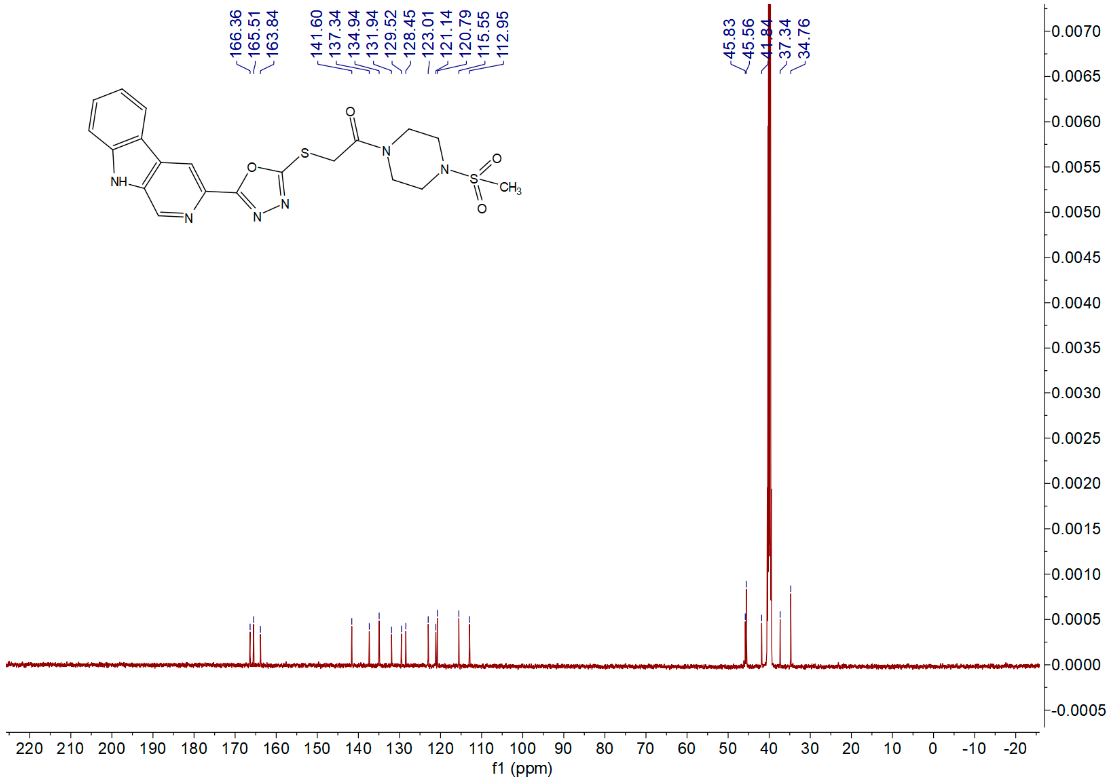

Figure S38 The ^{13}C NMR Spectrum of compound **7m**

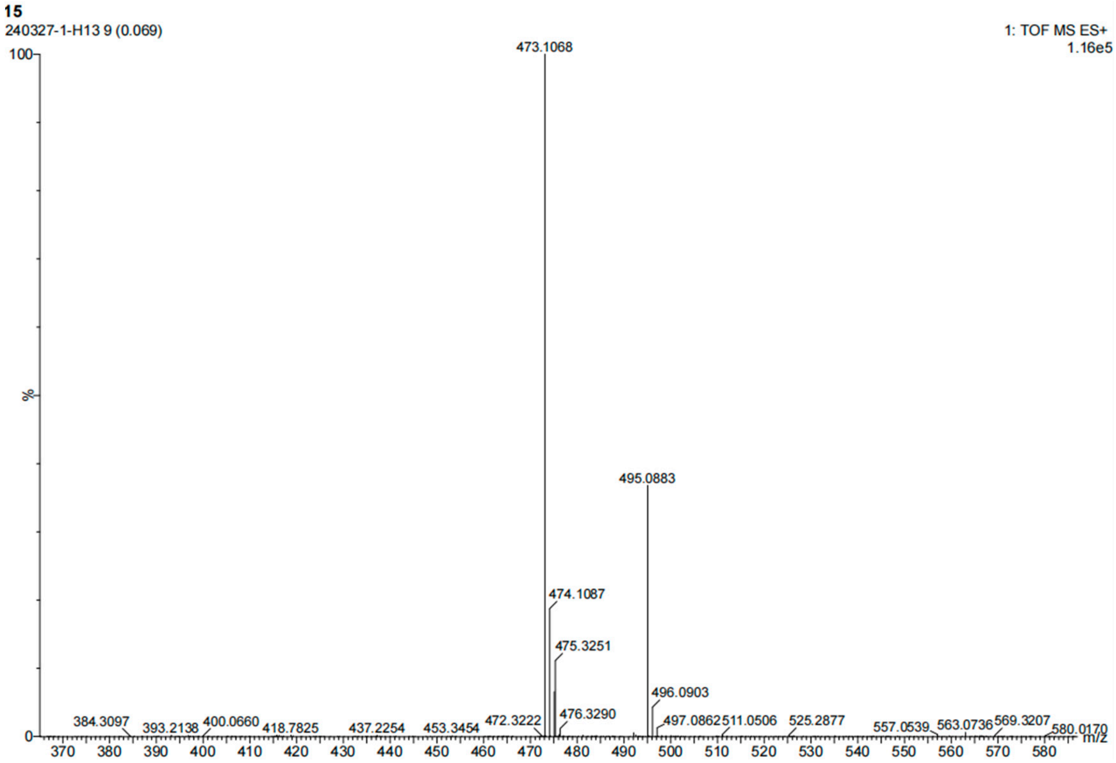

Figure S39 The HRMS spectrum of compound **7m**

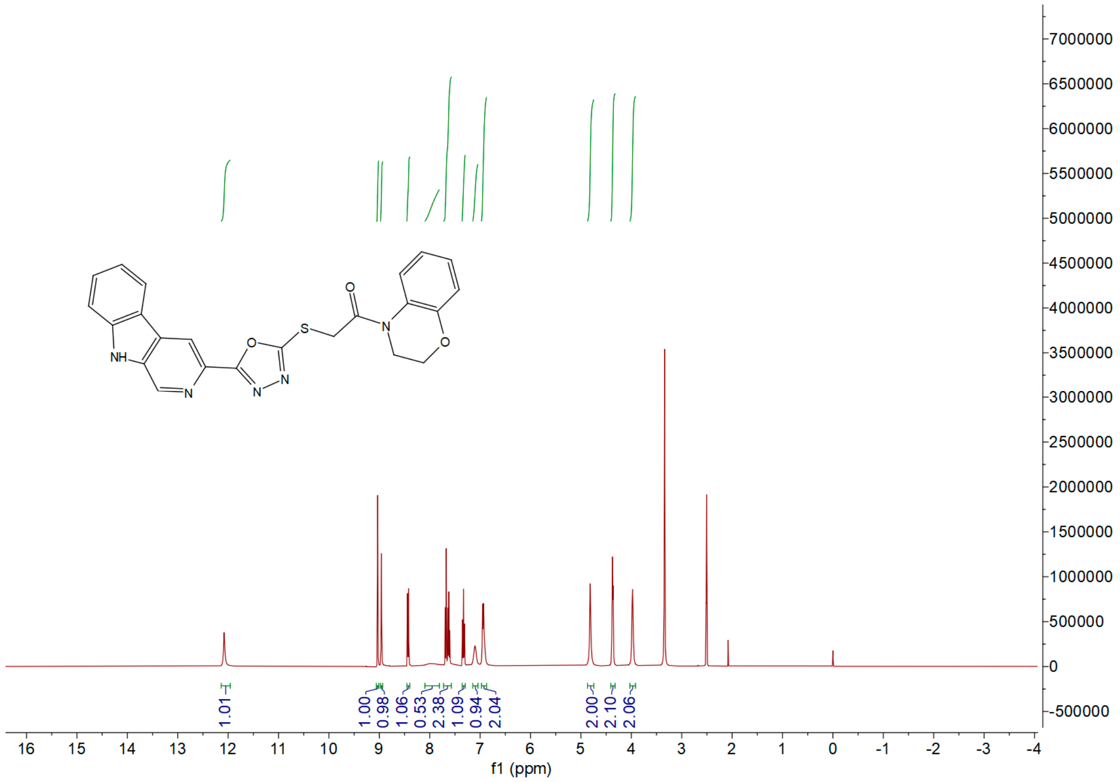

Figure S40 The ^{1}H NMR Spectrum of compound **7n**

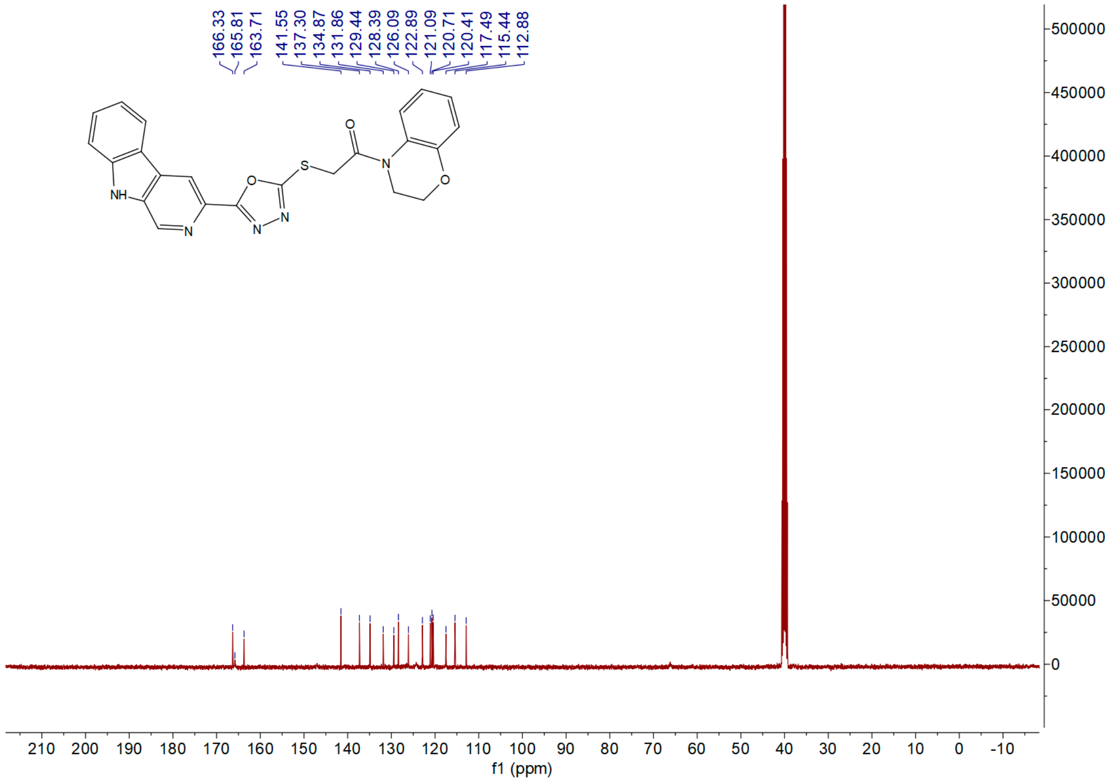

Figure S41 The ^{13}C NMR Spectrum of compound **7n**

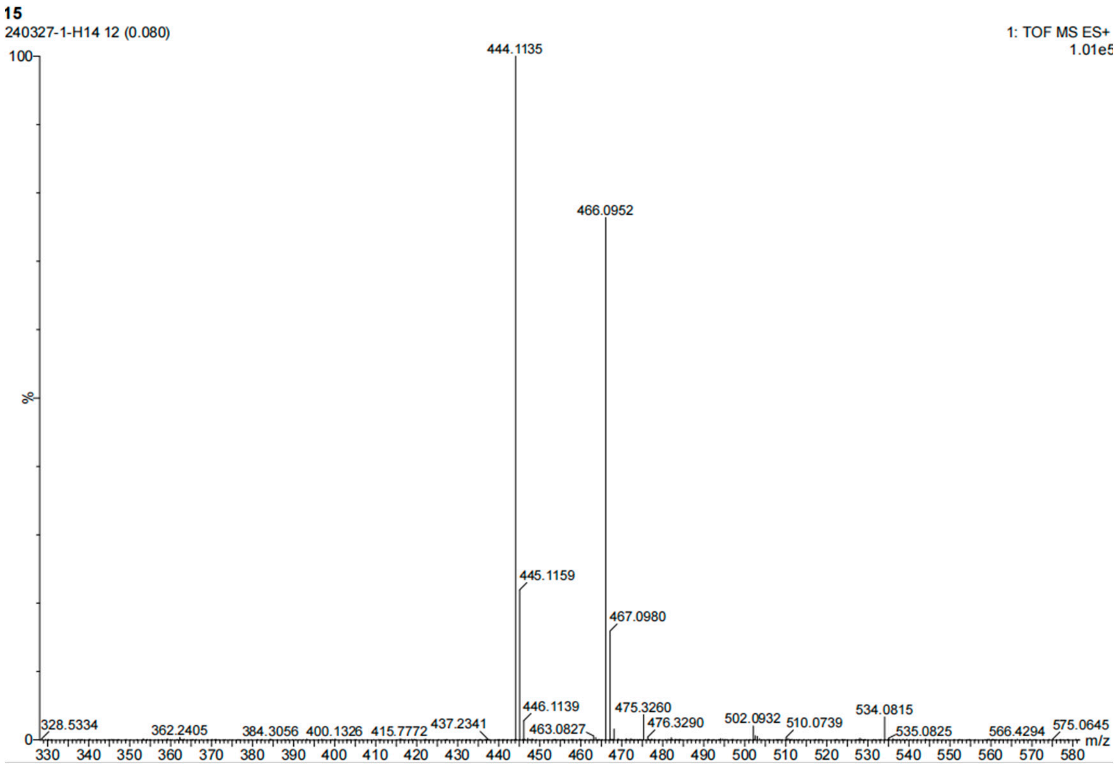

Figure S42 The HRMS spectrum of compound **7n**

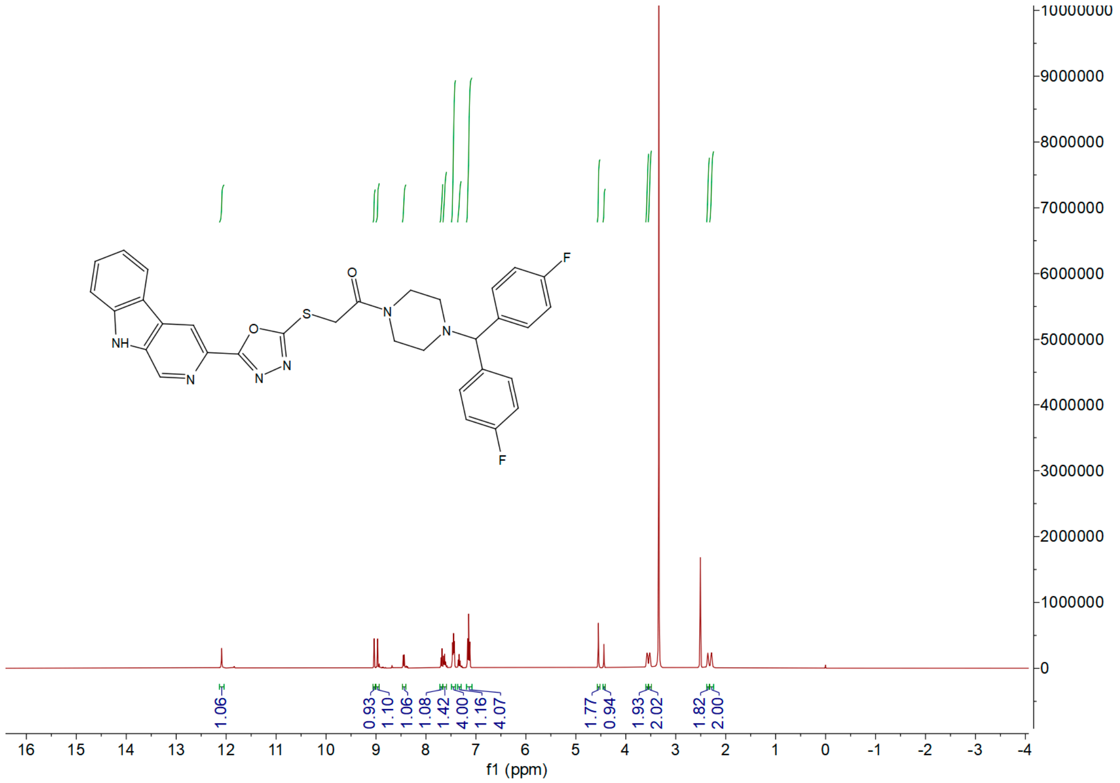

Figure S43 The ^{1}H NMR Spectrum of compound **7o**

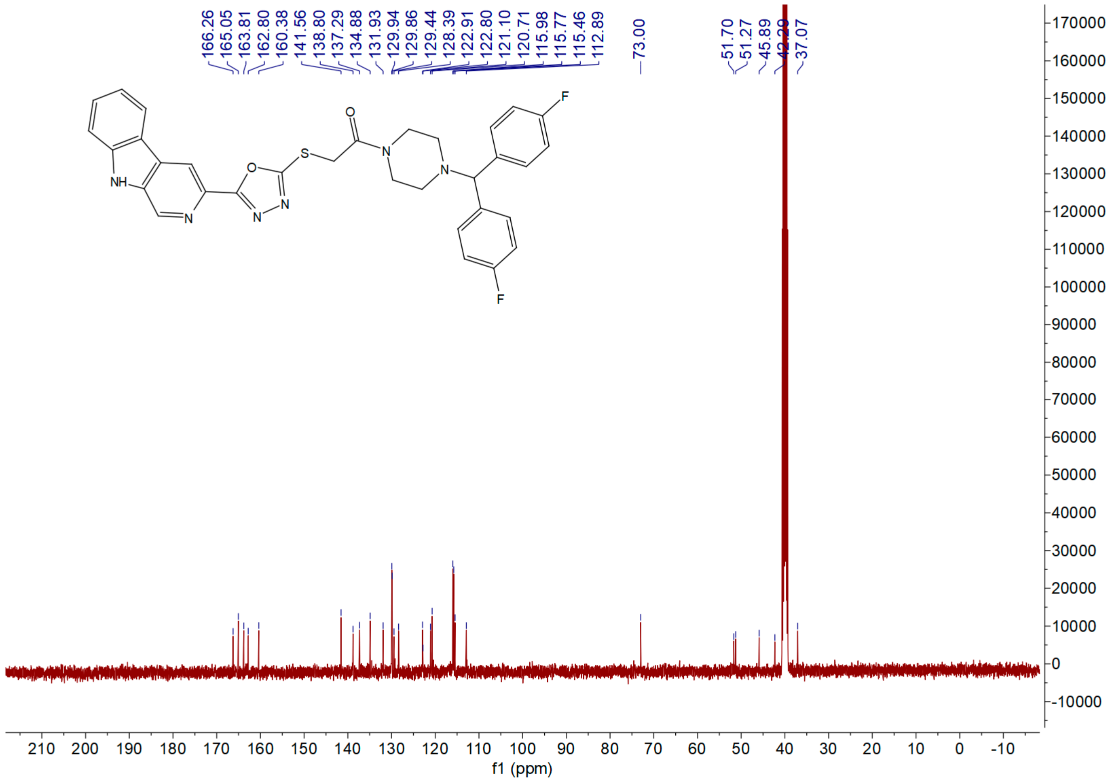

Figure S44 The ^{13}C NMR Spectrum of compound **7o**

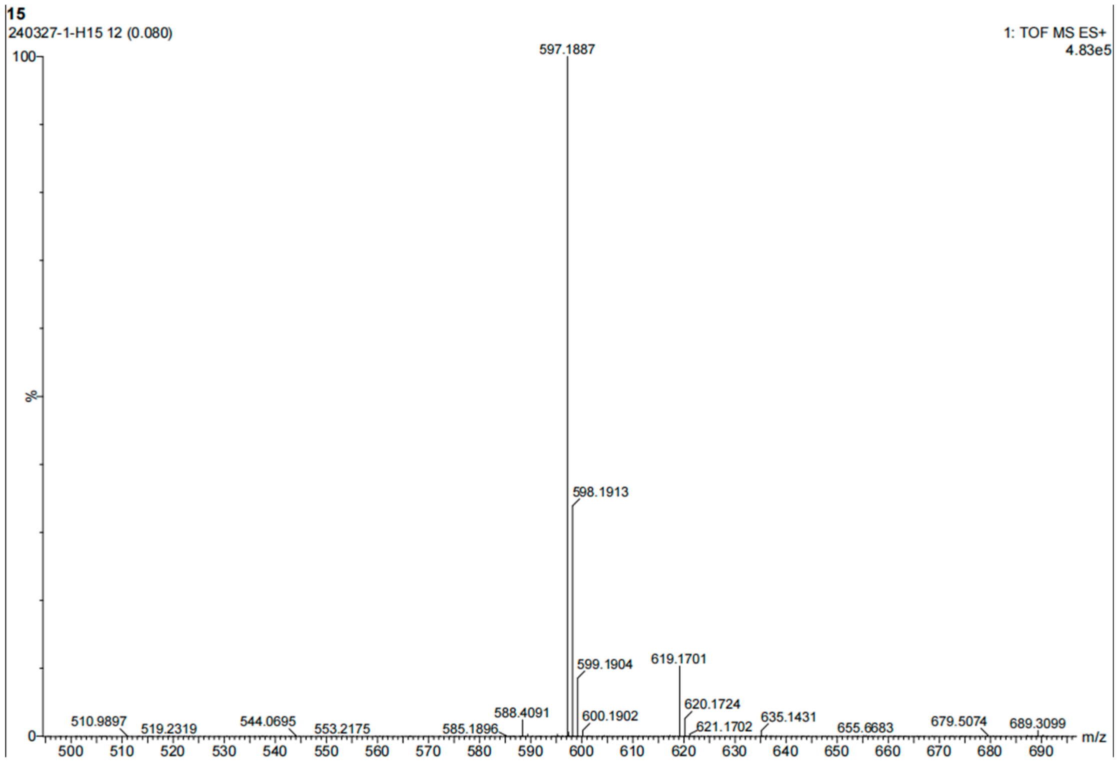

Figure S45 The HRMS spectrum of compound **7o**

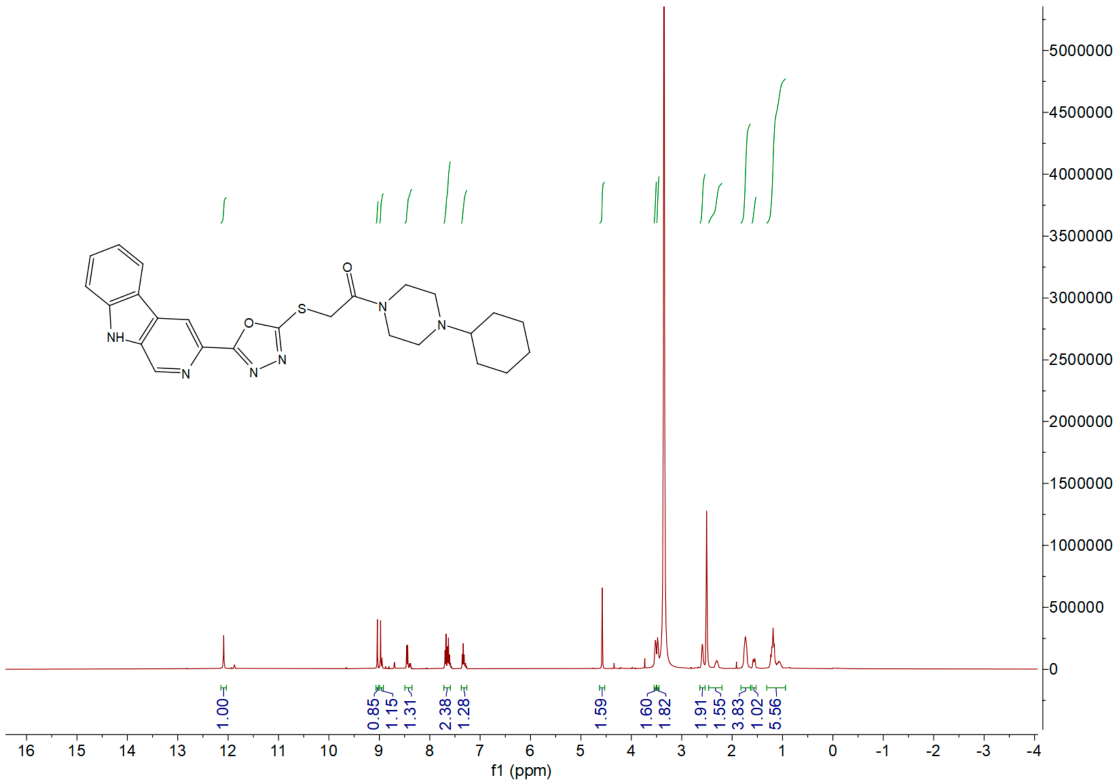

Figure S46 The ^{1}H NMR Spectrum of compound **7p**

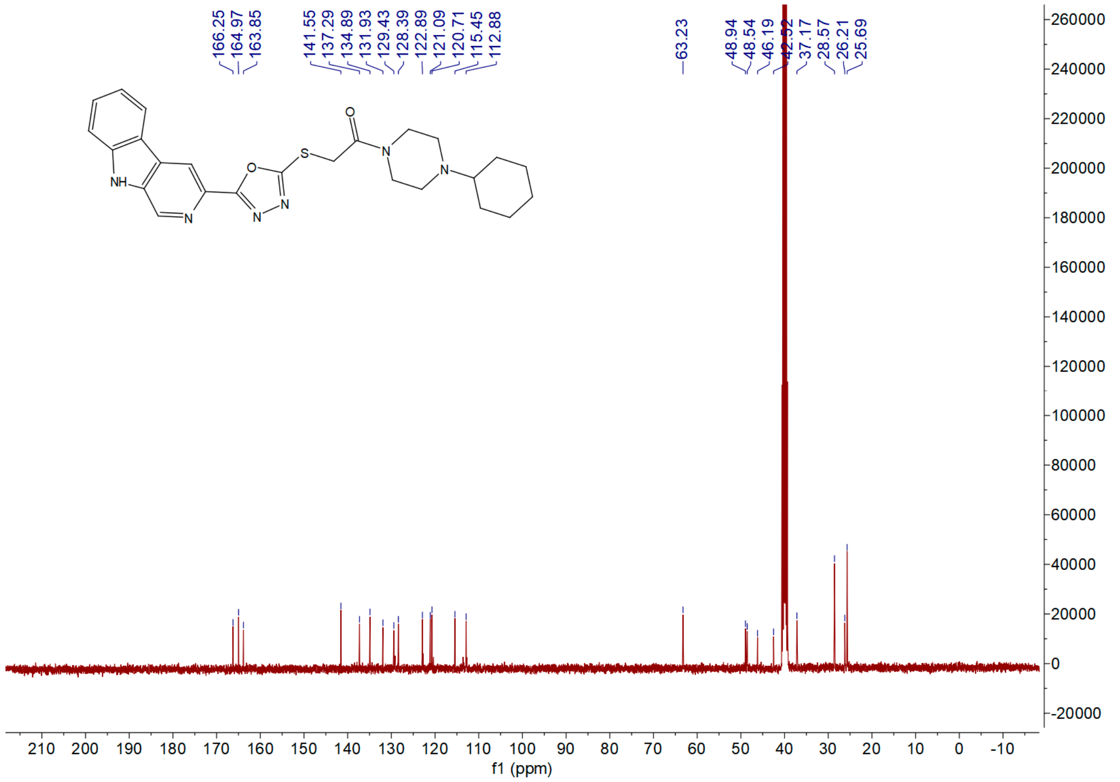

Figure S47 The ^{13}C NMR Spectrum of compound **7p**

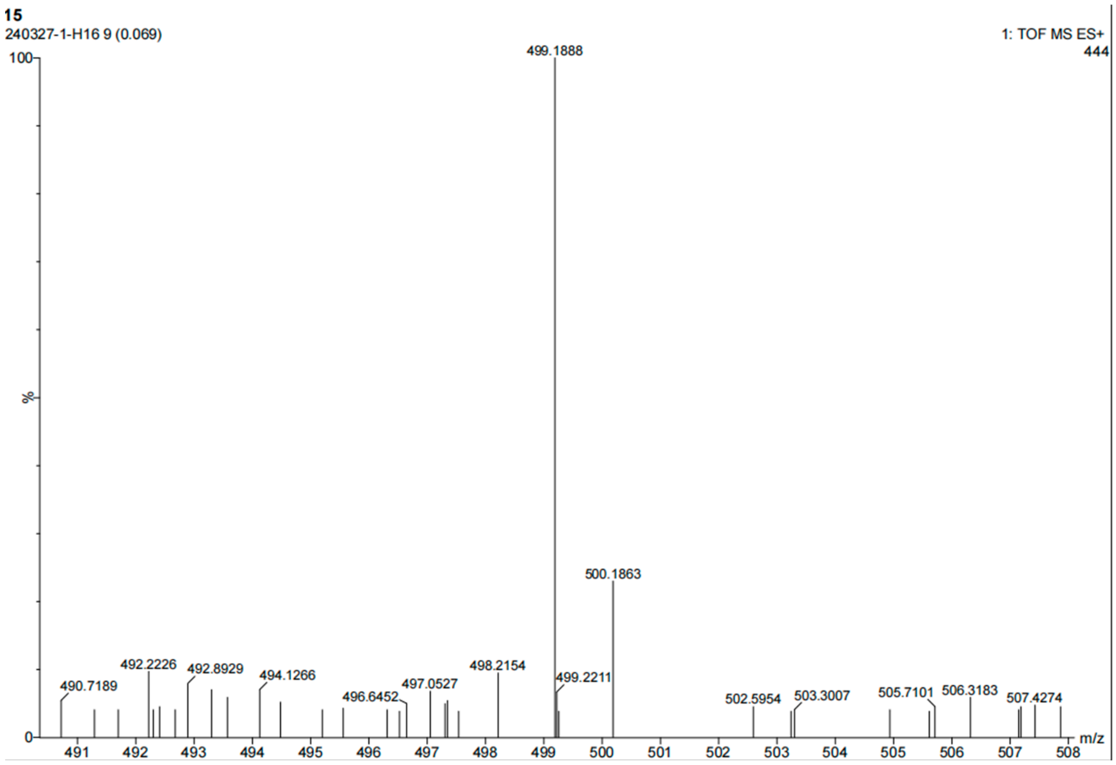

Figure S48 The HRMS spectrum of compound **7p**

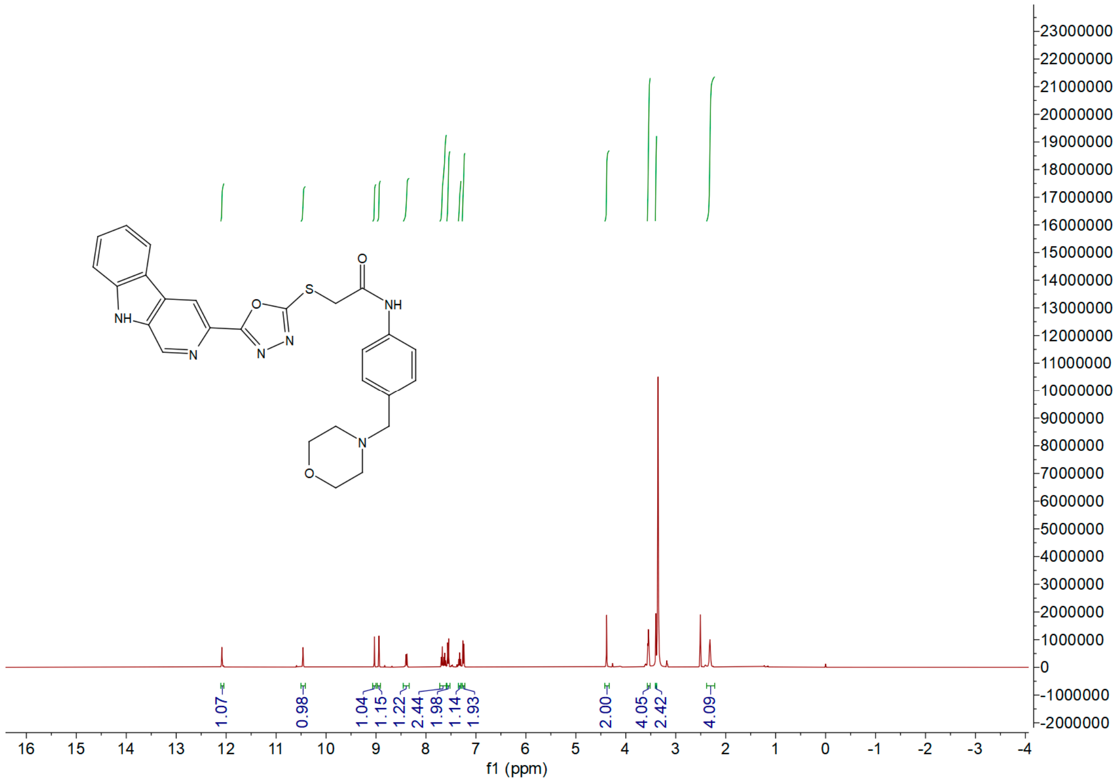

Figure S49 The ^{1}H NMR Spectrum of compound **7q**

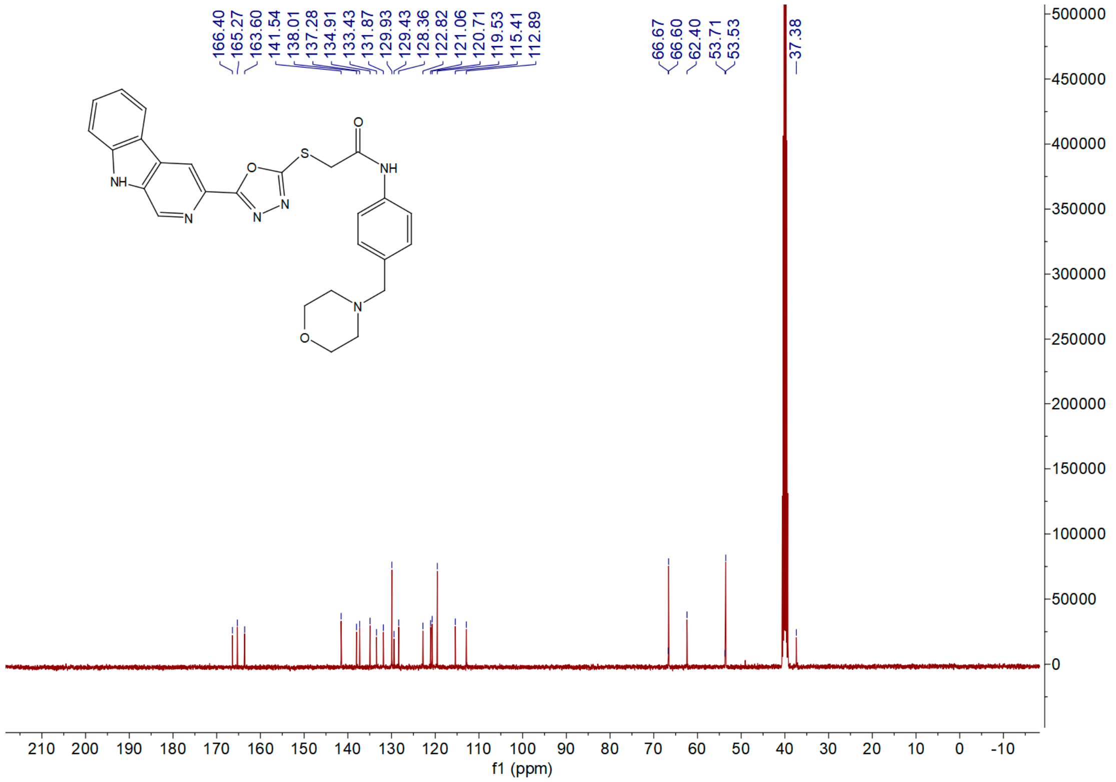

Figure S50 The ^{13}C NMR Spectrum of compound **7q**

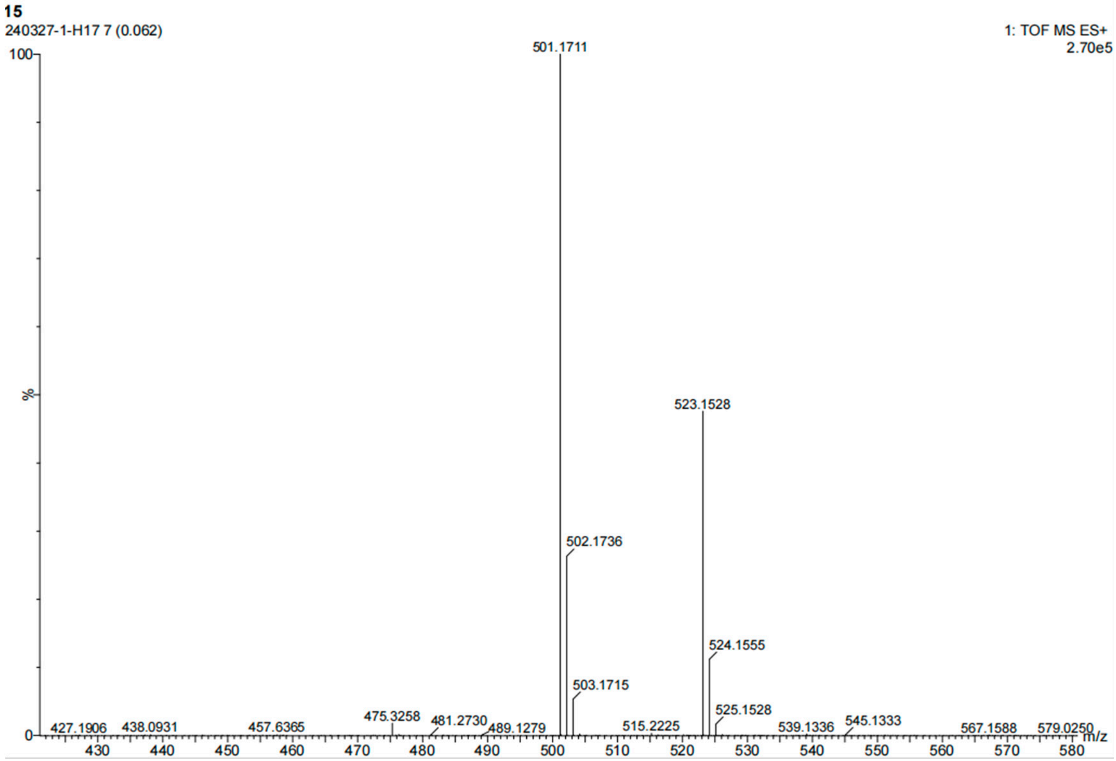

Figure S51 The HRMS spectrum of compound **7q**

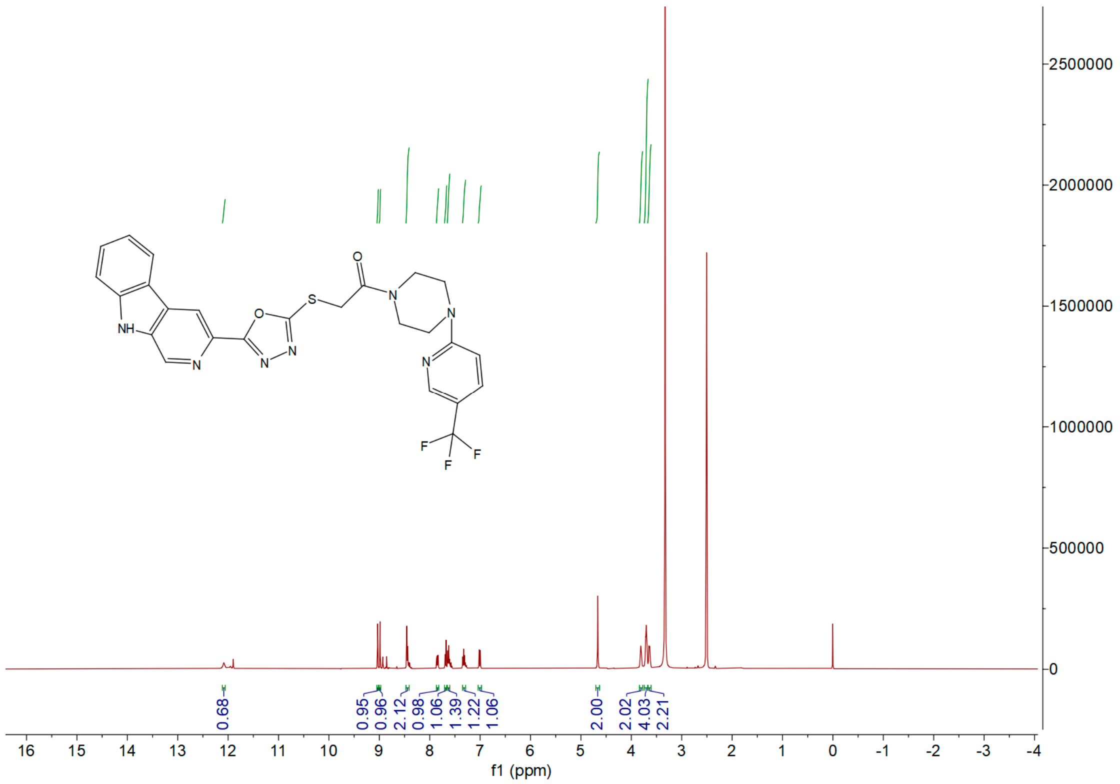

Figure S52 The ^{1}H NMR Spectrum of compound **7r**

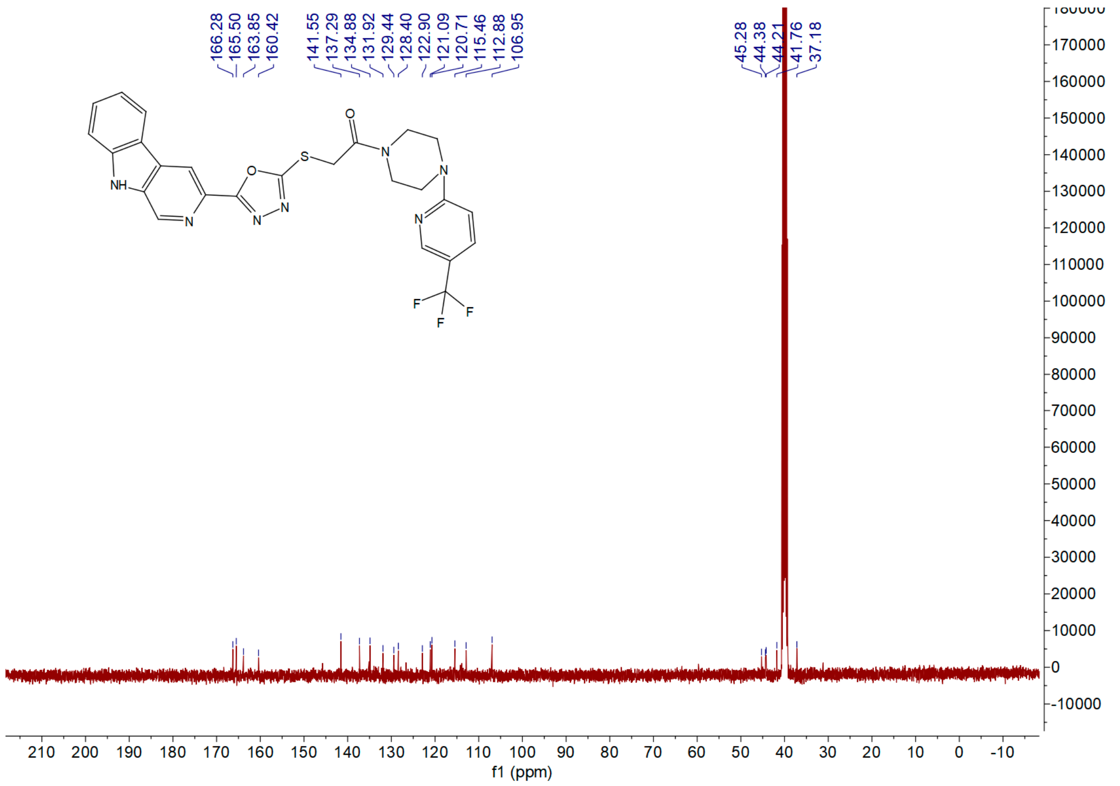

Figure S53 The ^{13}C NMR Spectrum of compound **7r**

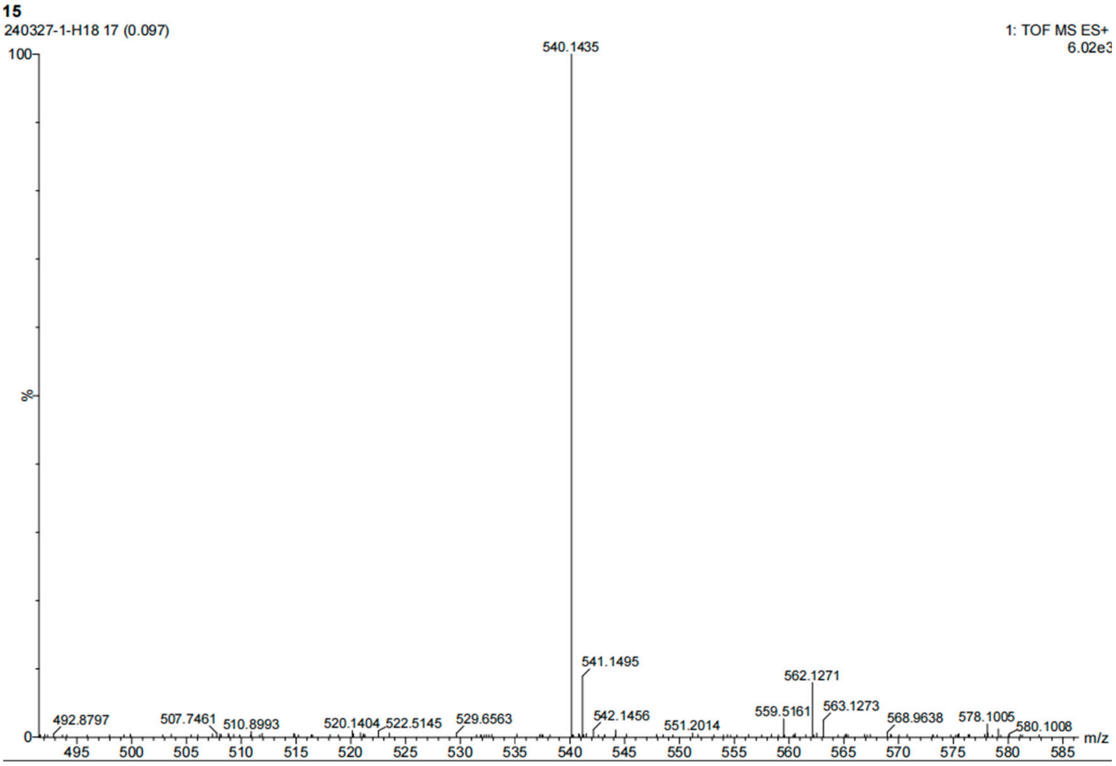

Figure S54 The HRMS spectrum of compound **7r**

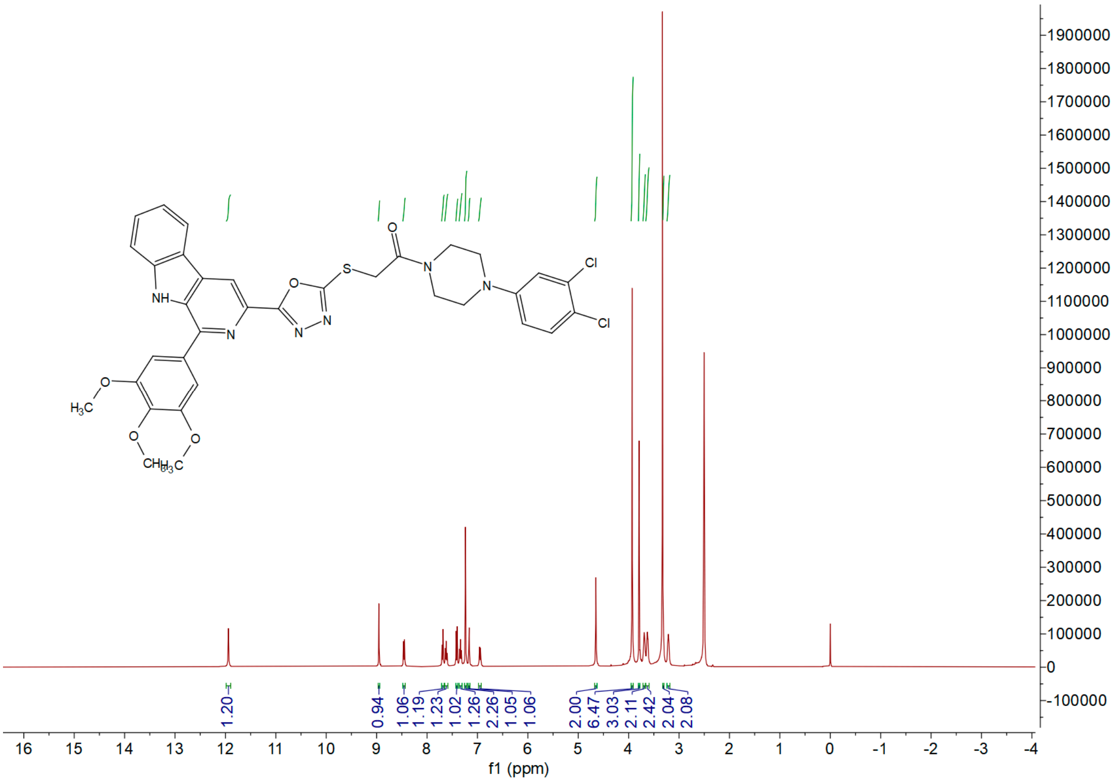

Figure S55 ^{1}H NMR Spectrum of compound **8a**

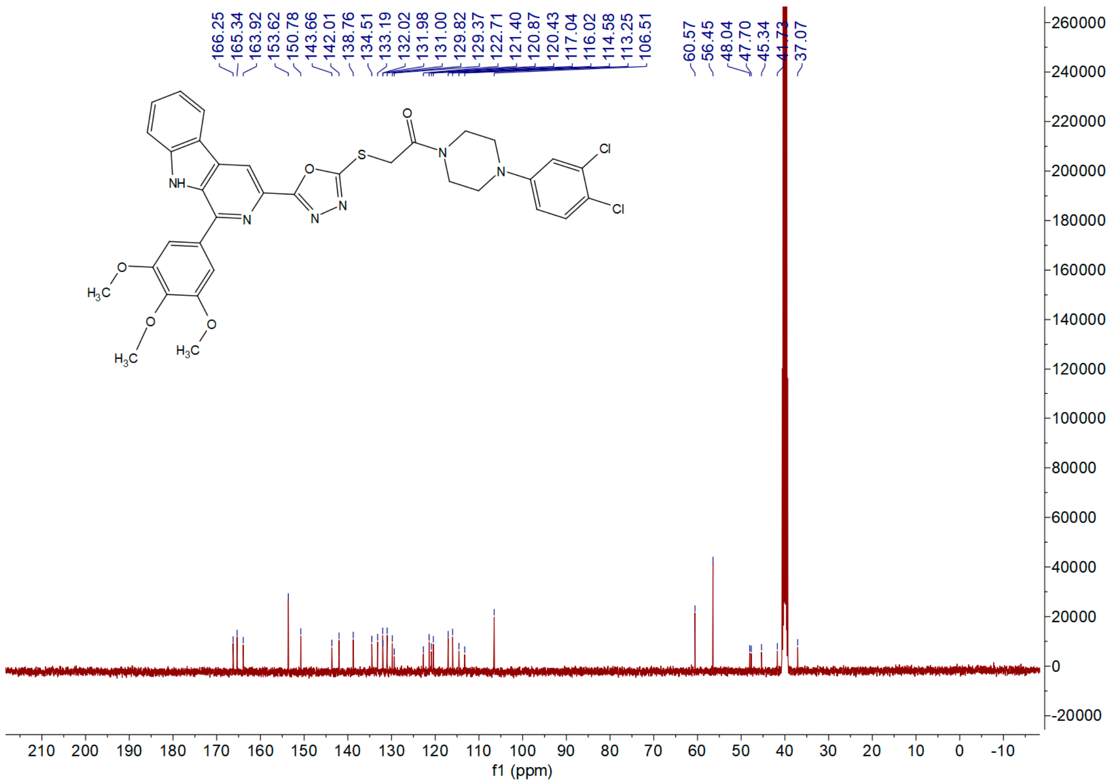

Figure S56 The ^{13}C NMR Spectrum of compound **8a**

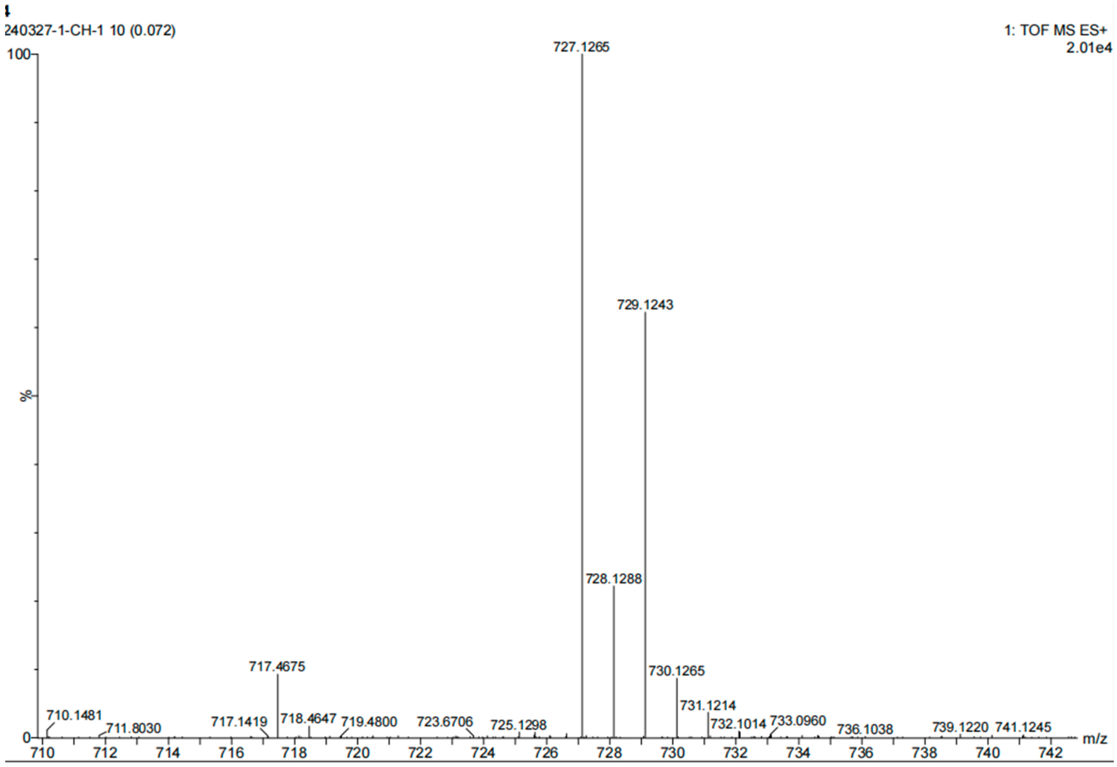

Figure S57 The HRMS spectrum of compound **8a**

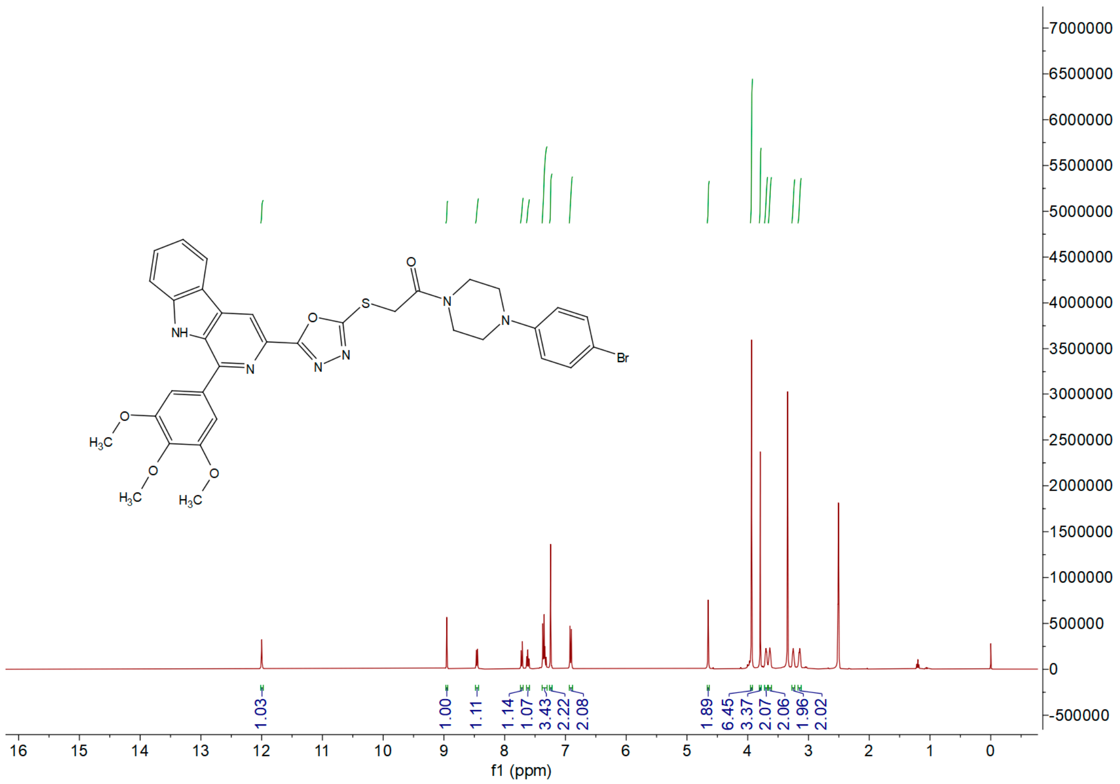

Figure S58 The ^{1}H NMR Spectrum of compound **8b**

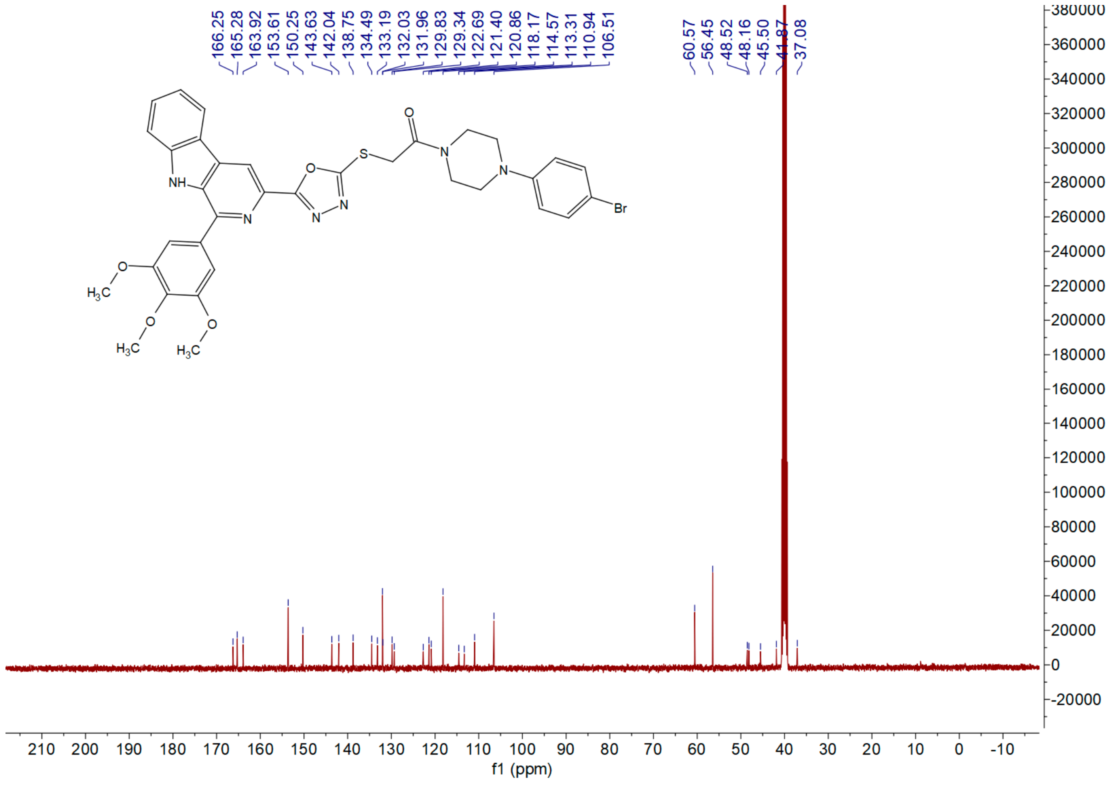

Figure S59 The ^{13}C NMR Spectrum of compound **8b**

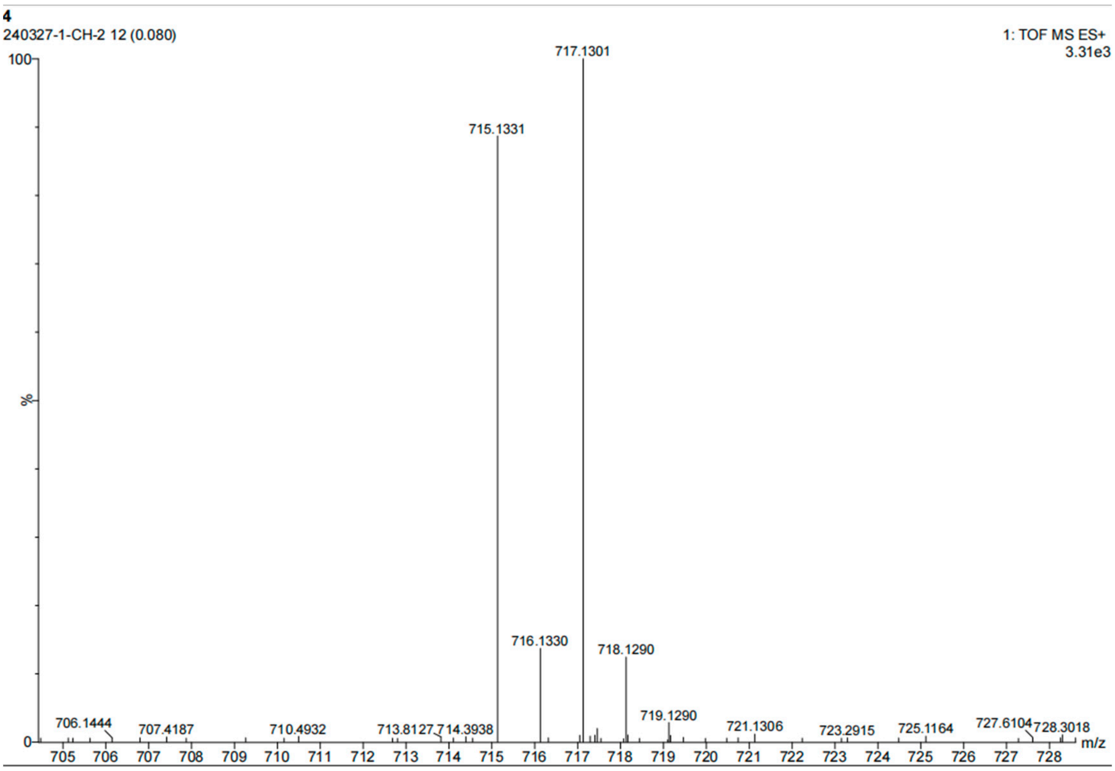

Figure S60 The HRMS spectrum of compound **8b**

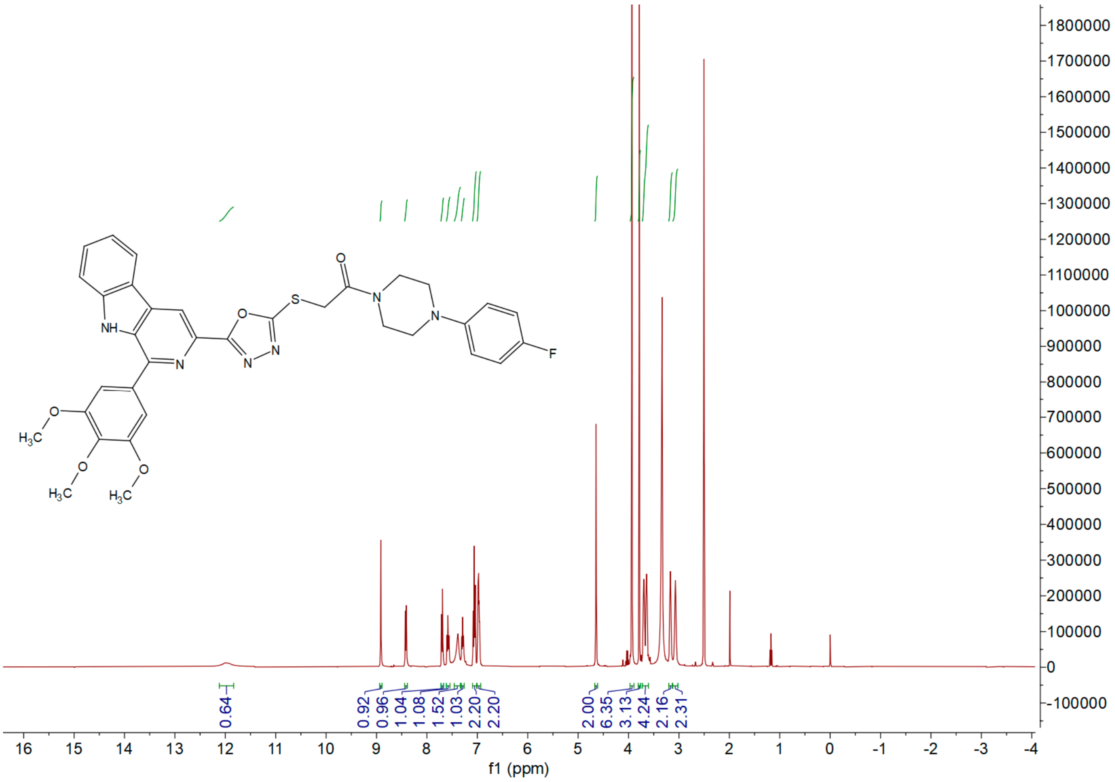

Figure S61 The ^{1}H NMR Spectrum of compound **8d**

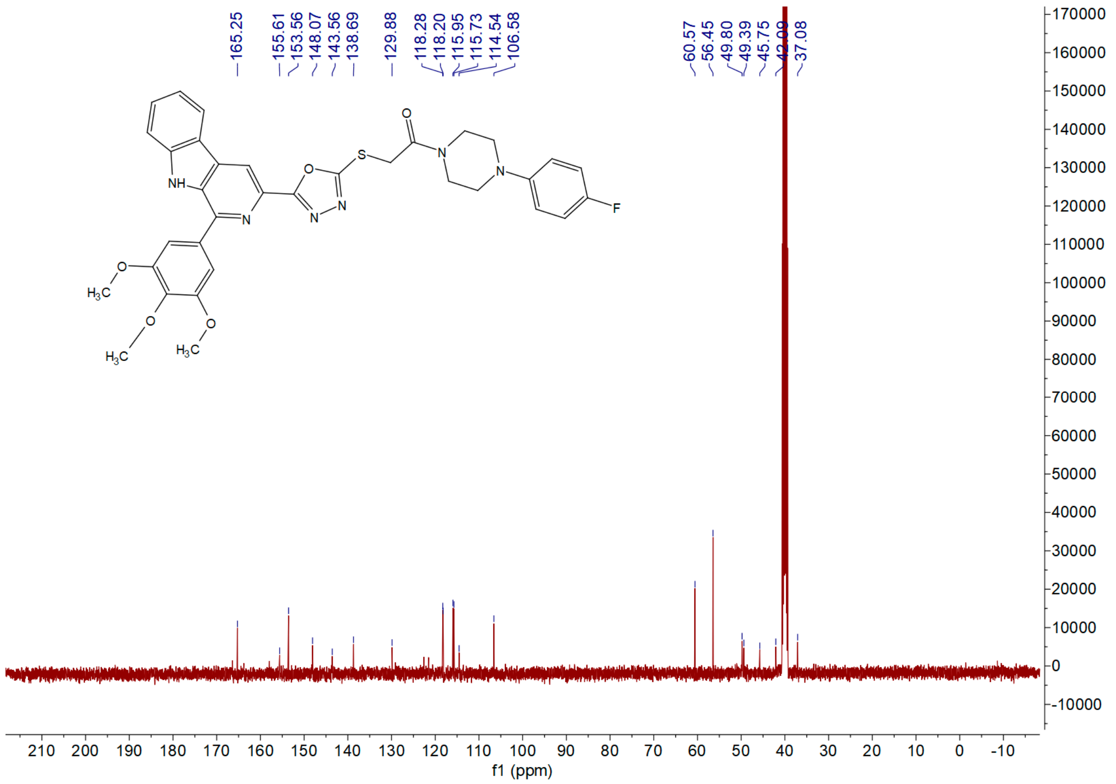

Figure S62 The ^{13}C NMR Spectrum of compound **8d**

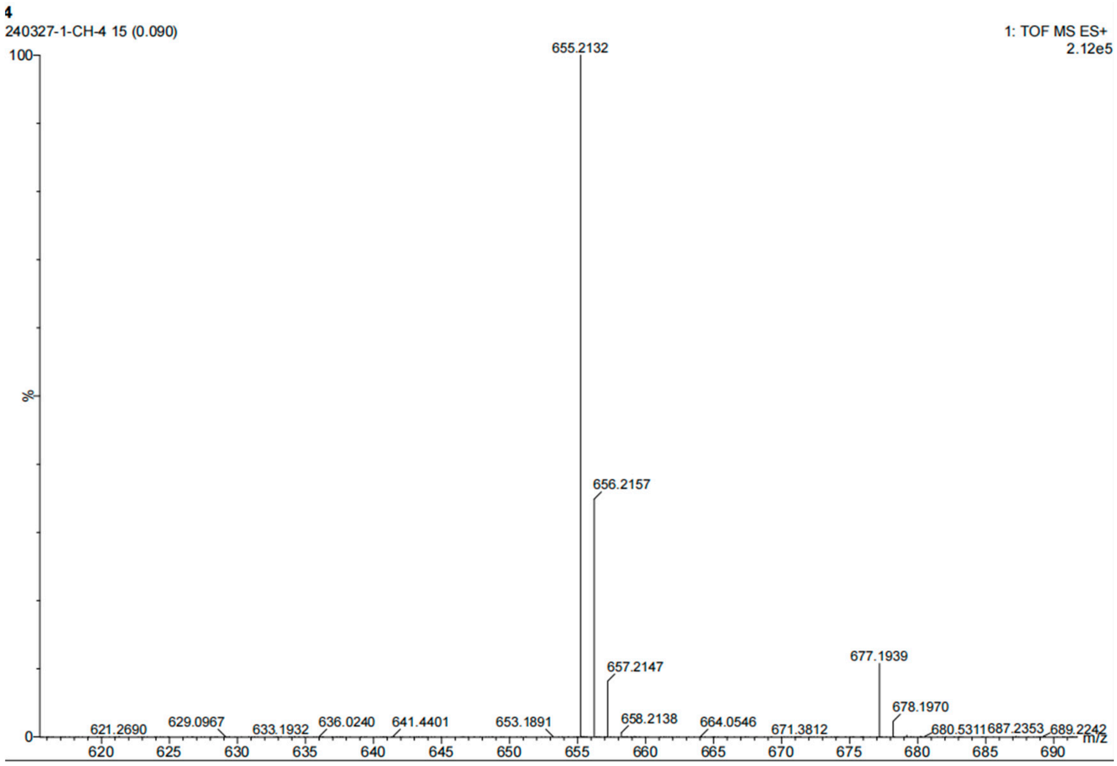

Figure S63 The HRMS spectrum of compound **8d**

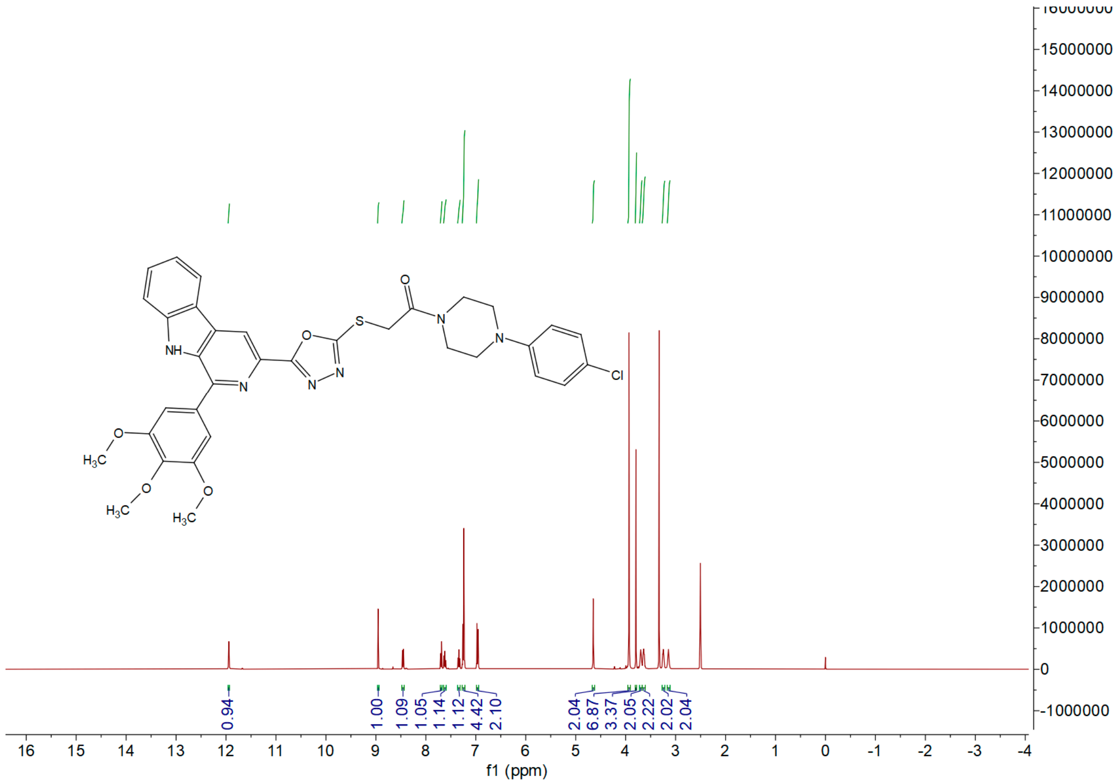

Figure S64 The ^{1}H NMR Spectrum of compound **8e**

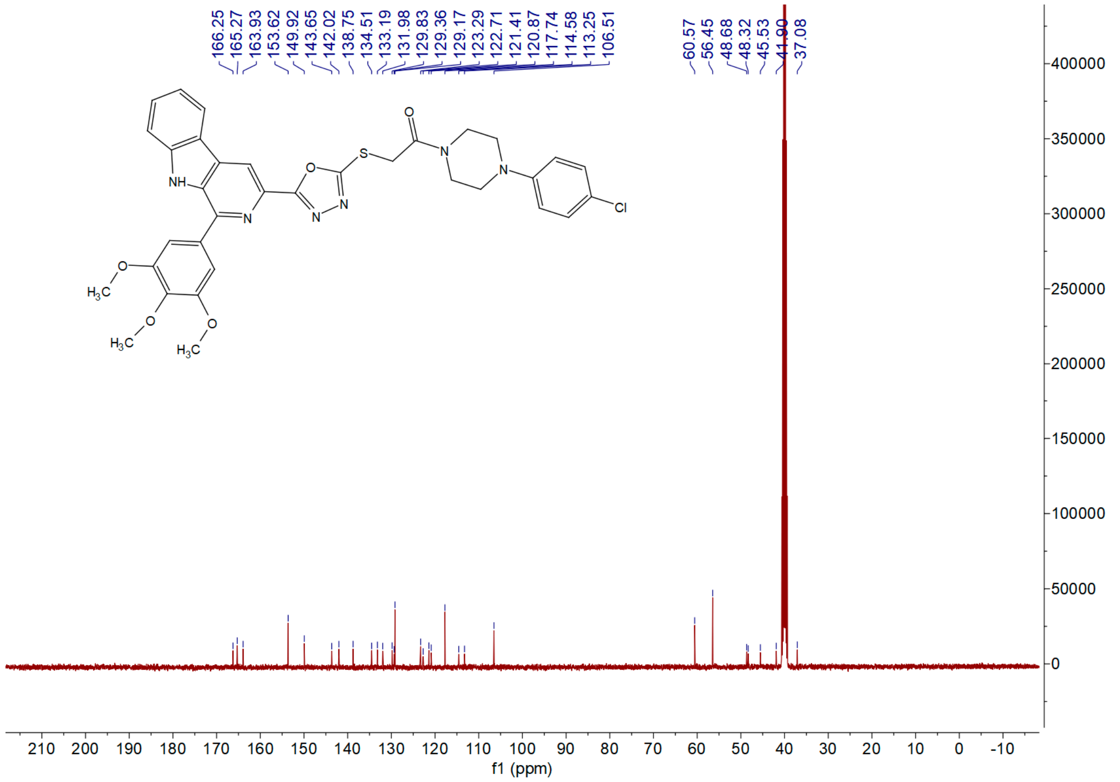

Figure S65 The ^{13}C NMR Spectrum of compound **8e**

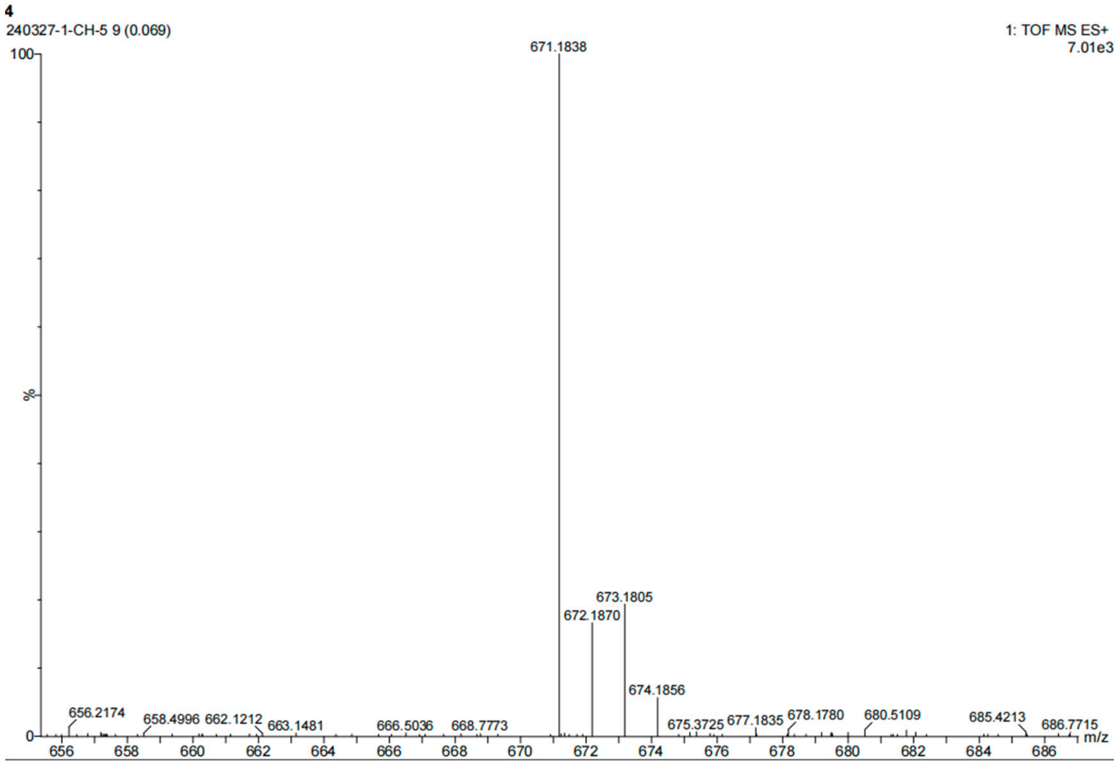

Figure S66 The HRMS spectrum of compound **8e**

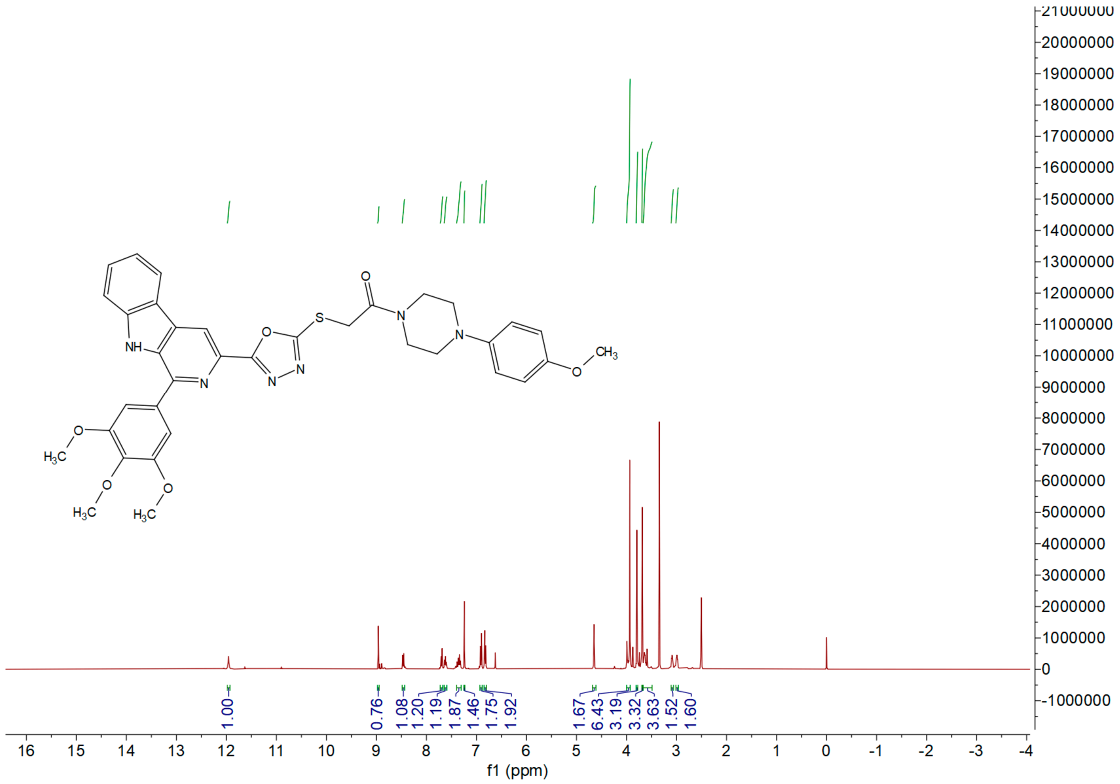

Figure S67 The ^{1}H NMR Spectrum of compound **8f**

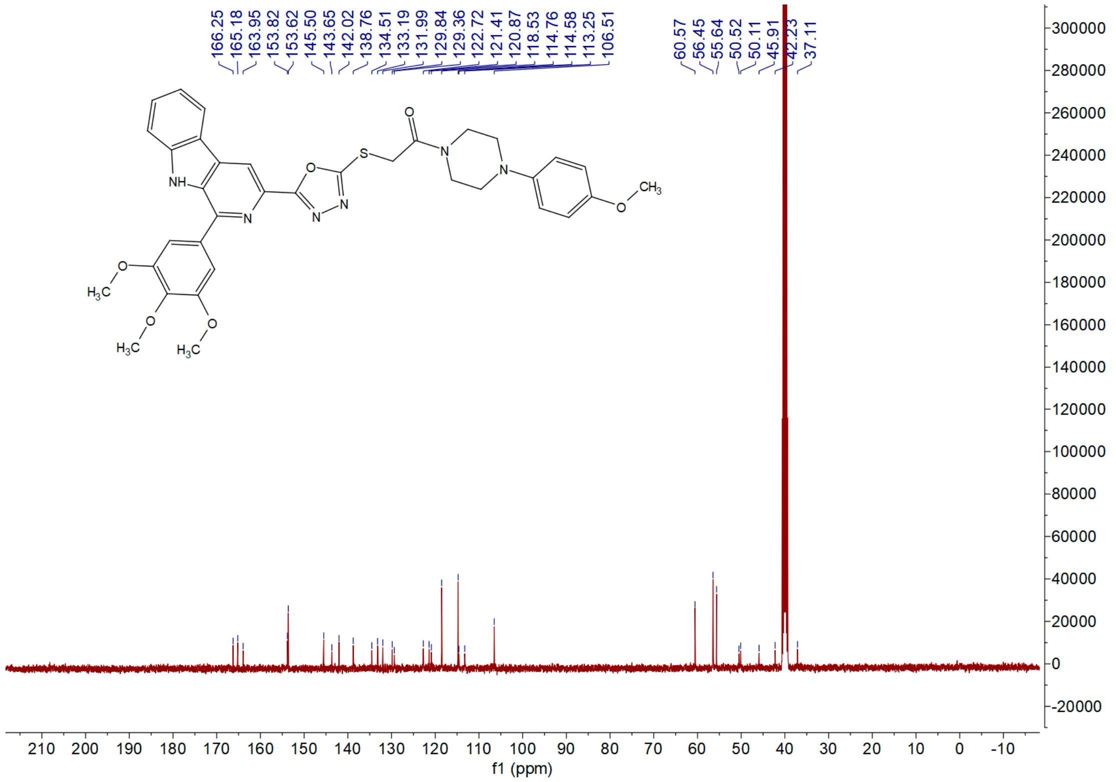

Figure S68 The ^{13}C NMR Spectrum of compound **8f**

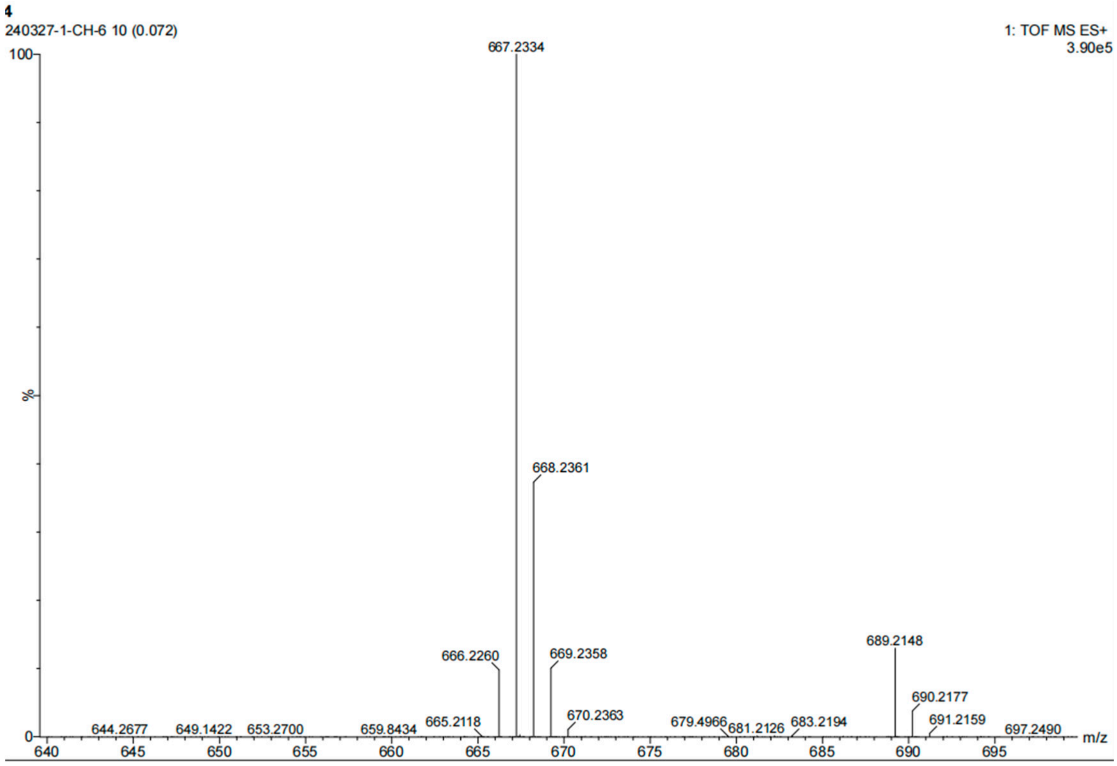

Figure S69 The HRMS spectrum of compound **8f**

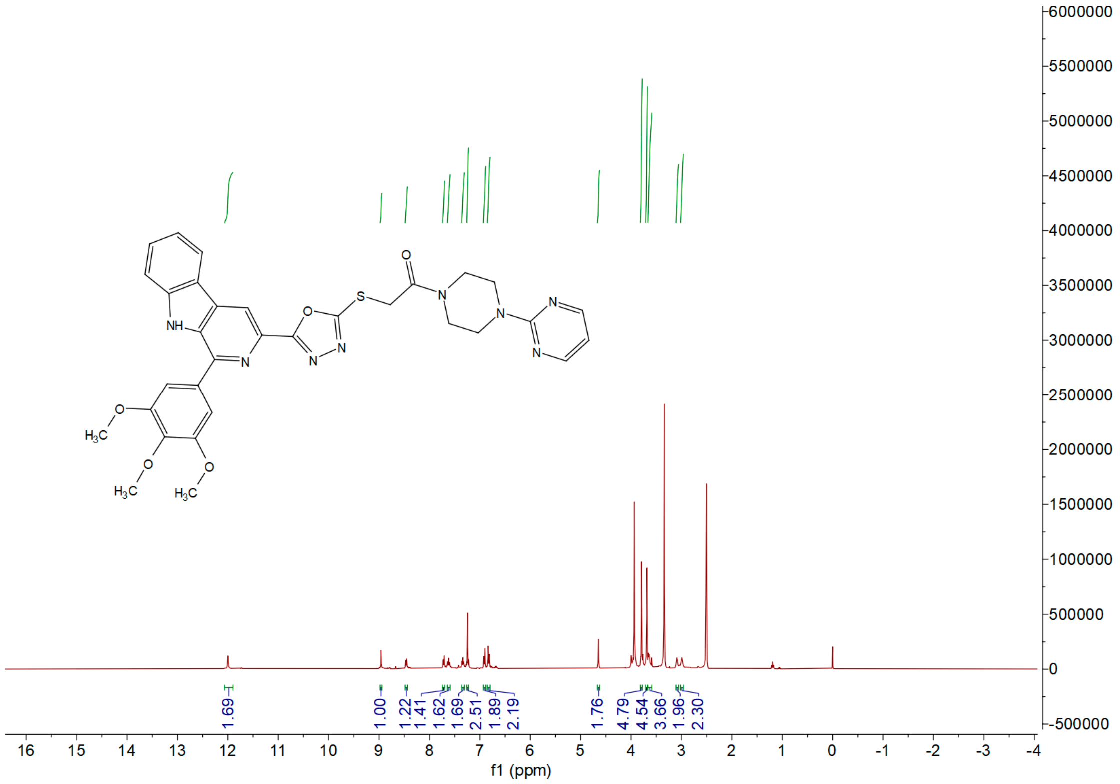

Figure S70 The ^{1}H NMR Spectrum of compound **8g**

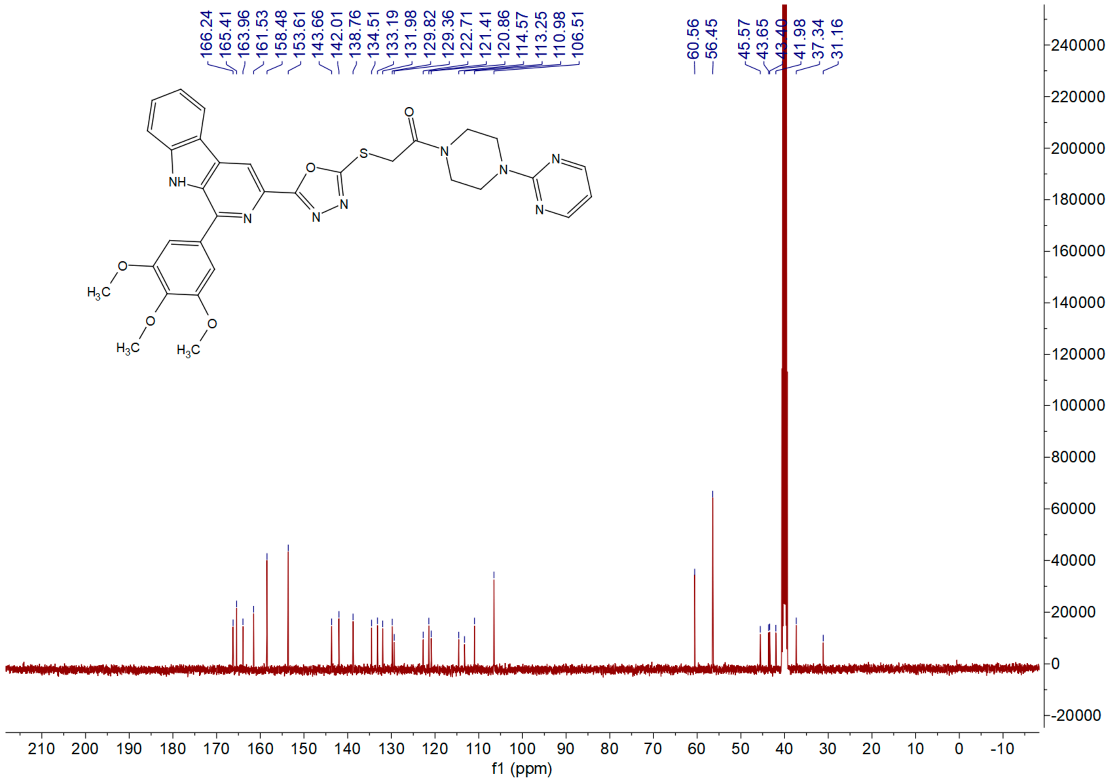

Figure S71 The ^{13}C NMR Spectrum of compound **8g**

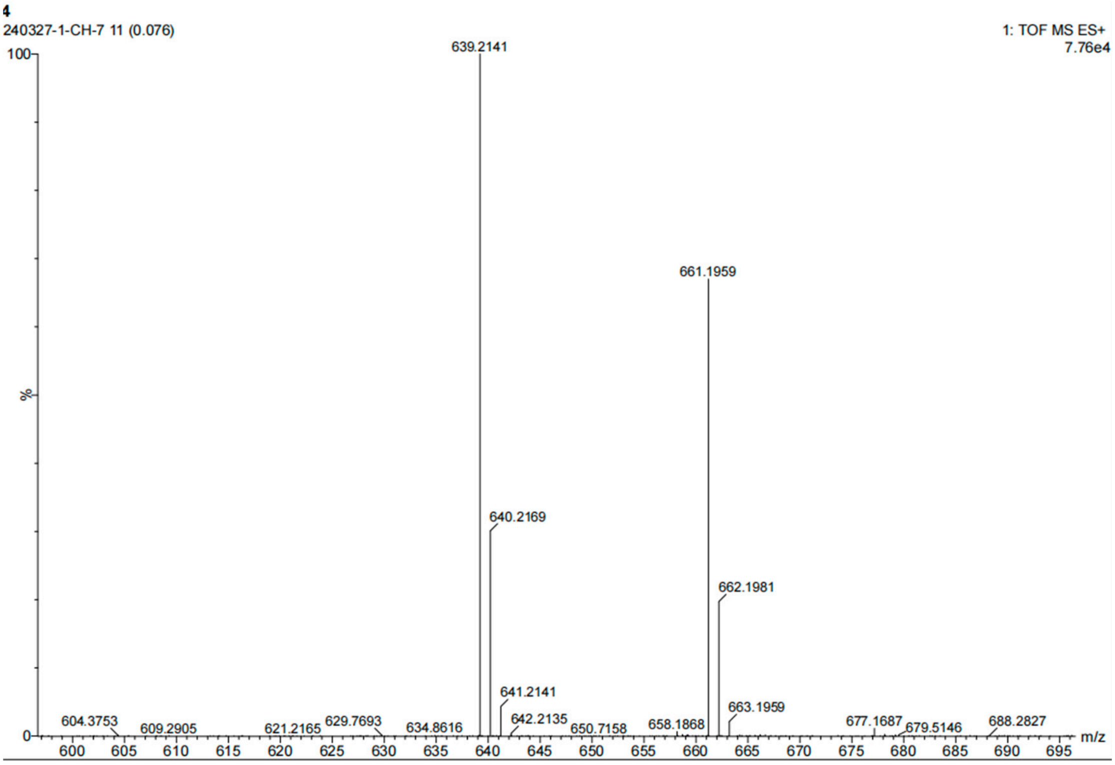

Figure S72 The HRMS spectrum of compound **8g**

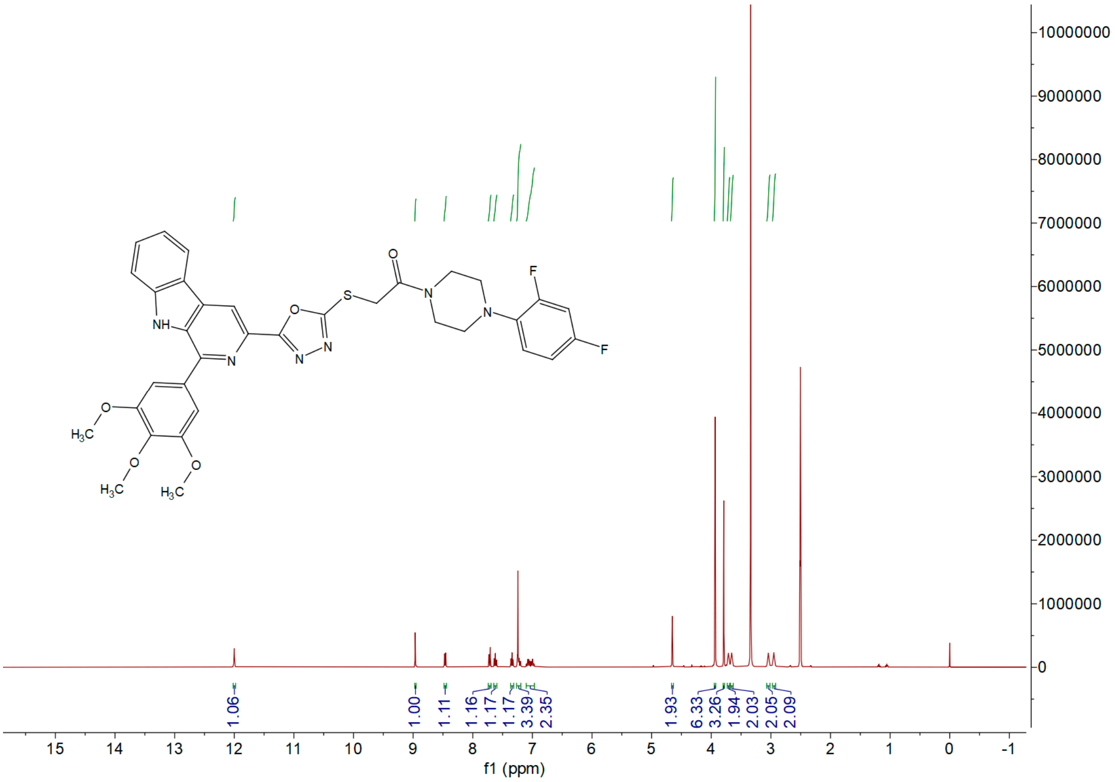

Figure S73 The ^{1}H NMR Spectrum of compound **8h**

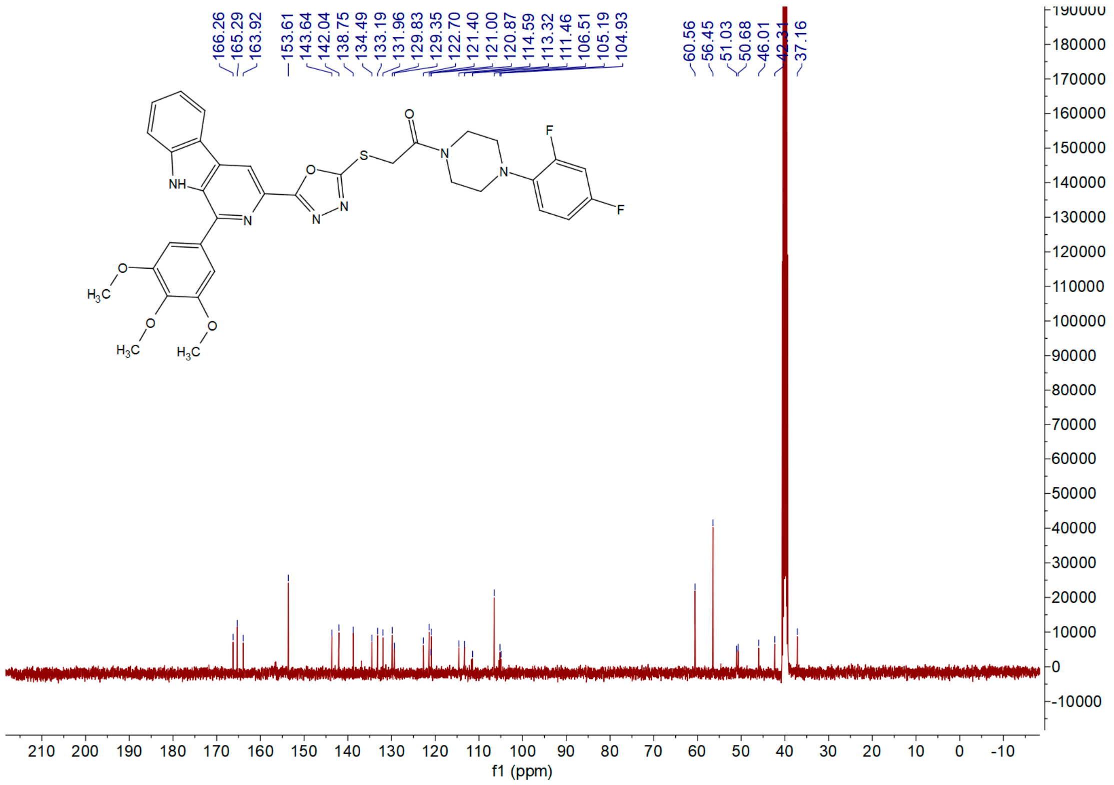

Figure S74 The ^{13}C NMR Spectrum of compound **8h**

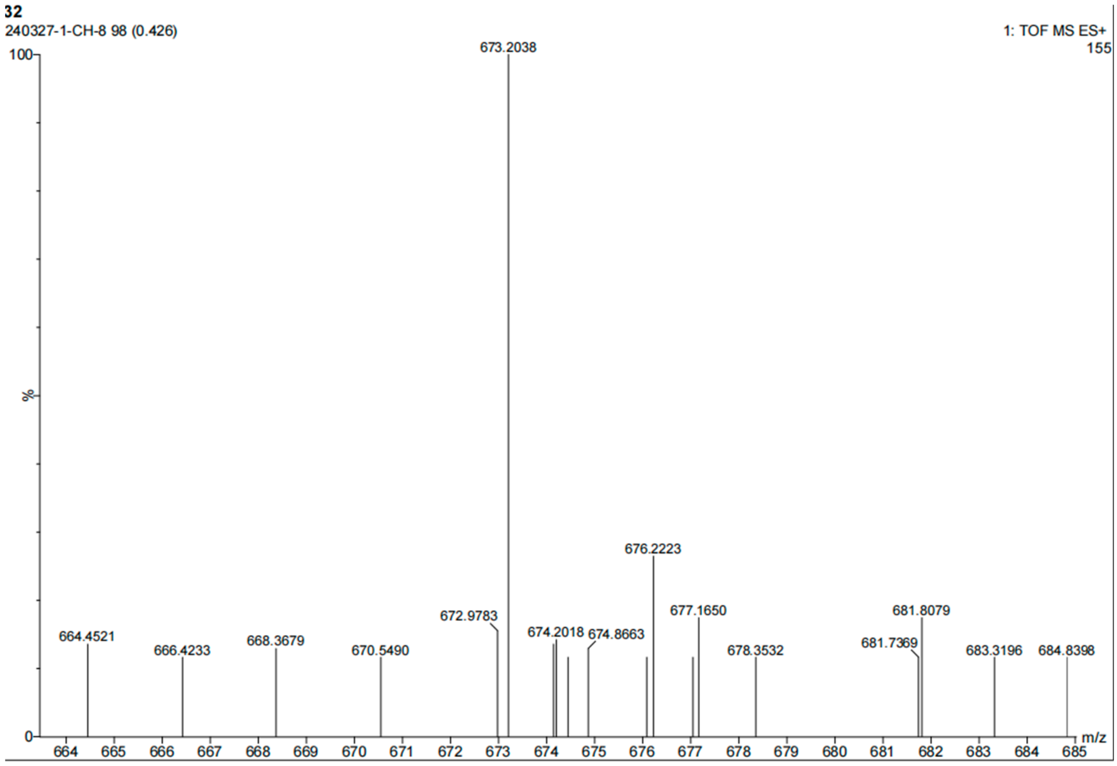

Figure S75 The HRMS spectrum of compound **8h**

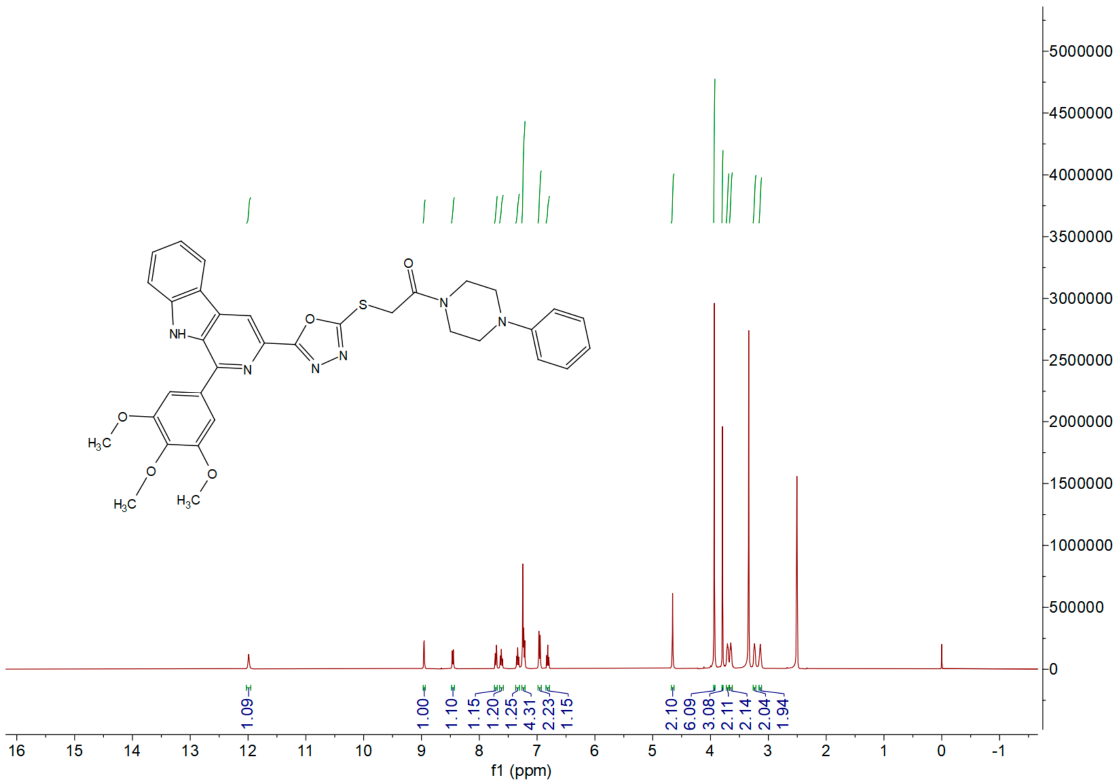

Figure S76 The ^{1}H NMR Spectrum of compound **8i**

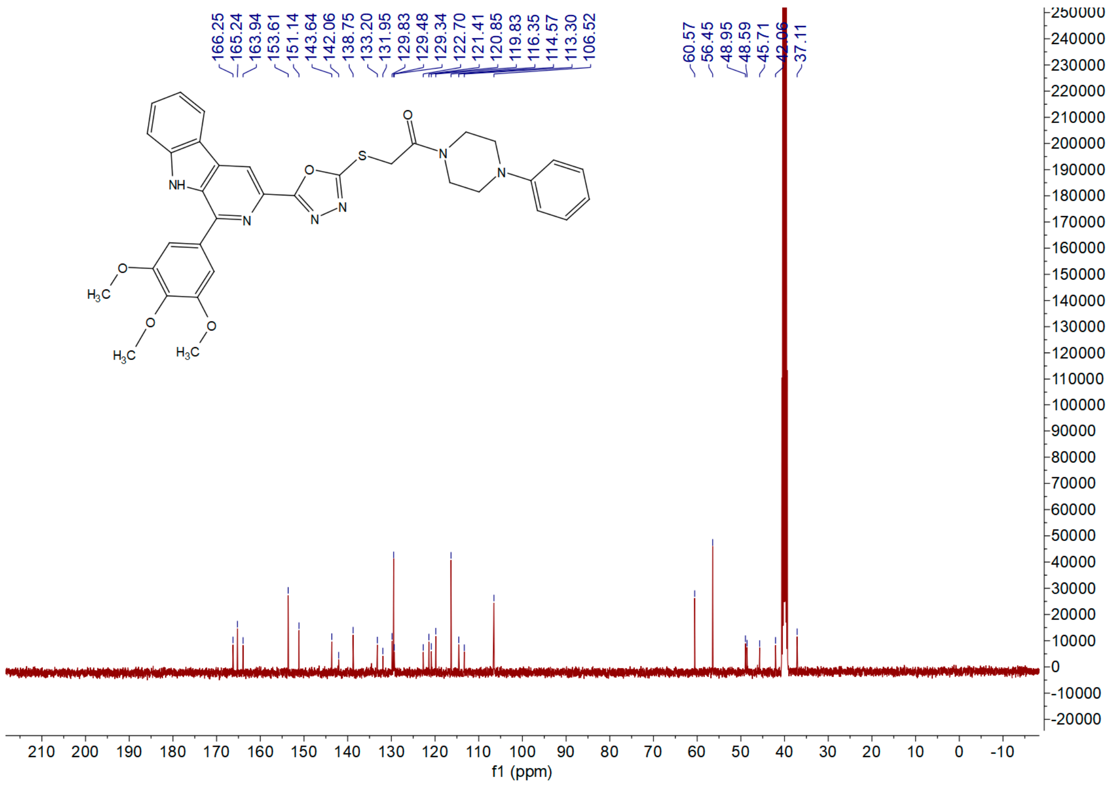

Figure S77 The ^{13}C NMR Spectrum of compound **8i**

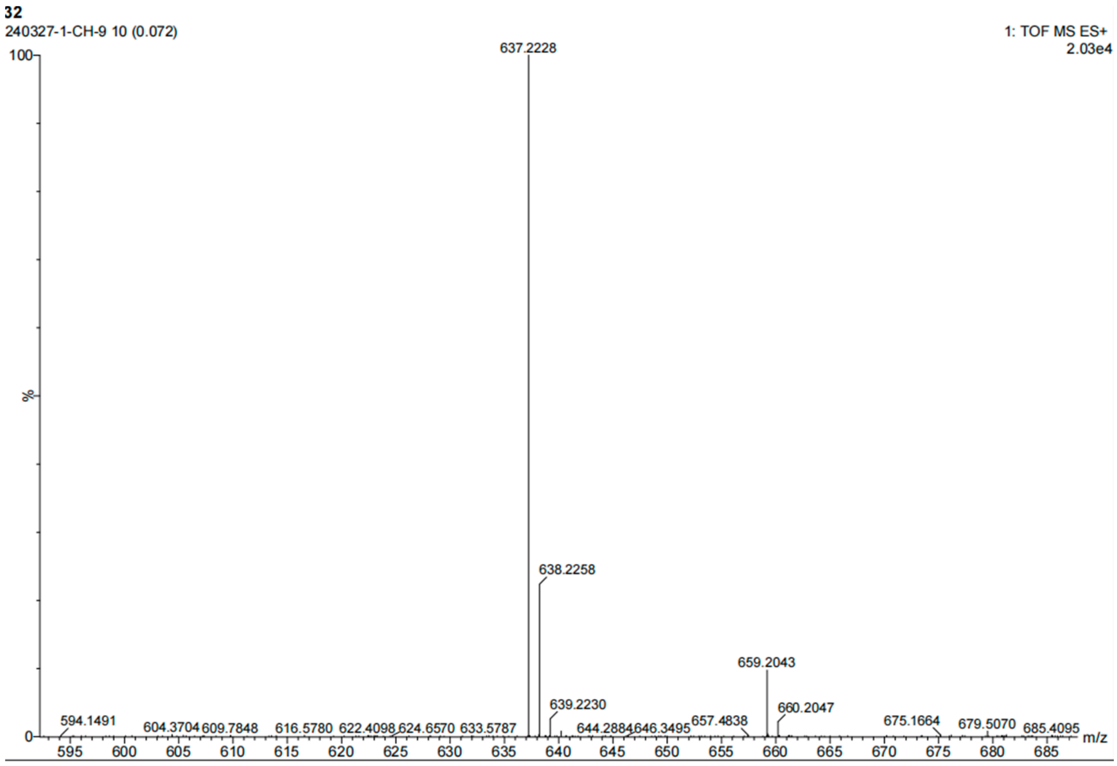

Figure S78 The HRMS spectrum of compound **8i**

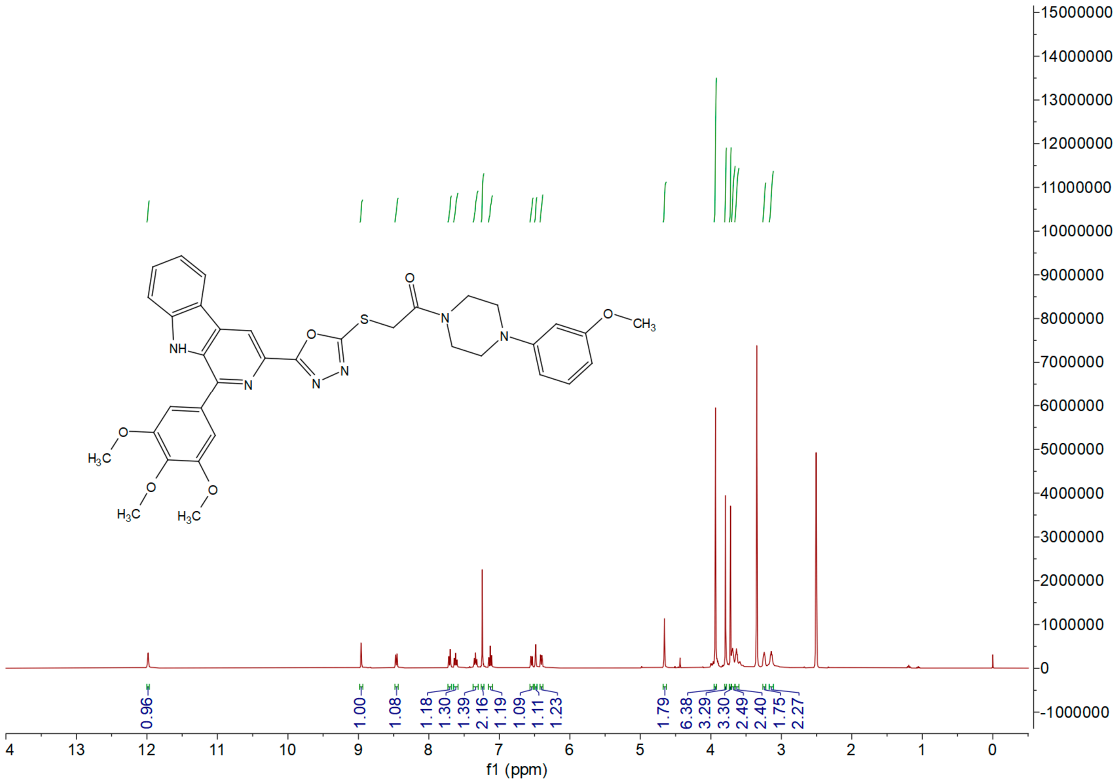

Figure S79 The ^{1}H NMR Spectrum of compound **8J**

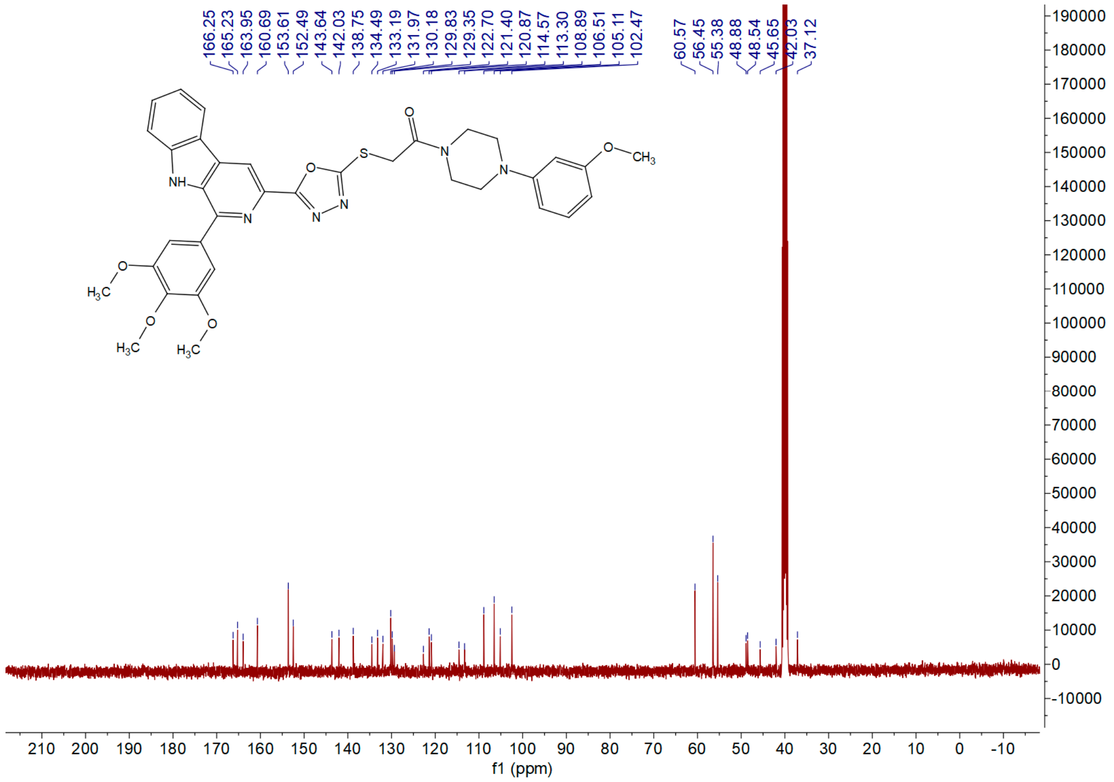

Figure S80 The ^{13}C NMR Spectrum of compound **8J**

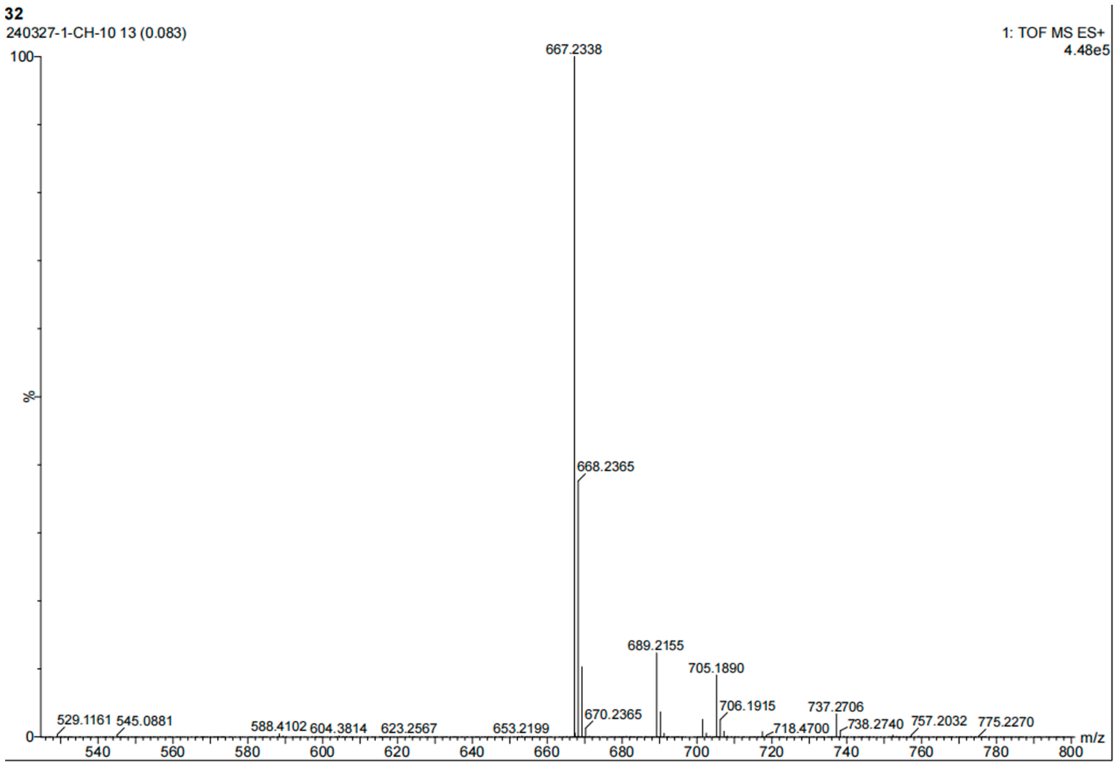

Figure S81 The HRMS spectrum of compound **8J**

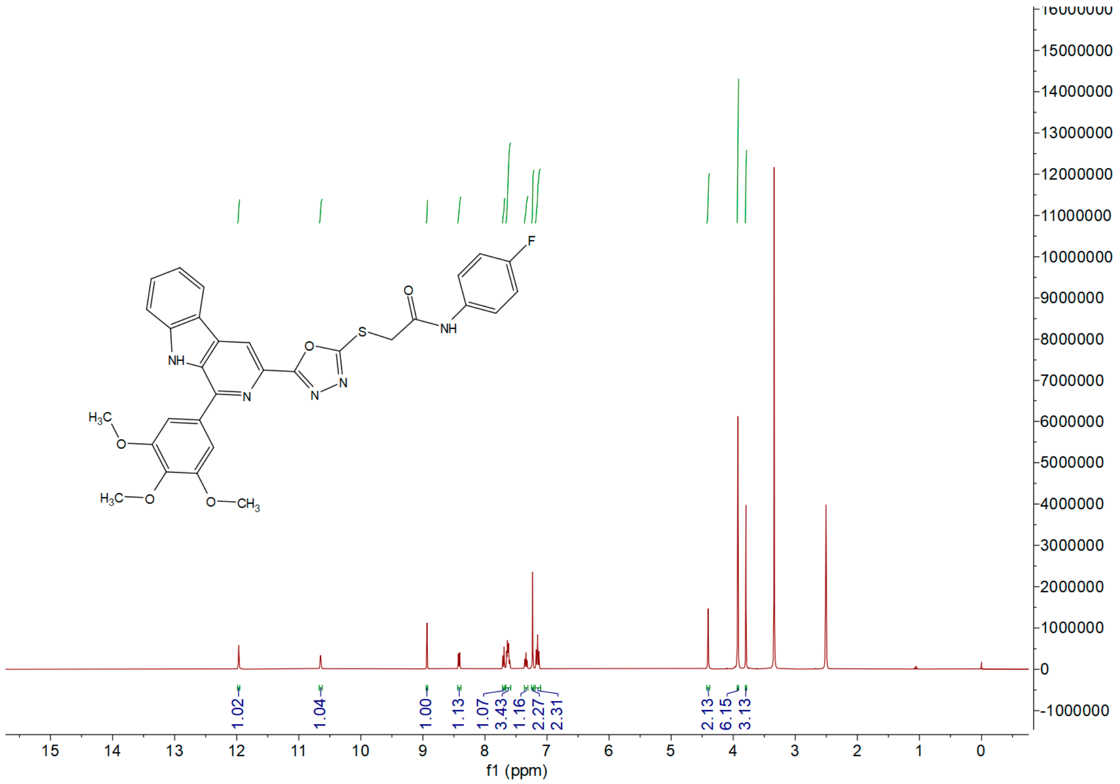

Figure S82 The ^{1}H NMR Spectrum of compound **8k**

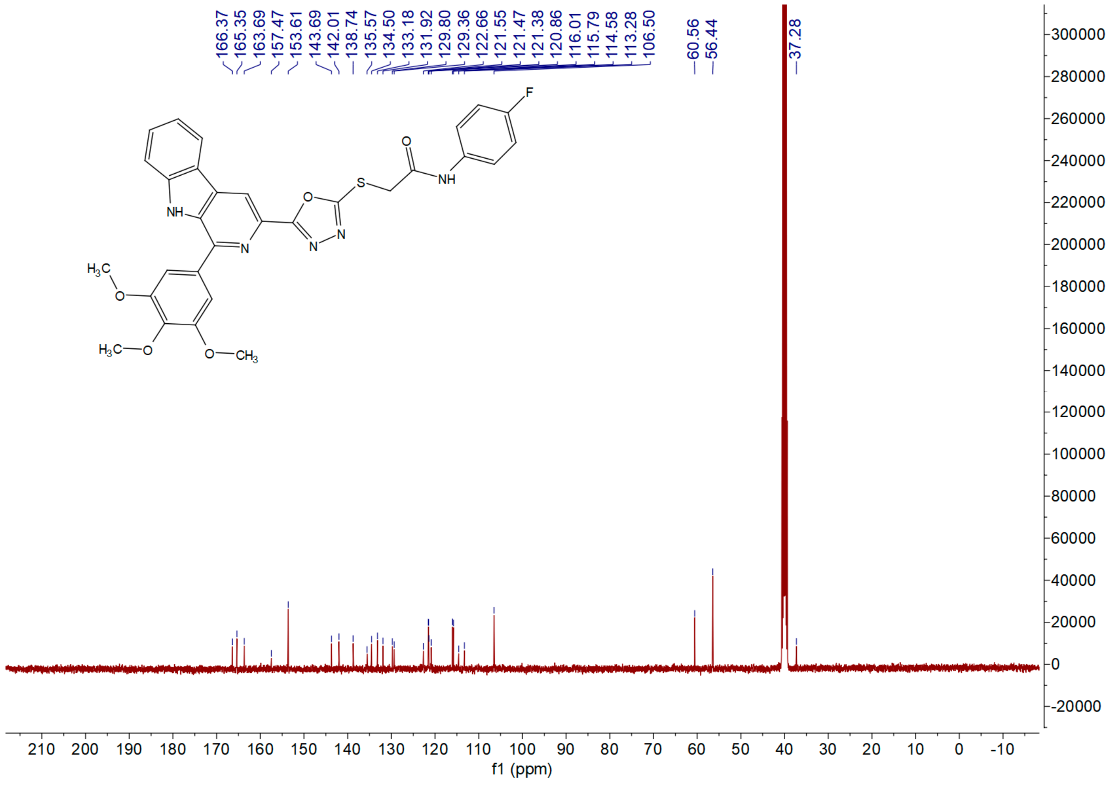

Figure S83 The ^{13}C NMR Spectrum of compound **8k**

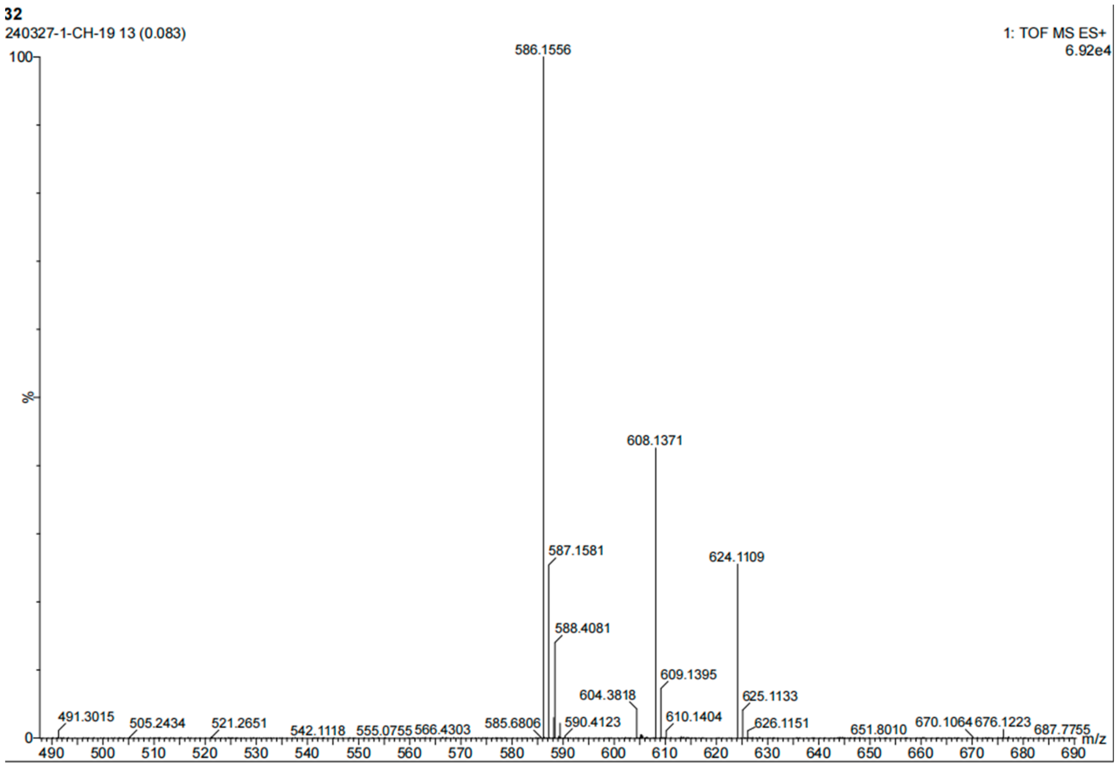

Figure S84 The HRMS spectrum of compound **8k**

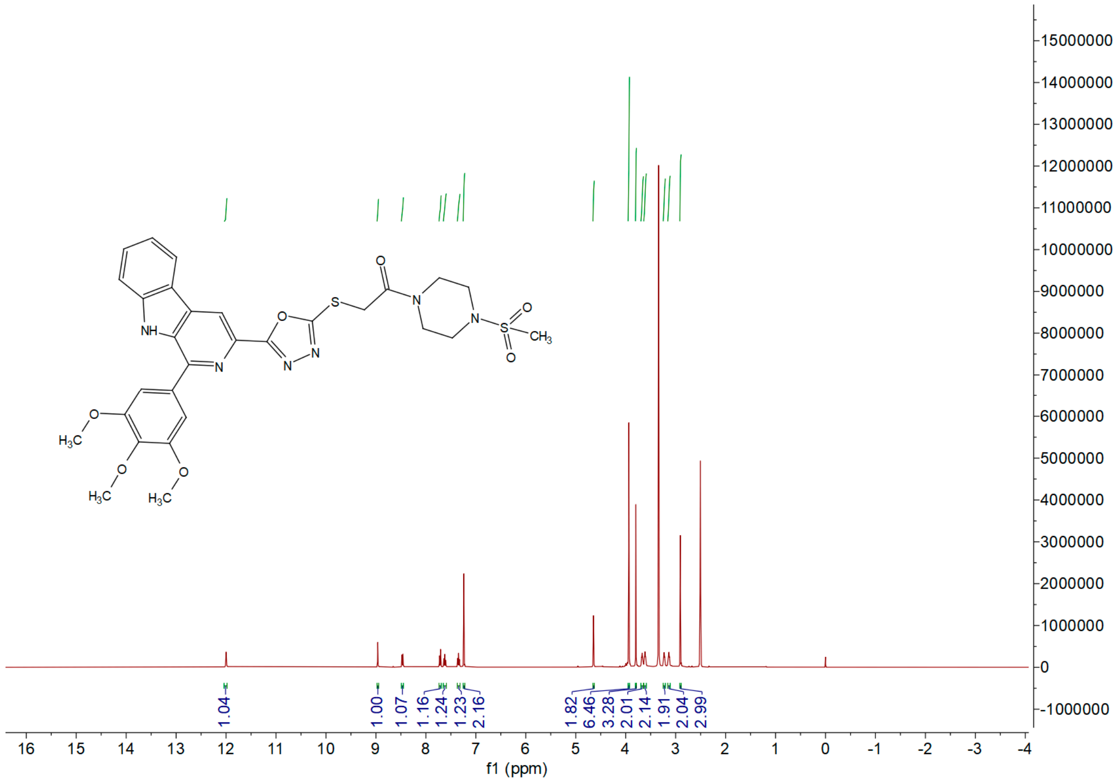

Figure S85 The ^{1}H NMR Spectrum of compound **8m**

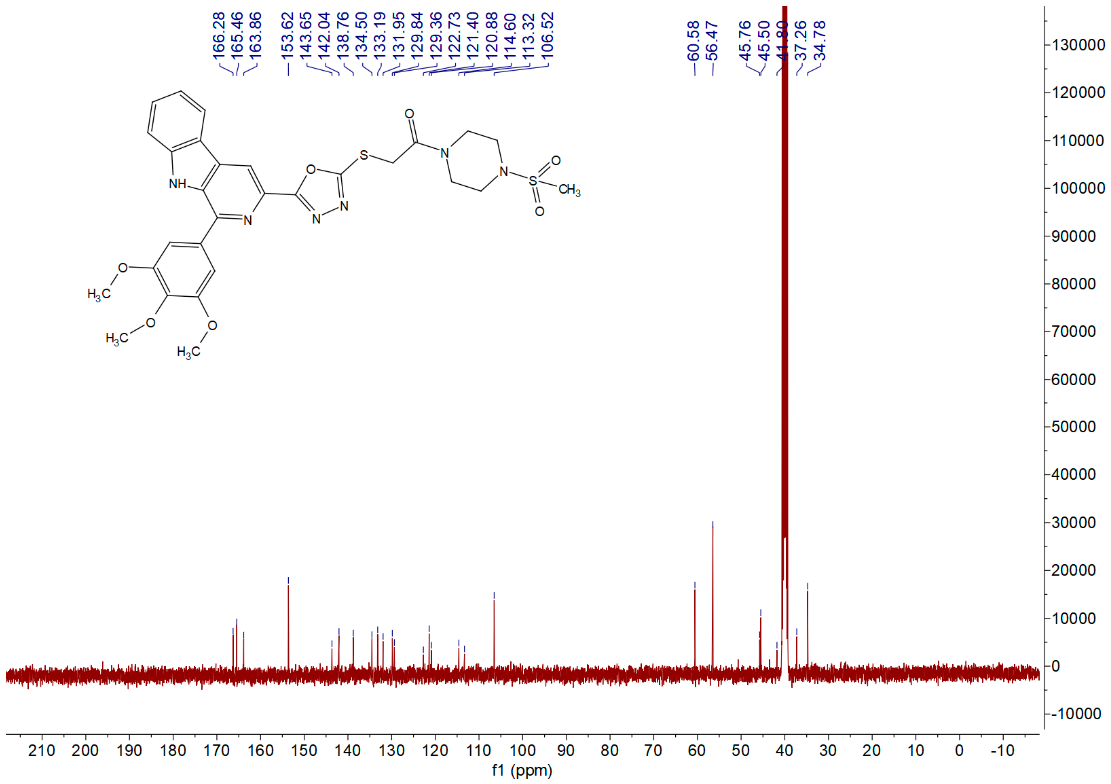

Figure S86 The ^{13}C NMR Spectrum of compound **8m**

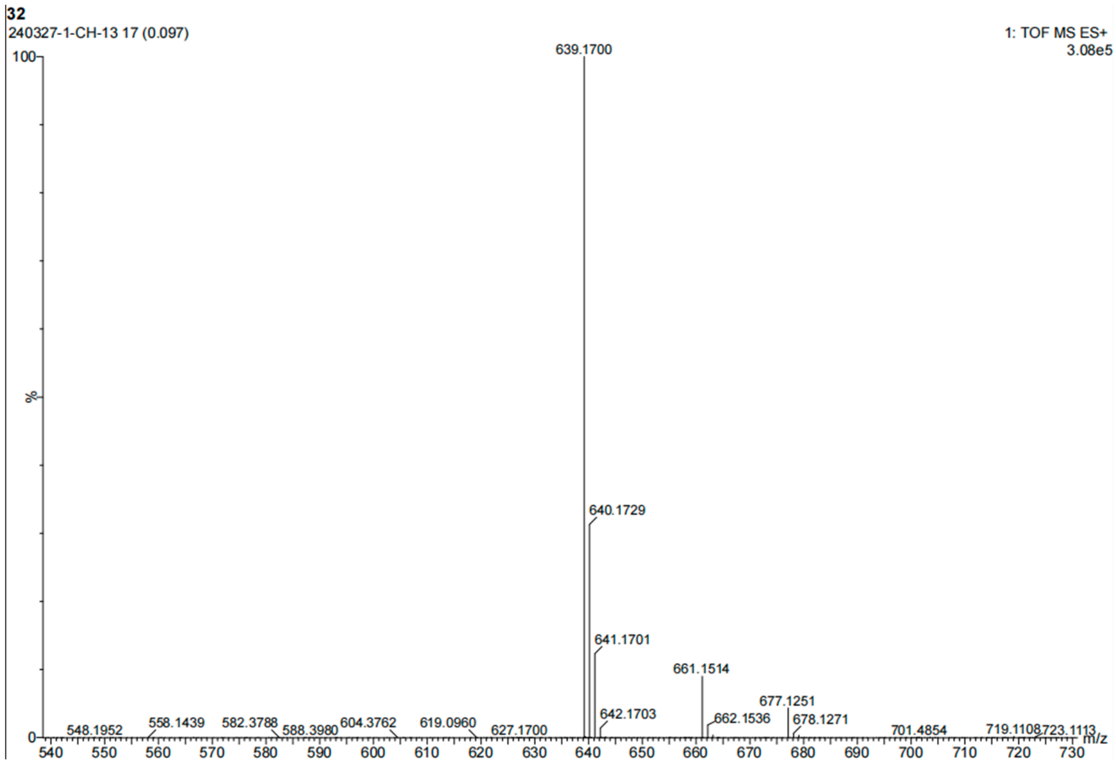

Figure S87 The HRMS spectrum of compound **8m**

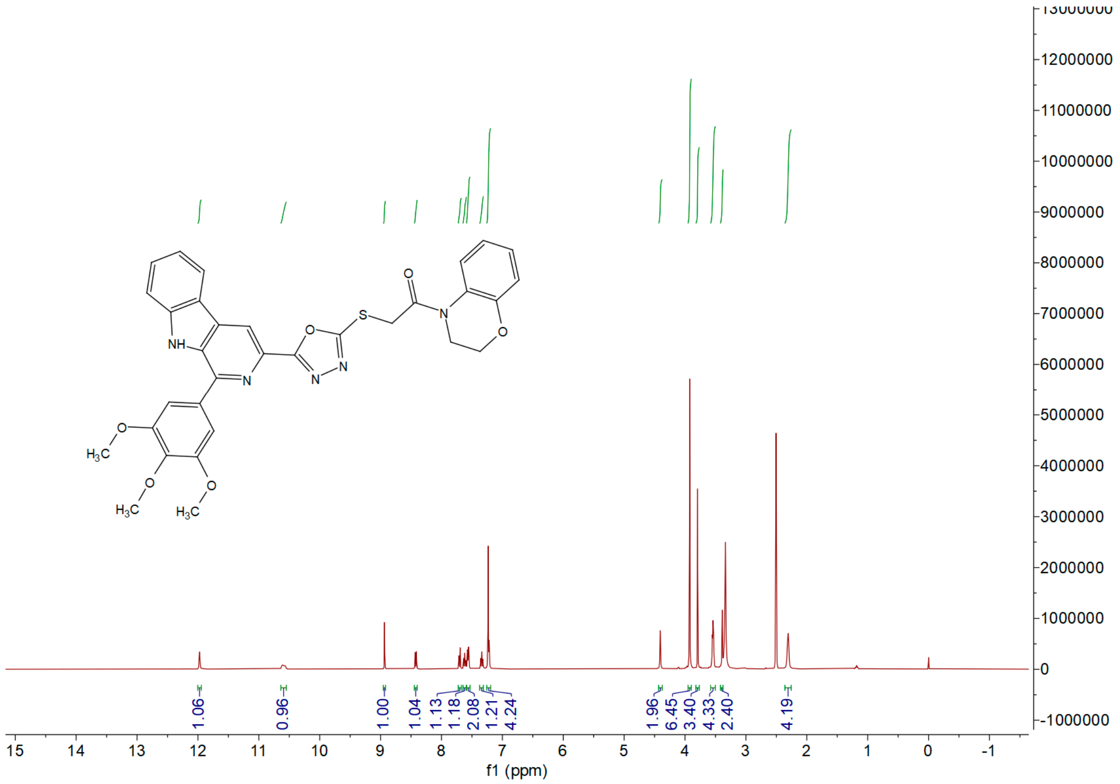

Figure S88 The ^{1}H NMR Spectrum of compound **8n**

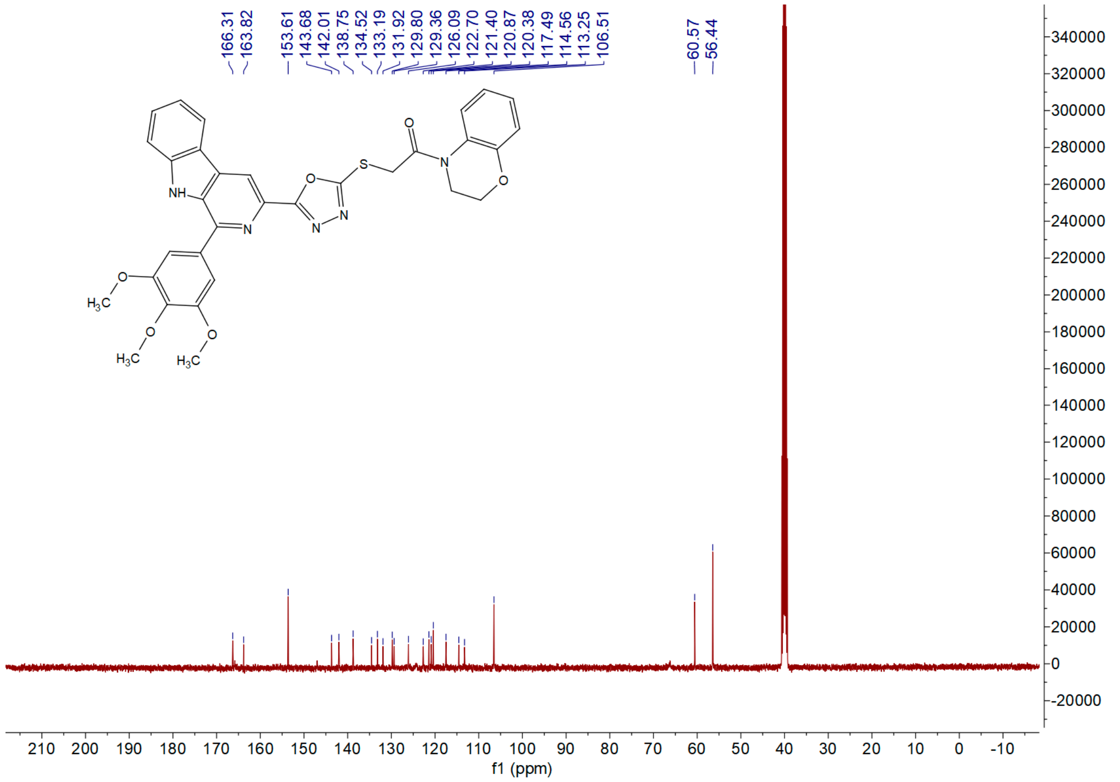

Figure S89 The ^{13}C NMR Spectrum of compound **8n**

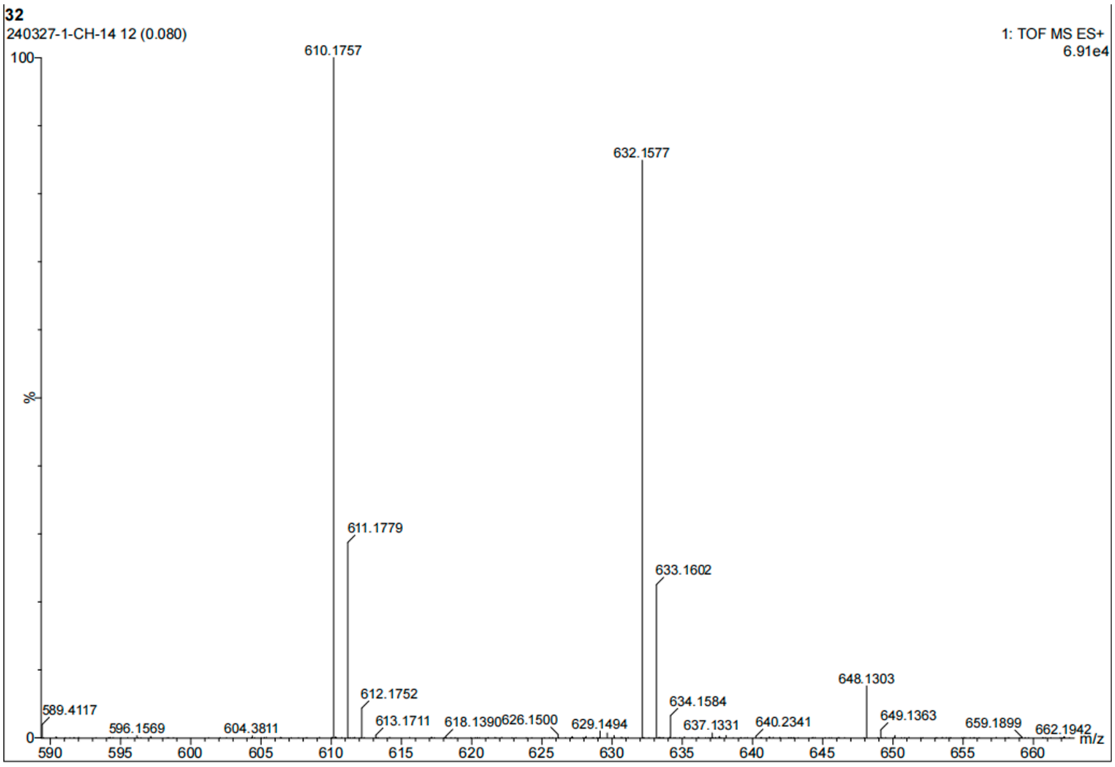

Figure S90 The HRMS spectrum of compound **8n**

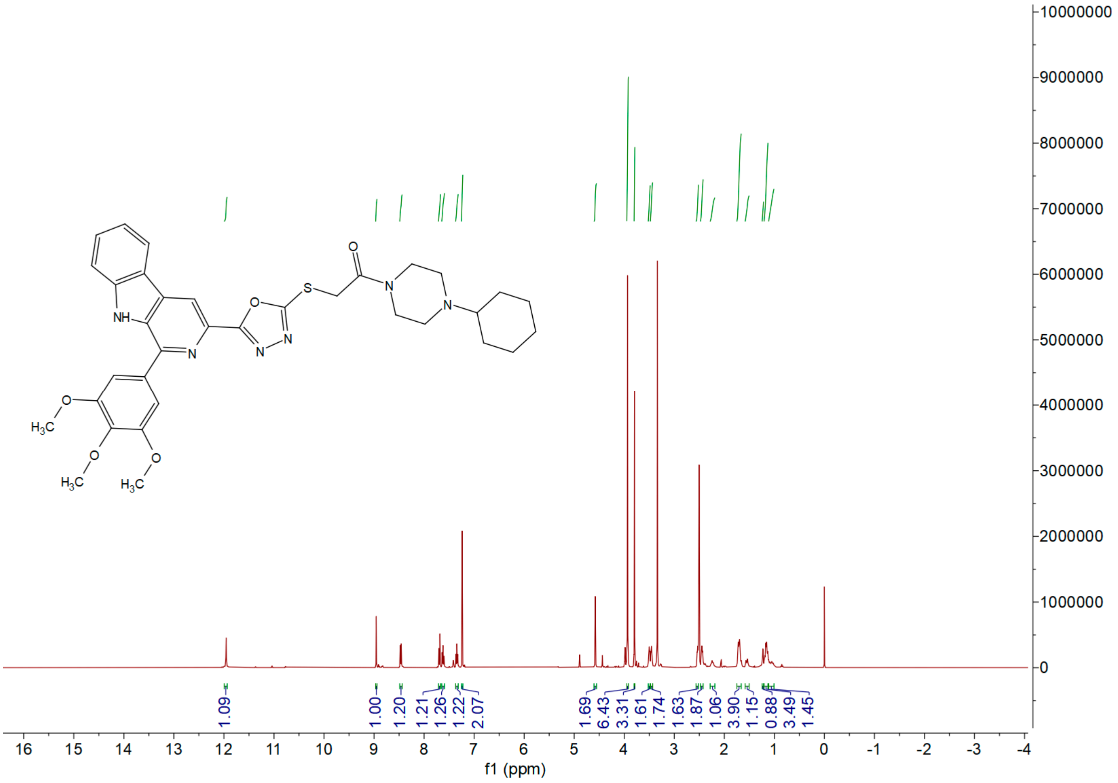

Figure S91 The ^{1}H NMR Spectrum of compound **8p**

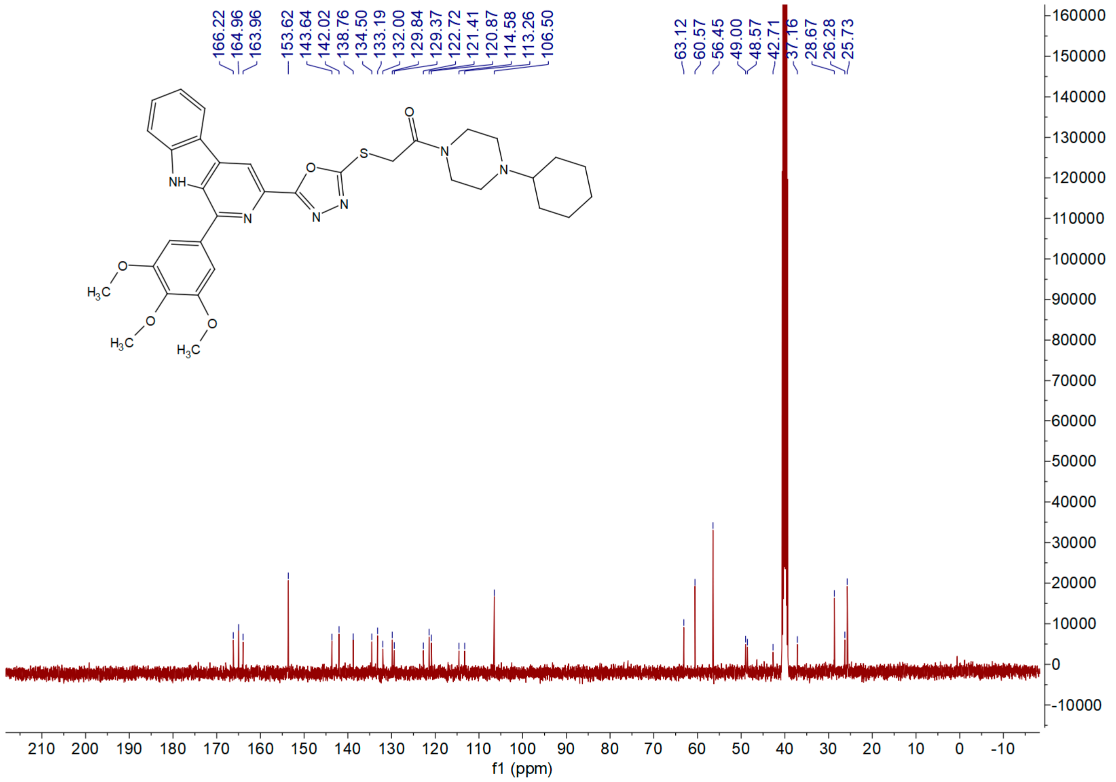

Figure S92 The ^{13}C NMR Spectrum of compound **8p**

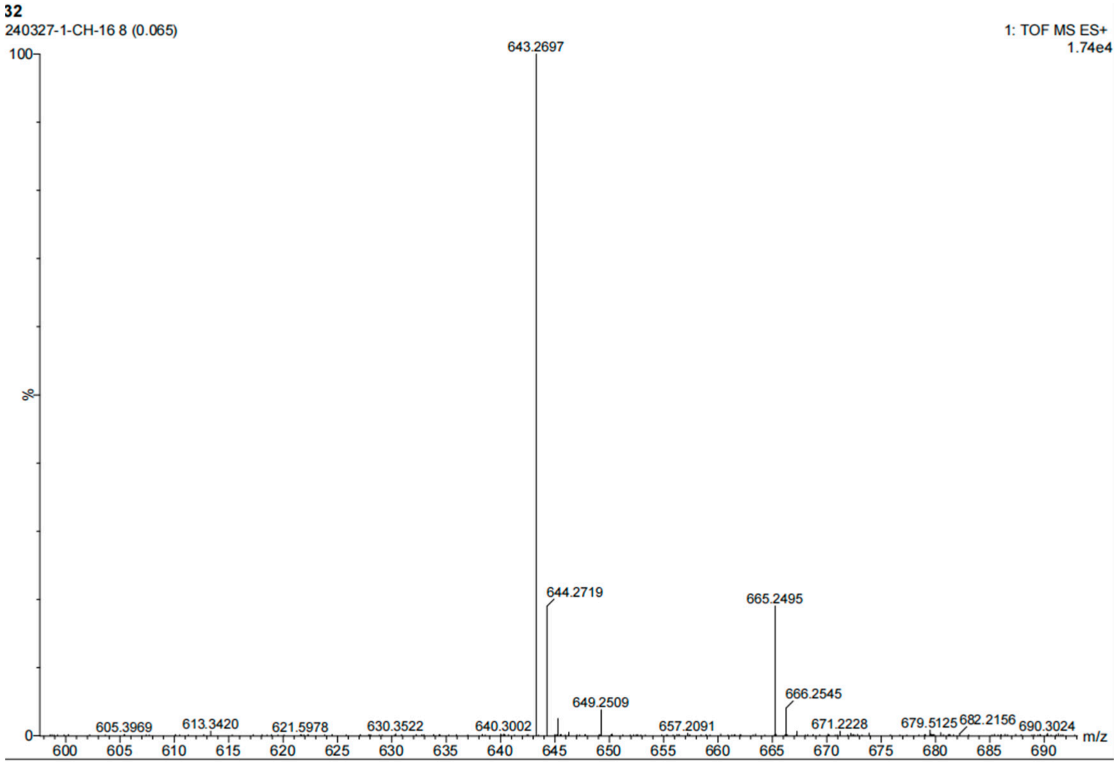

Figure S93 The HRMS spectrum of compound **8p**

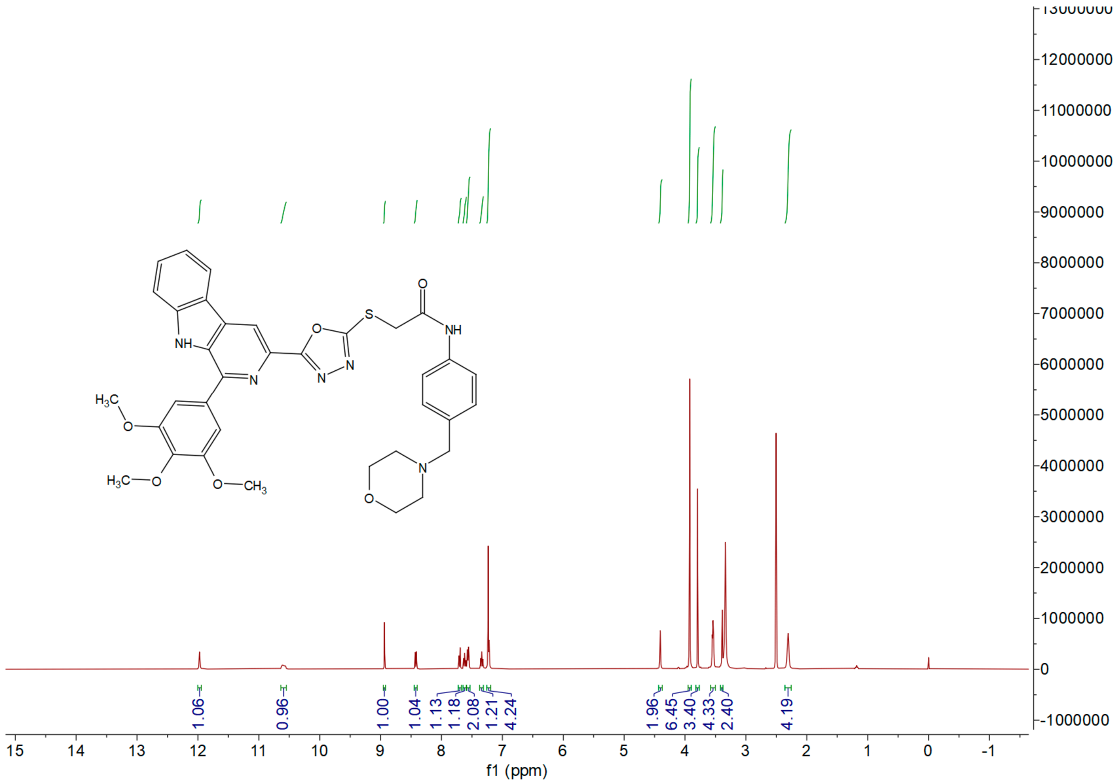

Figure S94 The ^{1}H NMR Spectrum of compound **8q**

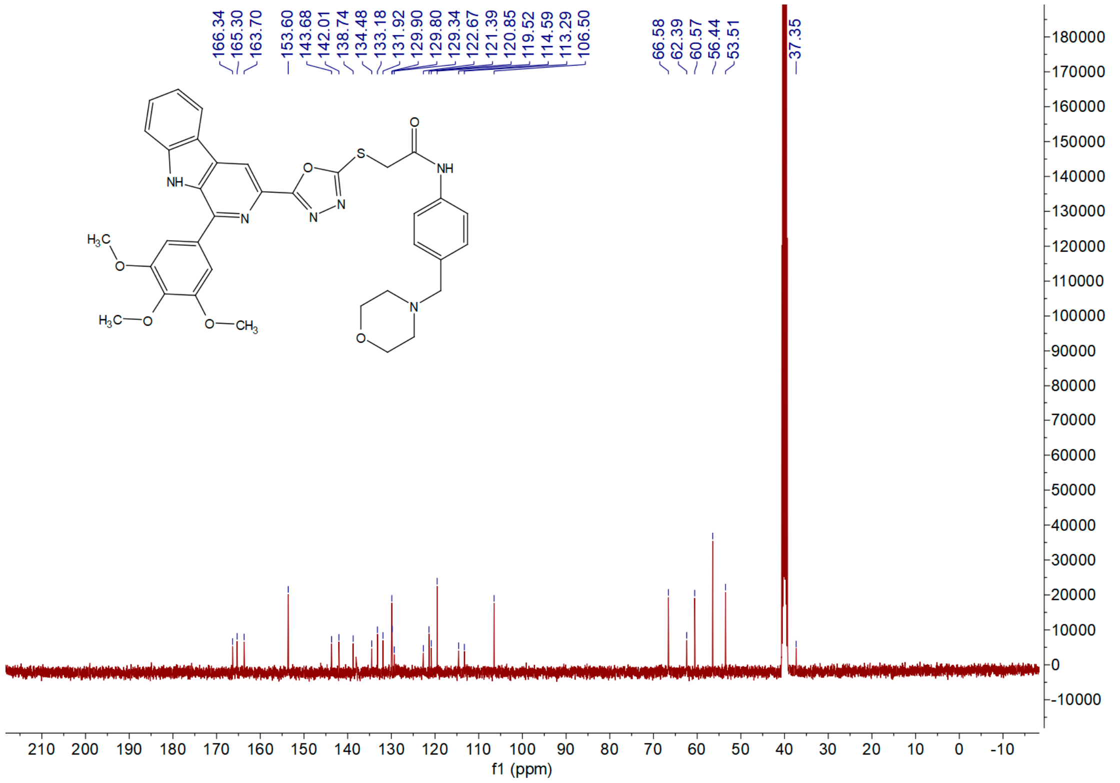

Figure S95 The ^{13}C NMR Spectrum of compound **8q**

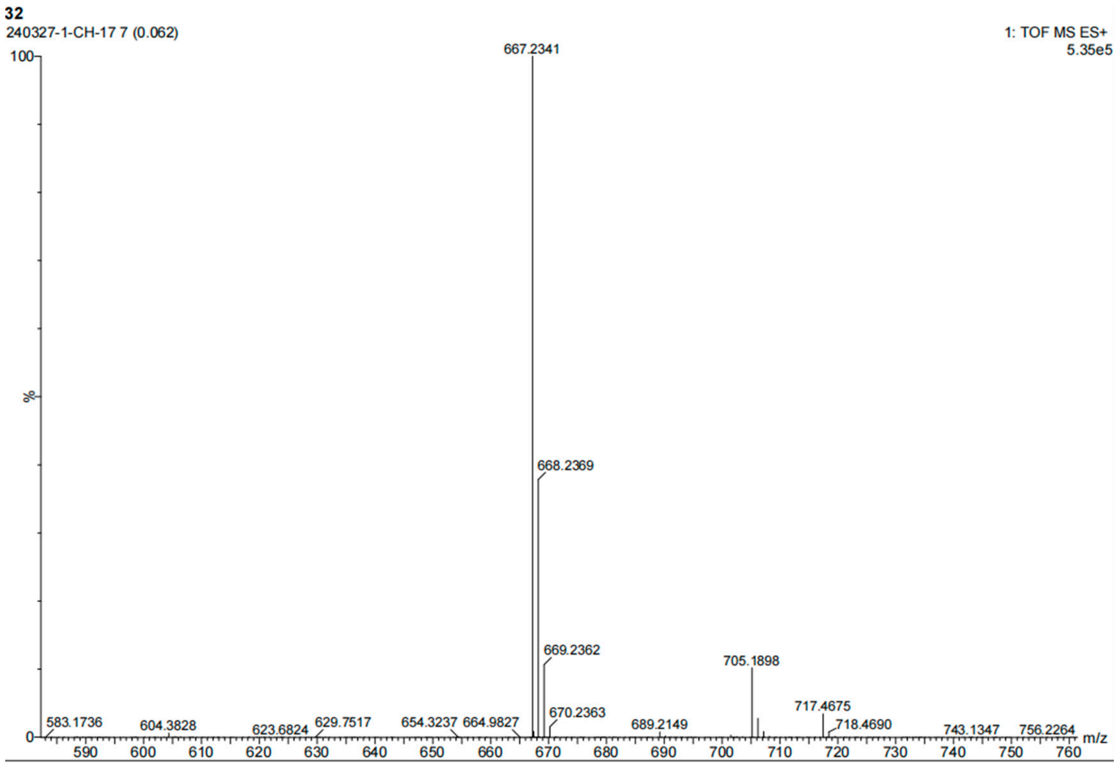

Figure S96 The HRMS spectrum of compound **8q**

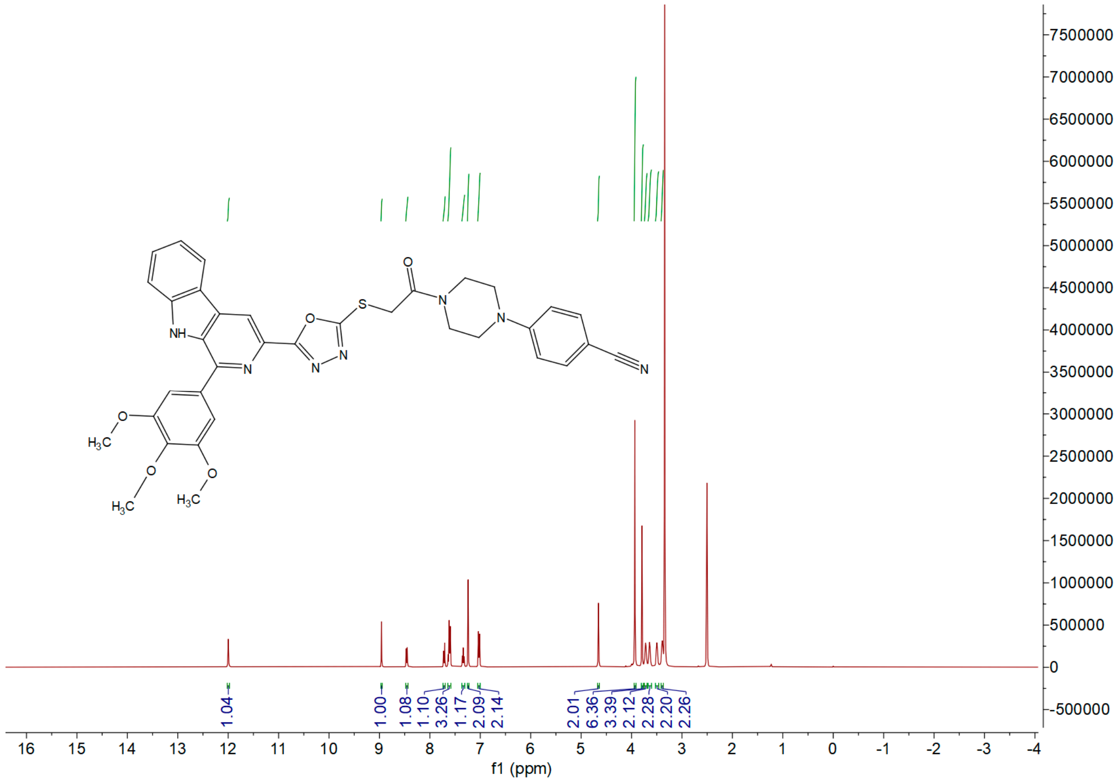

Figure S97 The ^{1}H NMR Spectrum of compound **8s**

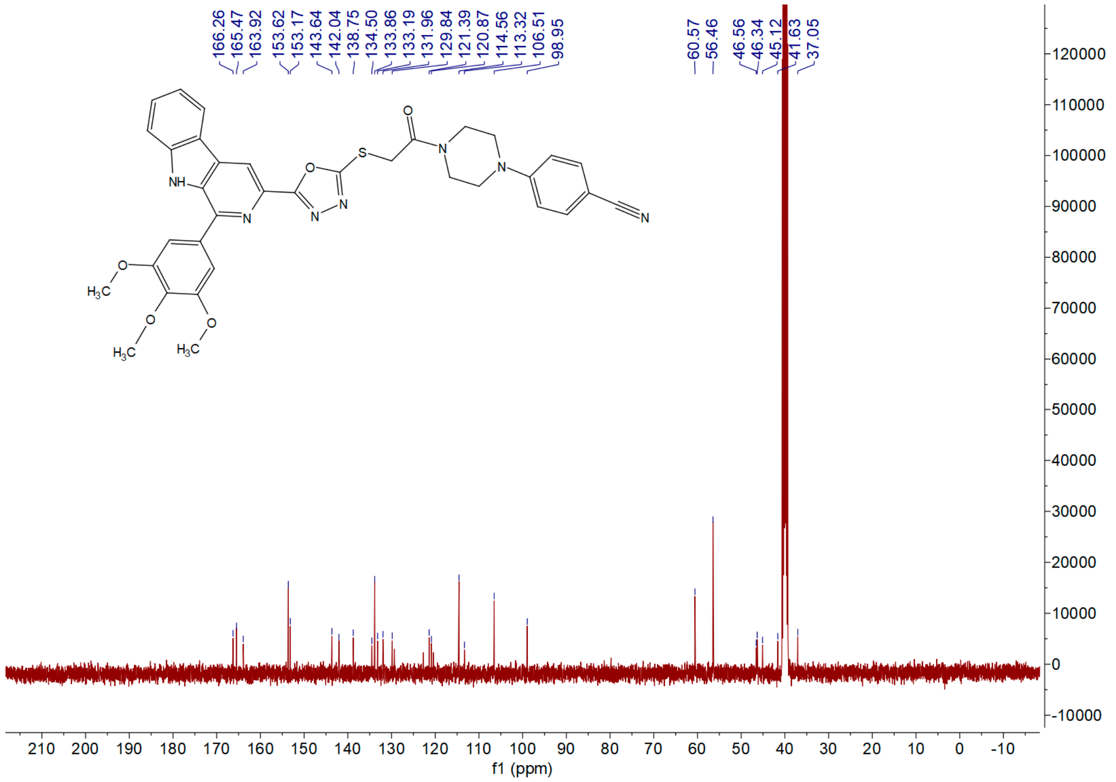

Figure S98 The ^{13}C NMR Spectrum of compound **8s**

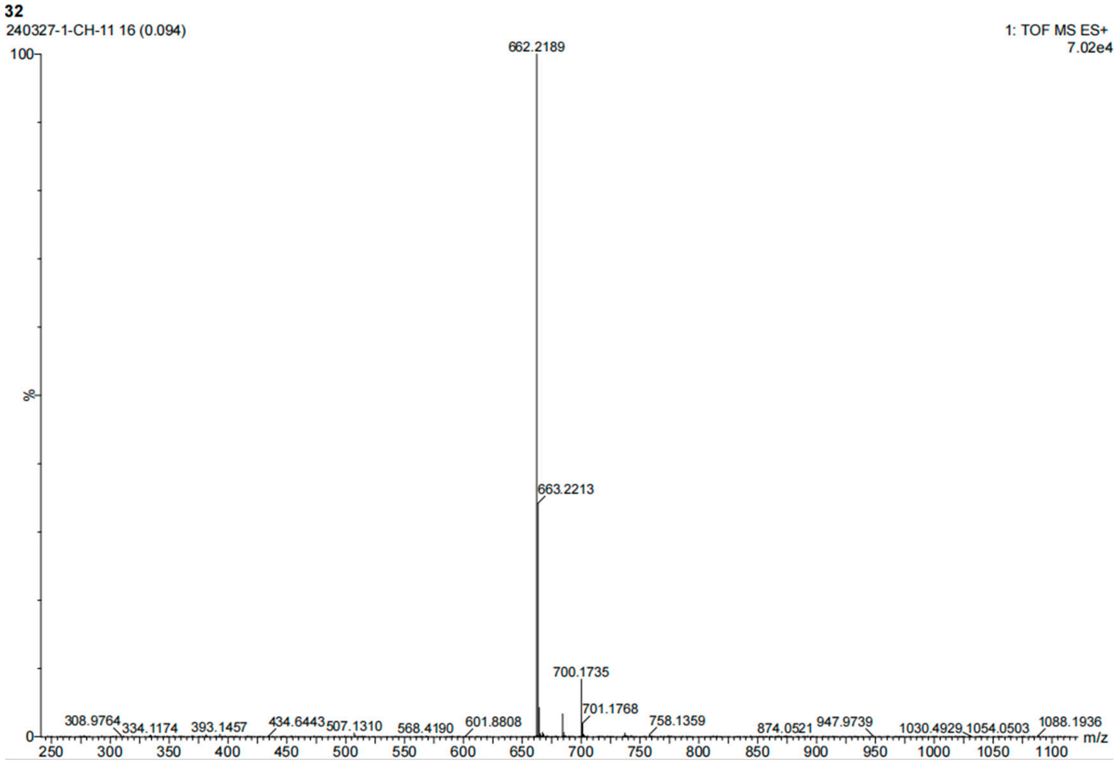

Figure S99 The HRMS spectrum of compound **8s**

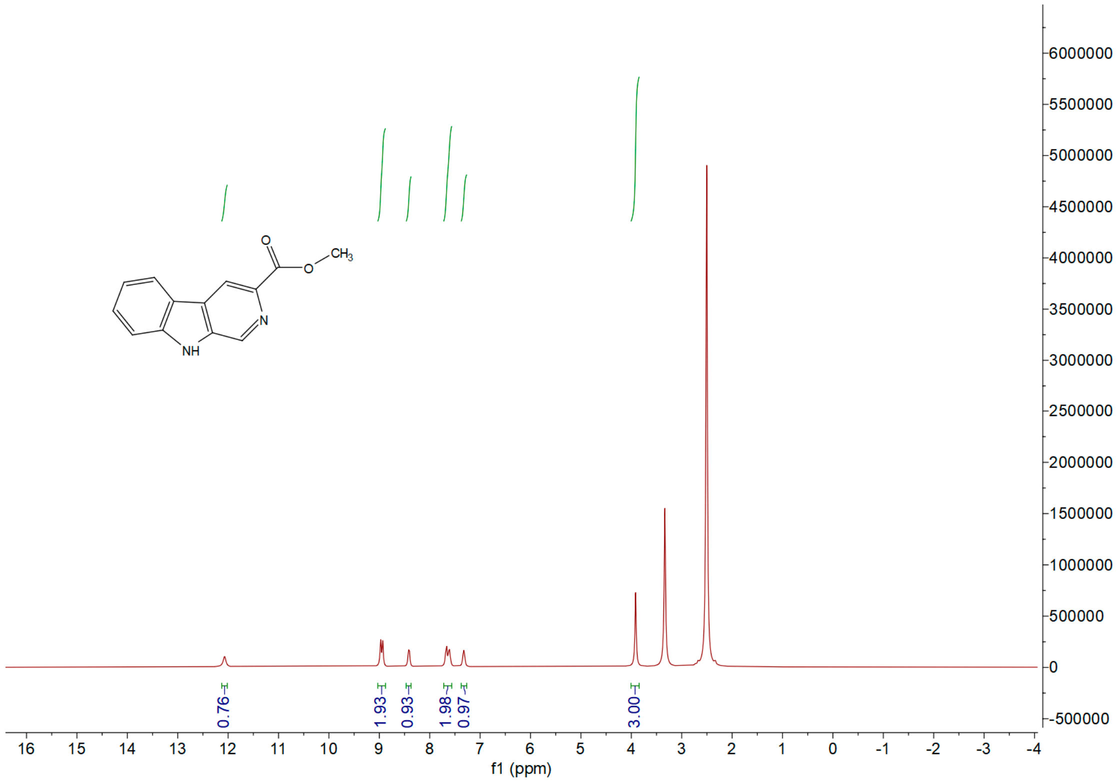

Figure S100 The ^{1}H NMR Spectrum of compound **3a**

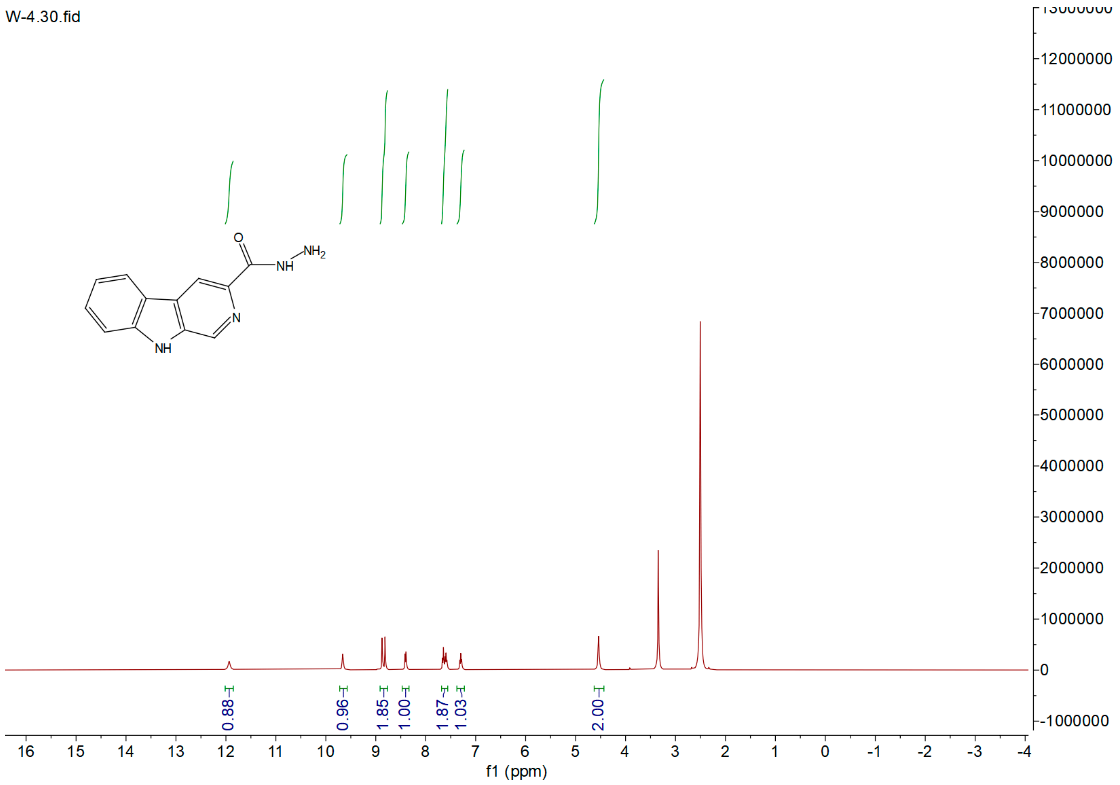

Figure S101 The ^{1}H NMR Spectrum of intermediate **4a**

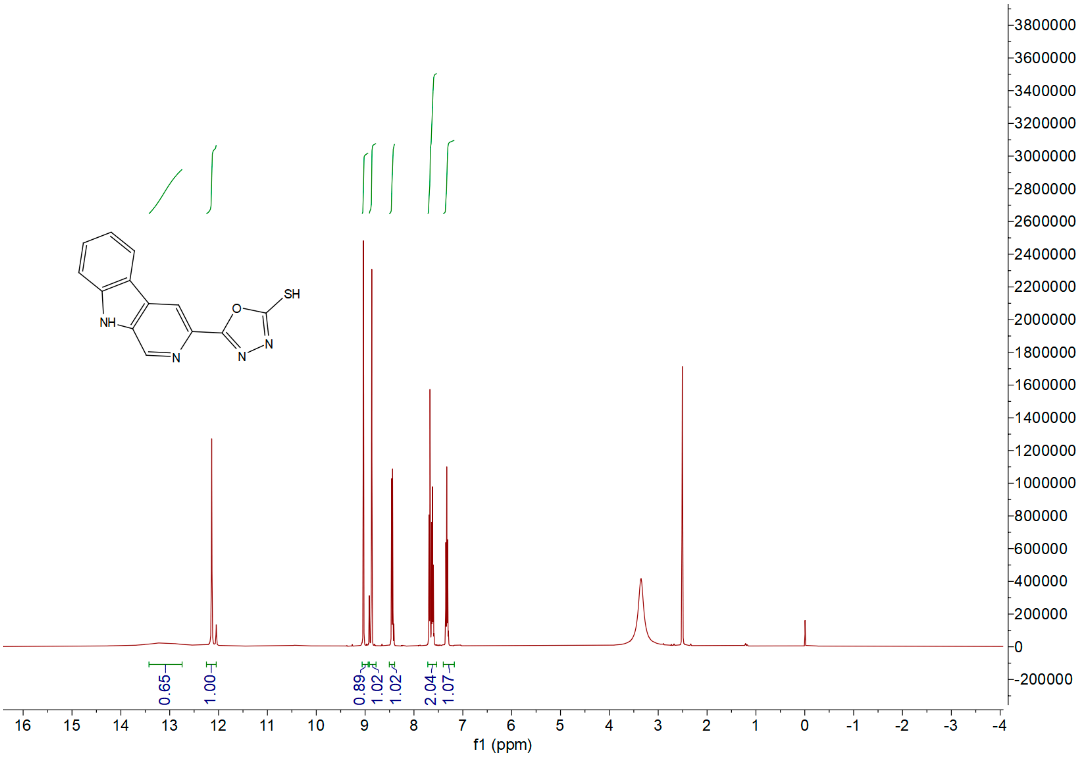

Figure S102 The ^{1}H NMR Spectrum of compound **5a**

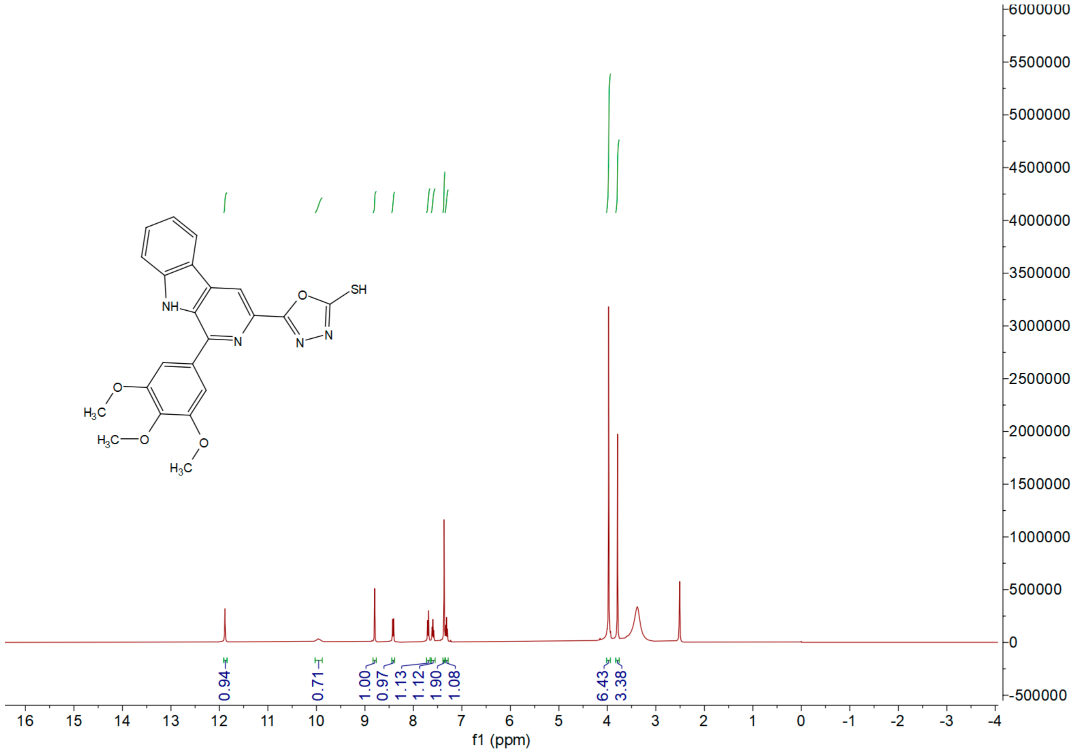

Figure S103 The ^{1}H NMR Spectrum of compound **5b**

1. Zou Y, Zhang G, Li C, et al. Discovery of Tryptanthrin and Its Derivatives and Its Activities against NSCLC In Vitro via Both Apoptosis and Autophagy Pathways. *Int J Mol Sci*. 2023;24:1450.
2. Zhang G, Tang Z, Fan S, et al. Synthesis and biological assessment of indole derivatives containing penta-heterocycles scaffold as novel anticancer agents towards A549 and K562 cells. *J Enzyme Inhib Med Chem*. 2023;38:2163393.
